# Supplementary material for: A breakage–replication/fusion process explains complex rearrangements and segmental DNA amplification
Source: Nat Genet. 2026 Jan 2;58(1):88–99. doi: 10.1038/s41588-025-02434-5 (PMC12807874; doi:10.1038/s41588-025-02434-5)
Supplement: Supplementary file 1 — Supplementary Note and Figs. 1–66. [file 41588_2025_2434_MOESM1_ESM.pdf]

nature genetics

Supplementary information https://doi.org/10.1038/s41588-025-02434-5

A breakage–replication/fusion process explains complex rearrangements and segmental DNA amplification

In the format provided by the authors and unedited

Supplementary Information for

A breakage-replication/fusion process explains complex rearrangements and segmental DNA amplification

Cheng-Zhong Zhang, Carlos Mendez-Dorantes, Kathleen H. Burns, David Pellman

Corresponding Author: Cheng-Zhong Zhang
E-mail: cheng-zhong_zhang@dfci.harvard.edu

This PDF file includes:

SI Figures 1 to 66
References for SI citations

Supplementary Note 5

1 Definitions 6

2 Computational approaches of DNA rearrangement analysis 9
A DNA rearrangement and “structural variant” . . . 9
B Bioinformatic analysis of DNA rearrangement . . . 9
C Evolutionary analysis of *cis* breakpoints and segments . . . 10
D Evolutionary analysis of *trans* segments and breakpoints . . . 10
E Inference of the evolutionary history and mechanism of DNA rearrangement . . . 12

3 Genome observations of insertions and their mechanistic interpretations 16

4 Rearrangement outcomes of break-induced replication 21

5 A proposed mechanism of copy-number gain and amplification from micronucleation 23

6 Footprints of breakage-replication/fusion in L1 clones 26

7 Footprints of breakage-replication/fusion in focal amplifications in the HCC1954 genome 31

8 Translocations and rearrangements of the broken ends of chromosome 4 in bridge clones 40

9 Footprints of breakage-replication/fusion in a post-crisis RPE-1 clone and a bridge RPE-1 clone 42

10 Breakpoints of rearranged segments in bridge clone a 46

11 Segmental structure of rearranged chromosomes in different subclones of bridge clone a 64

12 Foldback junctions in all the subclones of bridge clone a 71

13 Adjacent overlapping breakpoints in chromothripsis in the K-562 genome 73

14 Insertions in single cells after bridge resolution 74

List of Figures

1 Illustration of rearranged DNA, segments, breakpoints, and junctions . . . 5
2 Molecular processes that create rearrangement junctions . . . 7
3 Linkage between breakpoints on duplicated segments . . . 9
4 *cis* breakpoints/segments from DNA fragmentation. . . . 10
5 Timing of breakpoints on duplications. . . . 11
6 Segmental structure of multi-copy gains . . . 13
7 Origin of insertions at rearrangement junctions . . . 16
8 Adjacency between insertions and segmental breakpoints . . . 16
9 Genomic observations of short insertions in the experimental data . . . 17
10 Genomic features of insertions predicted by different models . . . 17
11 Two proposed models for short insertions originating from DNA ends . . . 18
12 Kataegis in insertions and the mechanistic implications . . . 19
13 Breakpoints and rearrangements generated by break-induced replication . . . 21
14 Segregation of fragments of a damaged chromosome from a micronucleus . . . 23
15 Segregation of fragments of a partially replicated chromosome from a micronucleus . . . 23
16 A proposed model of DNA amplification with onion-skin amplicons . . . 24
17 Adjacent parallel breakpoints at L1 ORF2p-induced DSB sites . . . 26
18 Two nested deletions with local sequence inversion in an L1 clone . . . 27
19 Foldback junctions in multi-chromosomal translocations in an L1 clone . . . 28
20 Nested foldbacks at an internal locus in an L1 clone . . . 29
21 Adjacent parallel breakpoints and insertions in L1-induced chromothripsis . . . 29
22 Tiling insertions in L1-induced chromothripsis . . . 30
23 Haplotype-specific DNA copy number and rearrangements of HCC1954 . . . 31
24 Haplotype-specific copy number of chr17 in HCC1954 . . . 31
25 Segmental structure of focal amplifications on chr21 and chr5 in HCC1954 . . . 32
26 Segmental structure of focal amplifications on chr8 in HCC1954 . . . 33
27 Adjacent parallel breakpoints with reciprocal breakpoints in amplified regions in HCC1954 . . . 34
28 Short insertions near the amplified region of chr5:116-117Mb in HCC1954 . . . 35
29 Short insertions near the amplified region of chr5:180-181Mb in HCC1954 . . . 36
30 Examples of nested insertions from amplified regions of chr5 in HCC1954 . . . 37
31 All instances of overlapping insertions in HCC1954 . . . 38
32 Translocations of chr4 break ends in bridge clones from Umbreit et al. (2020) . . . 41
33 Copy number and rearrangement of chr11 in the X-29 clone from Maciejowski et al. (2015) . . . 42
34 Inferred segments of copy-number gains on chr11 in X-29 . . . 43
35 Copy number and rearrangement of chr4 in Primary Clone 5a from Umbreit et al. (2020) . . . 44
36 Phasing of rearrangement breakpoints in Primary Clone 5a based on segmental copy number . . . 45
37 Subclonal copy-number variation in bridge clone **a**, in region A:p-ter–16.5Mb . . . 47
38 Segments of copy-number variation in region A:p-ter–16.5Mb . . . 47
39 Subclonal copy-number variation in bridge clone **a**, region B:17-22Mb . . . 48
40 Segments of copy-number variation in region B:17-22Mb . . . 49
41 Subclonal copy-number variation in bridge clone **a**, region C:24.9-26.3Mb and D:26.7-27.6Mb . . . 50
42 Segments of copy-number variation in region C:24.9-26.3Mb and D:26.7-27.6Mb . . . 51
43 Inference of ancestral DNA fragments from subclonal copy-number variation . . . 51
44 Subclonal copy-number variation in bridge clone **a**, region 30-39Mb . . . 52
45 Segments of copy-number variation in region 30-39Mb . . . 53
46 Subclonal copy-number variation in bridge clone **a**, region 41-46Mb . . . 54
47 Segments of copy-number variation in region 41-46Mb . . . 55

48 Subclonal copy-number variation in bridge clone **a**, region 46-47Mb . . . 56
49 Subclonal copy-number variation in bridge clone **a**, region 48-49Mb . . . 57
50 Segments of copy-number variation in regions 46-47Mb and 48-49Mb . . . 58
51 Subclonal copy-number variation in bridge clone **a**, region R:47-48Mb . . . 59
52 Segments of copy-number variation in region R:47-48Mb . . . 60
53 Subclonal copy-number variation in bridge clone **a**, region J:39-41Mb . . . 61
54 Segments of copy-number variation in region J:39-41Mb . . . 62
55 Segmental structure of rearranged chr4 in subclone **a1, a2, a4** . . . 64
56 Segmental structure of rearranged chr4 in subclone **a3** . . . 66
57 Segmental structure of rearranged chr4 in subclone **a5**, **a6** . . . 67
58 Segmental structure of the ancestral chr4 in bridge clone **a** . . . 68
59 Copy-number of chr4 segments in subclone **a6** . . . 69
60 Secondary chromothripsis in subclone **a6** . . . 70
61 Foldback junctions in all the subclones of bridge clone a . . . 71
62 Rearrangements of the 4B homolog in subclones **a2**, **a4**, and **a5** . . . 71
63 Adjacent overlapping breakpoints on chr18 in the K-562 genome . . . 73
64 Tiling pattern of insertions in the C-2a daughter cell from Umbreit et al. (2020) . . . 74
65 Tiling pattern of insertions observed in both daughter cells of the C-4 mother cell from Umbreit et al. (2020) . . . 74
66 Tiling pattern of insertions in the T-1a daughter cell from Umbreit et al. (2020) . . . 75

Supplementary Note

We start by defining commonly used terminologies related to DNA rearrangement with examples shown in **SI Figure 1** and then describe new computational approaches for DNA rearrangement analysis.

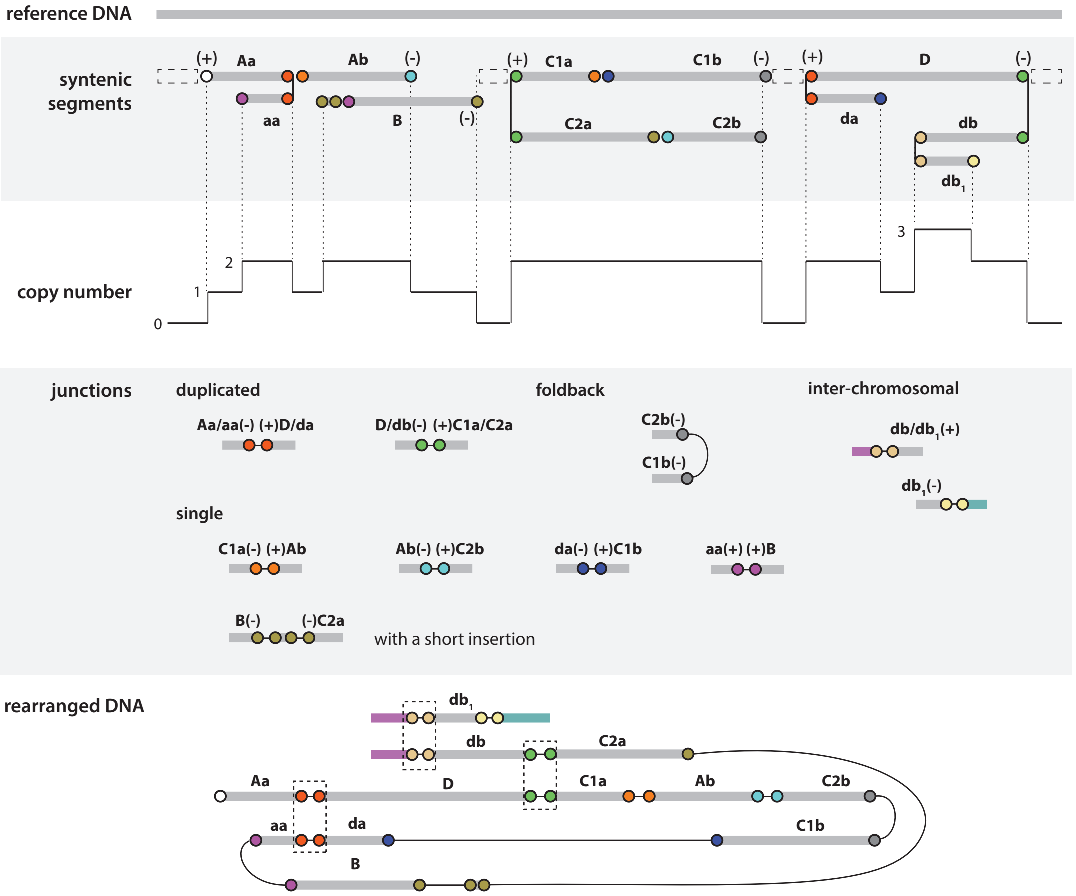

SI Figure 1: An example of rearranged DNA (bottom) with representations of segments (gray bars), breakpoints (open circles), and copy number on the reference DNA (top). The naming of segments follows the same convention as for the bridge clone shown in **Extended Data Figure 4**. (+) and (-) denote the directionality of copy-number transition across the breakpoint. Junctions (colored circles) between breakpoints determine the order of rearranged segments in the rearranged chromosome. Dashed boxes in the rearranged DNA structure represent duplicated junctions.

1. Definitions

(i) **Rearrangement** A DNA sequence consisting of two or more subsequences (**Segments**) that are conserved in another (reference) sequence. See DNA rearrangement and "structural variant" and Bioinformatic analysis of DNA rearrangement.

(ii) **Segment** A subsequence in the reference DNA that is conserved (i.e., contiguously aligned) in the rearranged DNA. Gray bars in **SI Figure 1**. See Segment and synteny mapping for further discussion.

(iii) **Breakpoint** The boundaries of rearranged segments define breakpoints (open circles in **SI Figure 1**). Breakpoints are represented by locations in the reference DNA. (+) and (-) denote the direction of **copy number** transition at a breakpoint.

(iv) **Copy number** The copy number of a sequence from the reference DNA is the total number of segments from a rearranged chromosome or genome that are mapped to this locus. DNA copy number only takes integer values. But the bulk average DNA copy number in a heterogeneous population may take non-integer values, which is the algebraic average of the integer copy-number states weighed by their subclonal fractions.

(v) **Duplicated segment (duplication)** A sequence from the reference genome is duplicated in the rearranged genome if its copy number is larger than one. Duplicated sequences may be contained in more than one segment with different breakpoints, or multiple copies of the same segment. For a heterozygous genome, only sequences derived from the same parental chromosome are considered to be duplications, but not those from different parental (homologous) chromosomes.

(vi) **Junction** A DNA subsequence in the rearranged DNA that connects two rearranged segments (spans two breakpoints). These are shown as color-filled circles in **SI Figure 1**. Also see **SI Figure 2** below.

(vii) **DNA fragments and DNA ends** A DNA fragment is a single-stranded or double-stranded DNA molecule and has two termini, or DNA ends. See Breakpoints and DNA ends for further discussion.

(viii) **Adjacent breakpoints** Adjacent breakpoints are those in close proximity in the reference DNA such that they are unlikely to have been generated independently by chance.

(ix) **Insertion** A short DNA sequence inserted between large segments in the rearranged DNA. A provisional criterion for distinguishing between insertions and segments is that insertions are flanked by *adjacent* (+) and (-) breakpoints. See Insertions and segments for further discussion. .

(x) **Phasing of breakpoints and junctions** To phase breakpoints is to determine their linkage on the segments of rearrangement. For examples of breakpoint phasing, see **Sec. 7**, **10** and **9**. To phase junctions is to determine their linkage and order in the rearranged chromosome(s). See **Sec. 11** for examples.

(xi) ***cis* and *trans* breakpoints** *cis* breakpoints are derived from DNA ends from a single ancestral chromosome or DNA fragment; *trans* breakpoints are derived from DNA ends from different (duplicated) copies of a chromosome or DNA fragment. For duplicated segments generated by aberrant replication (see **SI Figure 2C and F**), the breakpoints of each duplication are in *cis* and the breakpoints on different duplications are in *trans*.

(xii) ***cis* and *trans* segments** The phase of segments can refer to either their origin, i.e., whether they are derived from DNA fragments from the same or different ancestral chromosomes, or their locations in the rearranged genome, i.e., whether they are preserved in the same or different rearranged chromosomes. Here we use *cis* or *trans* segments to refer to their origin: *cis* segments are derived from fragments of a single ancestral chromosome by chromosome *fragmentation*. *trans* segments originate from different ancestral chromosomes. *cis* segments can be identified by *adjacent* (-) and (+) breakpoints derived from reciprocal DNA ends after DNA fragmentation (cf. **Figure 1** of the main text). *trans* segments can be identified by the presence of partial overlap between two segments. See Evolutionary analysis of *cis* breakpoints and segments and Evolutionary analysis of *trans* segments and breakpoints for further discussions.

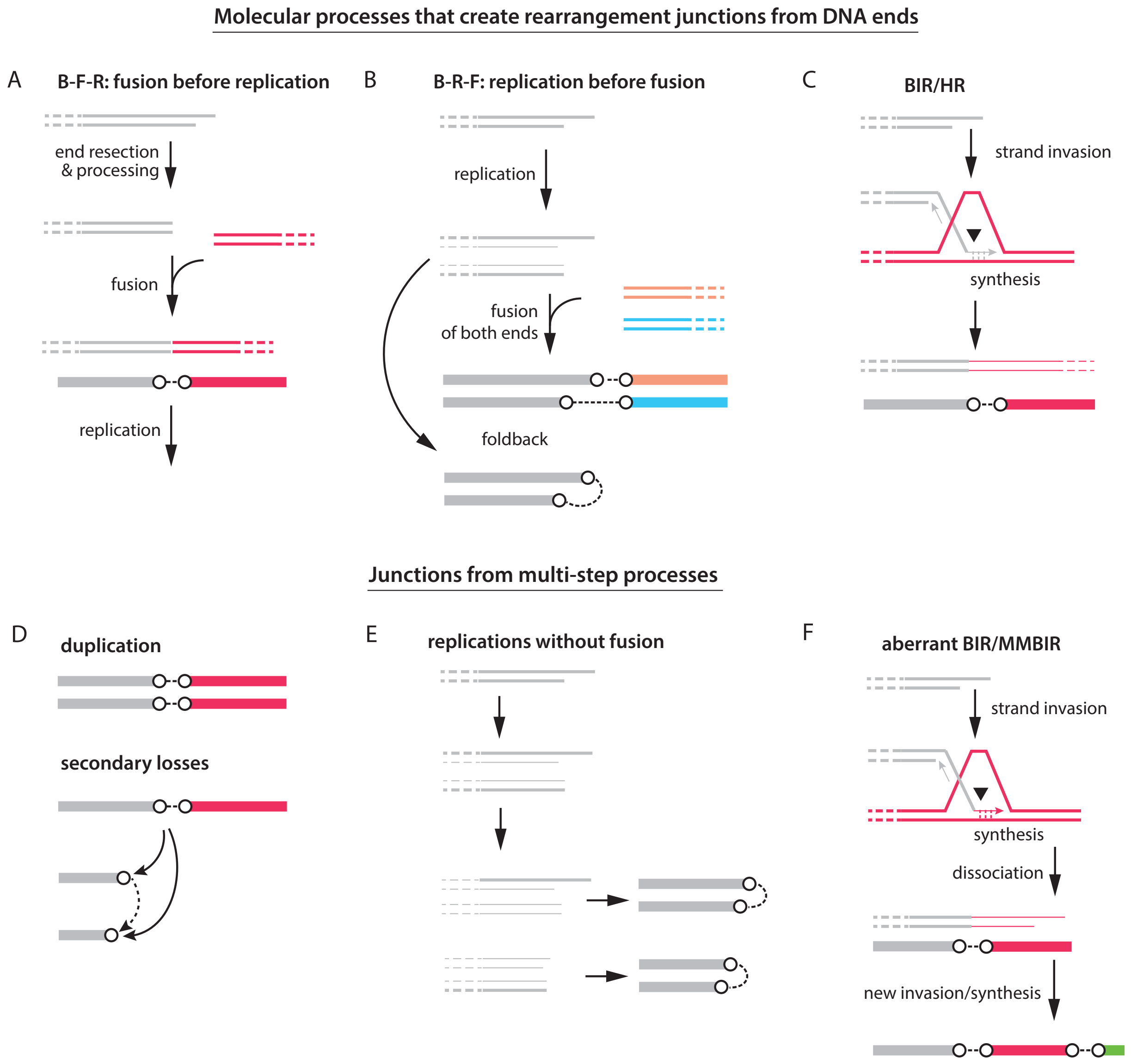

SI Figure 2: Molecular processes that create rearrangement junctions.

A. Breakage-fusion-**replication** (B-F-R). Fusion between two DNA ends can create a single junction with two breakpoints. Each breakpoint originates from a single ancestral dsDNA end.

B. Breakage-**replication**-fusion (B-R-F). A dsDNA end is first duplicated by replication, and the sister DNA ends then form separate junctions. A special case is when sister DNA ends join each other to form a foldback junction. Newly synthesized DNA strands are shown as thin lines; template DNA strands are shown as thick lines.

C. A dsDNA end invades an intact DNA template and initiates synthesis by break-induced replication (BIR). See reviews by Anand et al. (3) and Kockler et al. (13). BIR may be resolved in different ways, but the junction created by the initial strand invasion event consists of two breakpoints, one originating from a dsDNA end, the other from strand invasion. See Rearrangement outcomes of break-induced replication.

D. Junctions/breakpoints can be duplicated or deleted by downstream events.

E. Replication of a free DNA end over multiple generations can create two or more adjacent DNA ends as seen in regions of DNA amplification, as shown in **Figure 2** of the main text.

F. Microhomology-mediated break-induced replication, or MMBIR, posits that the BIR fork can dissociate from one template (red) and invade a different template (green), creating a series of junctions by “copy-and-paste.”

Note that breakage-replication/fusion (**A** and **D**), break-induced replication (**C**), or microhomology-mediated break-induced replication (**F**) can produce similar breakpoint/junction patterns, but adjacent parallel breakpoints are a specific feature of breakage-**replication**-fusion (**B** and **E**).

Additional discussion of segments, breakpoints, and junctions of rearranged DNA

Segment and synteny mapping. Rearranged segments are identified by aligning the rearranged sequence to the reference sequence. **Synteny** (blocks of genes with conserved order) mapping can be regarded as a special case of segment determination when alignment is performed at the gene level. Synteny mapping is used when comparing the genomes of *different species*, where significant sequence divergence interferes with the identification of long-range segmental conservation. For *somatic cells* from the same individual, the segments of rearrangement can be directly identified by sequence-level alignment. However, the alignment should distinguish between segments derived from different homologous chromosomes based on the *haplotype phase*. For duplicated sequences that are highly similar, their rearrangement can only be determined based on the non-duplicated flanking sequences. An example can be found in the determination of duplicated segments in 39-41Mb in bridge clone **a**.

Breakpoints and DNA ends. A DNA end is a *transient* molecular structure of a DNA fragment. A breakpoint describes a feature of the *rearranged DNA sequence*. There are several key differences between breakpoints and DNA ends. First, a double-stranded (ds)DNA end consists of two single-stranded (ss)DNA ends that can be either flush (same position for both strands) or staggered (different positions on opposite strands); a breakpoint is a sequence feature that is identical for both DNA strands. Second, DNA ends are not duplicated exactly due to the end-replication problem or end processing such as resection; breakpoints are sequence features that are duplicated exactly. Finally, breakpoints can arise from dsDNA ends, but can also arise from an intact DNA donor template that is invaded by a dsDNA end. See **SI Figure 2**. In the second scenario, the breakpoint corresponds to the site of invasion or template switching, but not a DNA end. Given the uncertainty about the origin of individual breakpoints, an integrative analysis of all the breakpoints is necessary to infer whether a breakpoint originates from DNA breakage or strand invasion, and to determine the relationship between the breakpoint and the ancestral DNA end.

Insertions and segments. Putative insertions are derived from sequences not from the host genome, such as viral DNA or integrated transgenes. Insertions are also used to refer to short sequences sandwiched between large segments, even if the inserted sequences originate from the host genome. An example is a short segment (unlabelled) to the left of segment **B** in **SI Figure 1**.

An insertion is referred to as a 'templated' insertion if the inserted sequence can be mapped to one or multiple locations in the reference (in the latter case, these sequences represent repeats). However, there is no difference between templated insertions and segments: Any subsequence that can be mapped to a location in the reference DNA should be viewed as a rearranged segment regardless of its size.

Here, we use insertions to refer to short sequences for which the breakpoints are closer than expected for random breakpoint generation (i.e., adjacent breakpoints). The adjacency between breakpoints suggests they are generated in a *concerted* manner, i.e., at the same time. By contrast, the two breakpoints of a large segment may arise from independent events occurring at different timepoints. In bridge clone **a**, insertions and large segments fall into distinct size ranges (**Extended Data Fig. 6c**. The distinction between short insertions and large segments based on their size differences is supported by their distinct origins in the ancestral DNA (**Figure 6** and **SI Figure 8**) and their distinct joining patterns in the rearranged chromosome (**Extended Data Figure 5** and **Sec. 11**).

Insertions are sometimes used to refer to *all* rearranged segments inferred to have been generated by a concerted process, e.g., "chains of insertions" inferred to be generated by a "copy-and-paste" process (14). However, even if we infer that each inserted sequence is generated in a concerted process based on breakpoint proximity, this does not imply that all the insertions in a junction are generated in a single concerted process. An important implication of the breakage-replication/fusion model is that free DNA ends can persist over one or multiple generations. Therefore, it is possible that a chain of insertions arises from concatenation of DNA fragments generated by different mechanisms (see **SI Figure 10**). More generally, the inference of the origin of rearranged DNA segments (including short insertions) should be separate from the inference of the mechanism that concatenates these segments together.

Rearrangement process and mechanism. To create a rearranged DNA molecule or chromosome usually requires a series of events including DNA breakage ('cut'), DNA repair/recombination ('paste'), and DNA duplication ('copy'). We consider a rearrangement process or mechanism to be a cascade of events that occur in a specific order (e.g., chromosome fragmentation followed by end-joining) and generate an outcome with specific signatures (e.g., oscillating copy number). See Inference of the evolutionary history and mechanism of DNA rearrangement for further discussion.

2. Computational approaches of DNA rearrangement analysis

A. DNA rearrangement and "structural variant". A structural variant is a representation of the rearranged sequence as an *alteration* of the reference sequence (i.e., a variant). Common (simple) structural variants include deletions, duplications, inversions, transpositions, and translocations. There are also patterns of complex structural variants that cannot be reduced to combinations of simple structural variants (14). In comparison to the structural variant representation, the representation of rearrangement using segments and junctions have several advantages.

First, segments and junctions can be uniquely defined for any rearranged sequence based on its alignment to the reference sequence, but the identification of complex rearrangement patterns (e.g., local n-jumps, chromothripsis etc.) can be heuristic and often requires operational thresholds (14).

Second, the rearranged sequence can be uniquely determined from the segments and junctions of a rearrangement, but a complex structural variant can represent more than one rearranged sequence. See Figure 1 of Li et al. (14) for examples. This ambiguity is because structural variants are defined solely based on the joining pattern of breakpoints (i.e., junctions), but do not include breakpoint linkage information as illustrated in **SI Figure 3**.

Third, breakpoints and segments can be related to ancestral DNA ends or DNA fragments, for which the evolutionary timing and correlation can be inferred by statistical analysis (e.g., adjacent breakpoints). By contrast, a structural variant involves two or more breakpoints from different segments that may have different evolutionary timing. This ambiguity becomes a considerable challenge for the evolutionary timing inference of complex rearrangements with many breakpoints.

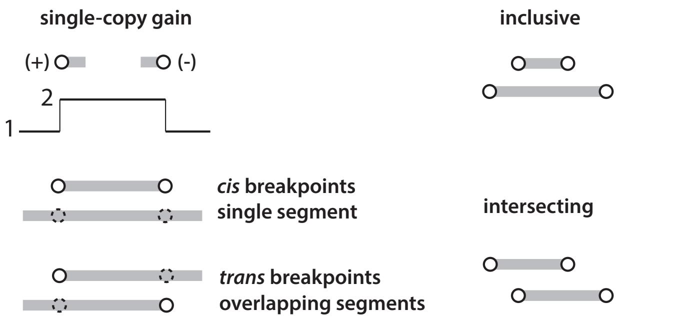

SI Figure 3: Breakpoint linkage on overlapping rearranged segments. *Left*: *cis* and *trans* breakpoints associated with a single-copy gain. *Right*: Two segmental configurations reflecting different *cis* breakpoints.

Finally, breakpoints and segments provide more insight into the mechanism of rearrangement than structural variants. For example, tandem duplication (TD), a common structural variant, can arise in different sizes (21) and by different molecular processes. It has been demonstrated that 10kb TDs are generated by the replication-bypass mechanism associated with BRCA1 deficiency (38), which produces a net gain of DNA. However, this mechanism does not produce larger TDs (>100kb or >1Mb). Moreover, as demonstrated both in cancer genome analysis (28, 29) and in the current study (**Extended Data Figure 8**), replication bypass can also create duplications associated with two translocations. Therefore, the central genomic feature of the replication-bypass mechanism is the presence of two DNA segments with a 10kb overlap, but not a head-to-tail joining pattern of breakpoints as indicated in the TD classification.

The common usage of structural variants for DNA rearrangement analysis is at least partially due to the technical limitation that shotgun sequencing reads can only resolve junctions between breakpoints, but not their linkage in the rearranged DNA. With long-read sequencing and Hi-C sequencing, we can determine the linkage between breakpoints and resolve the segmental structure of rearranged DNA. Due to reasons listed above, it is advantageous to build a general framework for DNA rearrangement analysis based on the segmental structure instead of the conventional representation based on structural variants.

B. Bioinformatic analysis of DNA rearrangement. A complete description of DNA rearrangement includes three features: the rearranged segments, the junctions between segments, and the order of junctions and segments in the rearranged chromosome. Among these features, junctions can be efficiently detected from shotgun sequencing reads with non-contiguous (discordant) alignments to the reference. From the aligned subsequences of these reads, we can further identify breakpoints and segments that are shorter than the read length. Finally, in the absence of DNA duplication, all the breakpoints are unique to a single chromosome, and the segments can be determined from consecutive (+) and (-) breakpoints on each chromosome.

When there are duplicated segments, determination of their segmental structure requires knowledge of the linkage between breakpoints on each segment (**SI Figure 3**). For small duplications, the breakpoints can be directly resolved from a single sequencing read or from a unitig (a unique path on the assembly graph) assembled from sequencing reads. For large DNA duplications (≥100kb) with little sequence variation (the average frequency of de novo substitutions in a somatic cell is usually $\lesssim 10^{-5}$), even long reads cannot resolve the linkage between breakpoints. In the current study, we have demonstrated two strategies to determine the long-range linkage (100kb or above) between breakpoints: The first is to identify *cis* breakpoints that

have the same copy number, as illustrated in the resolution of Breakpoints of rearranged segments in bridge clone **a**. The second is to identify *cis* breakpoints that form de novo *Hi-C contacts* with the same translocation partner, which is demonstrated in the analysis of amplified DNA in the HCC1954 genome (**SI Figure 25** and **26** in **Sec. 7**). A third strategy that is not used in the current study is Strand-Seq. By combining these approaches, it is in principle feasible (as demonstrated here for bridge clone **a**) to determine the segmental structure of duplicated or amplified DNA, which addresses a major problem in the assembly of complex cancer genomes.

C. Evolutionary analysis of *cis* breakpoints and segments. For rearranged DNA that is preserved in a clonal population, it is impossible to definitively exclude the possibility that two segments or two breakpoints originate from two ancestral DNA molecules (e.g., sister chromatids). However, two strategies may be employed to infer *cis* breakpoints or *cis* segments indirectly.

The first strategy is by the identification of adjacent gapped or overlapping breakpoints (main text **Figure 1**). The *cis* relationship is established by two lines of reasoning: (1) the proximity between these breakpoints suggests that the probability that they are generated independently on different DNA molecules is very small (an evolutionary argument); (2) there is ample evidence that such breakpoints can result from reciprocal DNA ends created by a single DNA breakage event (a molecular argument). For adjacent breakpoints inferred to have been generated at the same time, their partners in the rearrangement junctions should also be generated at or around the same time. This strategy can therefore identify evolutionarily concurrent breakpoints that are non-adjacent in the original chromosome.

As an example, we consider three segments as shown in **SI Figure 4**. For the proximal breakpoints on segment 1 and 2, if the breakpoint distance d_1 is very small compared to the size of the segments (L_1 and L_2), we can infer that the two breakpoints are generated from a single breakage event ($p_1 \ll 1$). Similar argument can be applied to breakpoints between segment 2 and 3 (when $p_2 = d_2/(L_2 + L_3) \ll 1$). However, these two inferences do not imply that all three segments are generated at the same time: it is possible that the two pairs of adjacent gapped breakpoints are generated by DSBs at different timepoints. If the two adjacent breakpoints between segment 1 and 2 are joined to breakpoints between segment 2 and 3, then we can infer that the two DSBs occur at the same time. The same inference can be drawn if size of the middle segment is very small (i.e., it is a short insertion) compared to the flanking segments, $p_{12} = L_2/(L_1 + L_3) \ll 1$.

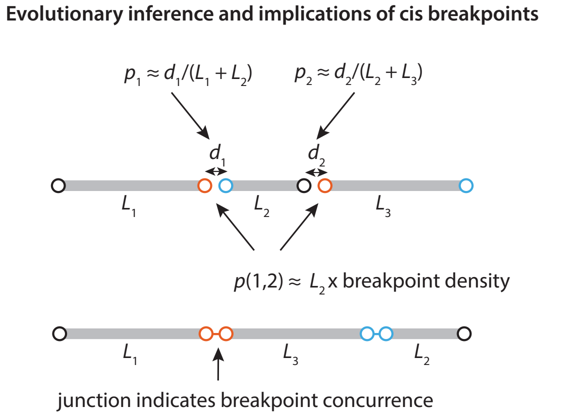

SI Figure 4: *cis* breakpoints/segments from DNA fragmentation.

When there is no duplication (i.e., overlap between segments) in the rearranged chromosome, we can assign all breakpoints and segments to a single ancestral chromosome based on parsimony: The alternative model that some breakpoints/segments are derived from two different ancestral chromosomes implies a duplication followed by extensive segmental losses. This line of reasoning is weaker than the inference based on adjacent breakpoints and it also does not constrain the timing of breakpoints.

A well-known example of *cis* segments is in chromothripsis, where all the segments are derived from fragments generated in a single catastrophe. The oscillation between deletion and retention suggests all the retained segments are derived from the same ancestral chromosome, but does not establish that they are generated all-at-once. The argument that the rearrangements in chromothripsis arises all-at-once instead of by gradual evolution was originally made by contrasting the simulated copy-number outcomes of gradual evolution with the observed two-state oscillating copy-number pattern (31). A counterargument to this original argument was made subsequently by Kinsella et al. (12). We later provide a revised argument supporting the all-at-once model of chromothripsis in Inference of the evolutionary history and mechanism of DNA rearrangement.

D. Evolutionary analysis of *trans* segments and breakpoints. Here we only consider *trans* segments/breakpoints on duplicated segments. For simplicity, we do not consider pre-existing duplications in the reference chromosome and assume all the segments of the rearranged chromosome are mapped to unique locations in the reference. We want to infer (1) the phylogenetic relationship between duplicated segments; (2) the timing of breakpoint formation on duplicated segments. These inferences can be drawn based on three types of variation that distinguish different duplicated copies: (1) local sequence differences; (2) different breakpoints; (3) different flanking sequences.

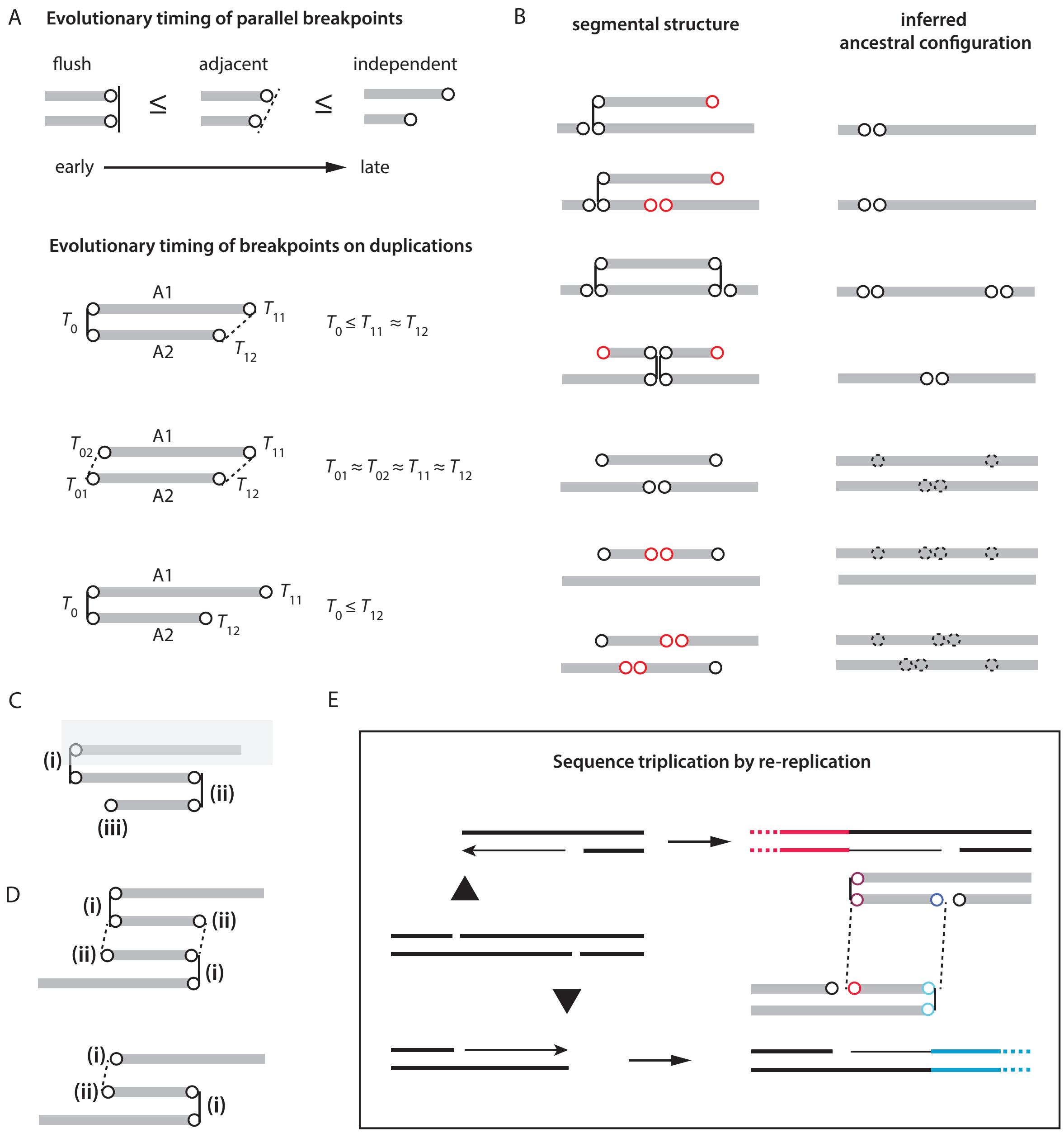

SI Figure 5: Timing of breakpoints on duplications.

A. Relative timing of parallel breakpoints on duplications.

B. Segments of a single-copy gain (left) and the inferred ancestral segments (right). Ancestral breakpoints are shown as black circles; secondary breakpoints are shown as red circles. The top four segmental configurations can be derived from fragments of a single ancestral chromosome; the bottom three can only arise from two ancestral chromosomes each with a subset of breakpoints (dashed circles).

C. An example of a non-flush breakpoint (i) that is generated before the flush breakpoints (ii). The long breakpoint (i) is initially duplicated but becomes a single breakpoint after deletion of the long segment (shaded box). Note that the short breakpoint (iii) must be generated after the flush breakpoint.

D. A special scenario when adjacent parallel breakpoints (i and ii) are generated sequentially by two rounds of replication as shown in **E**.

E. The replication forks originating from the middle segment collapse at the nicks on opposite strands, creating adjacent ssDNA nicks and dsDNA ends. Re-replication (through replication forks fired from the fusion partners in magenta and blue colors) can create up to two internal segments with adjacent breakpoints on both sides (**D**, top). A subsequent deletion can result in one pair of adjacent breakpoints plus one pair of flush breakpoints (**D**, bottom), but not two pairs of adjacent breakpoints or two pairs of flush breakpoints.

Mutations The evolutionary timing of duplicated segments can be inferred from the number of mutations generated after duplication. These mutations should accrue independently in each duplicated copy (e.g., assessed by the positions of mutations). As the average frequency of de novo mutations is generally low ($\lesssim 10^{-5}$), the most useful scenario is when there is hypermutation (e.g., kataegis). See **SI Figure 12** and **31** for examples.

Breakpoints The following rules apply to breakpoints on duplicated segments (**SI Figure 5A**). (1) Each breakpoint is generated only once at a specific timepoint (a direct consequence of the infinite-sites model of genome evolution; see Ma et al. (17). (2) Flush breakpoints (identical position and identical junction sequence) are generated before duplication. (3) Adjacent parallel breakpoints are generated by replication through a single unligated dsDNA end; therefore, they are generated at the same time as the (initial) duplication. Most adjacent parallel breakpoints are staggered, but they may have identical positions by chance (but have different junction sequences). (4) For two segments that share a flush breakpoint, the non-flush breakpoint on the shorter segment must have arisen after flushed breakpoints. (Note that this does not hold for the non-flush breakpoint on the longer segment, as shown in **SI Figure 5C**). The relative timing of breakpoints on duplicated segments can be summarized as:

$$T[\text{flush breakpoints}] < T[\text{replication}] = T[\text{staggered breakpoints}] \leq T[\text{short non-adjacent breakpoint}].$$

Applying these constraints to single-copy gains with various breakpoint configurations (**SI Figure 5B**), we can infer the relative timing of duplication and breakpoint generation. The example in **SI Figure 5C** shows why the timing constraint applies only to the short breakpoint but not the long breakpoint when both are in *cis* with flush breakpoints.

Segments Two overlapping segments are either 'inclusive' or 'intersecting' (**SI Figure 3**). These two configurations have different implications on the evolutionary relationship between the segments. (1) For two segments in an inclusive configuration (i.e., one is contained in another), either the shorter segment is a descendent of the longer segment, or the two segments evolve independently from two different chromosomes. These two scenarios cannot be distinguished. (2) For two segments in an intersecting configuration (i.e., with a significant but partial overlap), they must have been generated independently from two ancestral DNA molecules. Examples of these two scenarios are shown in **SI Figure 6**. (3) For two segments with identical breakpoints, they arise from a secondary duplication of a single ancestral segment; the timing of the duplication can be inferred from mutations generated after the duplication or from the breakpoints of their flanking segments (see this example).

In general, higher segmental copy-number states are associated with more breakpoints on both sides of the amplification. If the amplified DNA segments are generated independently, either by fragmentation of duplicated chromosomes or by BIR using an intact donor chromosome, the segments of amplified DNA can be either inclusive or intersecting. The rarity of intersecting segments seen in both bridge clone **a** (**Extended Data Figure 4**) and in the HCC1954 genome disfavors these mechanisms and supports an origin of amplified DNA generated by iterative breakage-replication/fusion cycles.

Note: The two constraints about inclusive or intersecting segments are both due to the constraint that a breakpoint can be *shortened* but not *extended*. DNA ends can be extended by homologous recombination (HR); however, HR can only generate one breakpoint that extends the ancestral DNA end, but cannot create a new breakpoint that extends an existing breakpoint.

E. Inference of the evolutionary history and mechanism of DNA rearrangement. The question of how a rearranged DNA sequence arises is often addressed from two angles. The first is to infer the evolutionary history of rearrangement, i.e., to deduce the series of sequence alterations that create the rearranged DNA sequence from the reference sequence. The second is to infer the mechanism of rearrangement, i.e., infer the initiating and downstream events including DNA breakage, DNA repair, and DNA replication that can explain all the features of the rearranged DNA.

Considerable efforts have been put into the inference of the evolutionary trajectory that produces a specific pattern of DNA rearrangement, including simulations (14). Here, we show that one can draw specific evolutionary or mechanistic inferences about DNA rearrangement from the sequence features, i.e., breakpoints, junctions, and segments of rearranged DNA, without knowledge of the exact evolutionary trajectory of the rearranged DNA sequence.

As an example, we revisit the genomic evidence that suggests rearrangements in chromothripsis arise in a single catastrophe instead of by gradual evolution. The original argument by Stephens et al. (31) was based on the prediction that a random series of deletions, tandem duplications, or inversions as inferred from the junctions of the rearranged DNA will produce DNA duplications. This prediction contradicts the observation that the rearranged chromosome only shows two copy-number states, retention and deletion. A caveat in this otherwise elegant argument is that it implicitly assumes head-to-tail junctions

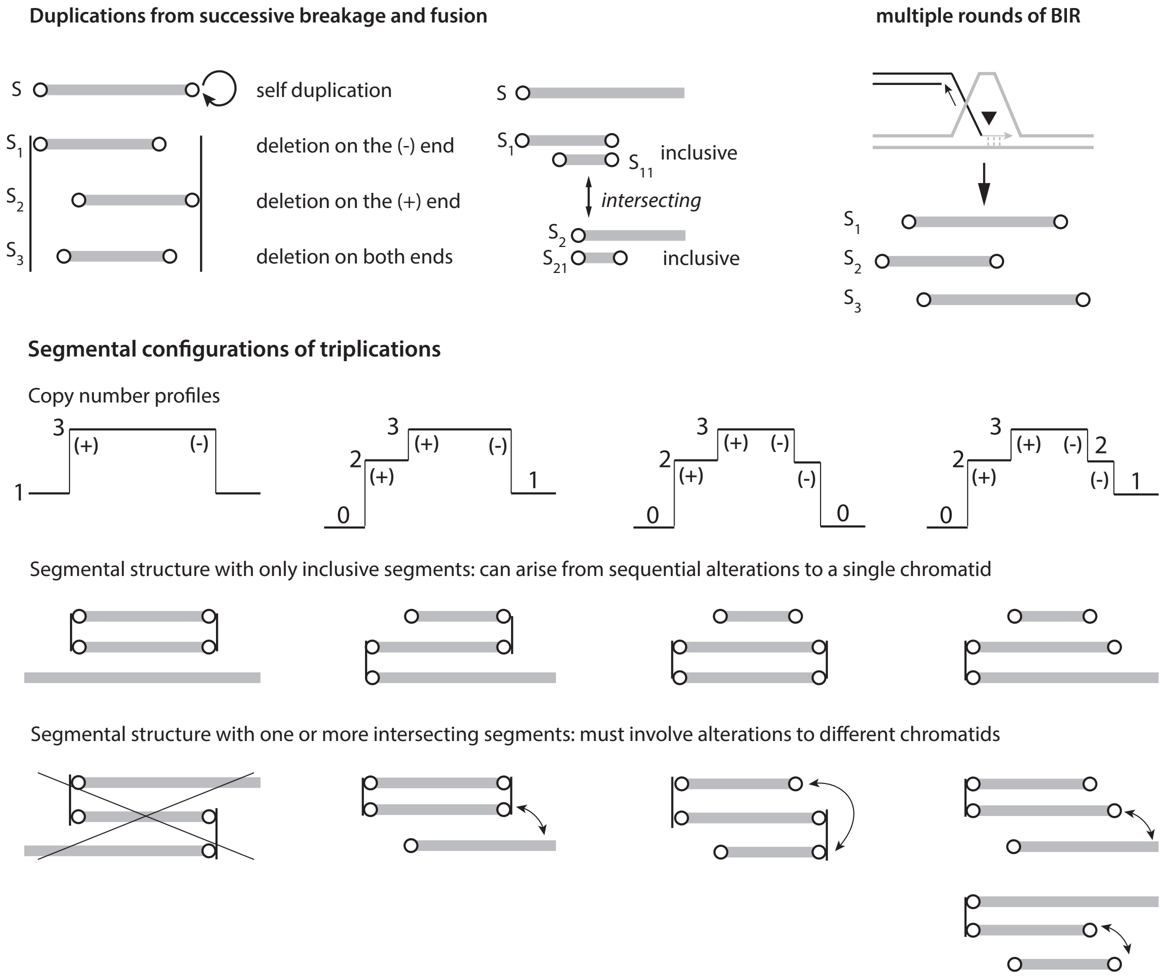

SI Figure 6: Segmental structure of multi-copy gains.

Top left: Configurations of breakpoints on secondary duplications generated by breakage and fusion. Each secondary duplication is shorter than its ancestor ("inclusive"); two segments with partial overlap ("intersecting") must have descended from different ancestors (different strands of a single dsDNA fragment, or independent dsDNA segments).

Top right: Duplications generated by break-induced replication (BIR) from an intact donor template can be partially overlapping as the breakpoints generated in different rounds of BIR are independent.

Bottom: Examples of segmental configurations of triplications. The first row show configurations without partially overlapping segments; the second row show configurations with partially overlapping segments. The constraint of no partial overlapping often determines a unique segmental configuration. Note that the first example of triplication is not permitted based on the model shown in **SI Figure 5E**.

to reflect tandem duplications and omits the possibility that such junctions can also arise from complex alterations that do not cause copy-number gains, such as two inversions. If we accept these alternative interpretations of the junctions, then it is straightforward to come up with a series of inversions and deletions, or a series of transpositions and deletions, that produce only deletions but not duplications, as suggested by Kinsella et al. (12). Therefore, the argument for chromothripsis as a one-off catastrophe hinged on one of many possible interpretations of rearrangement junctions.

Here we provide an evolutionary argument for the one-off model of chromothripsis that is solely based on features of the rearranged DNA (segments, breakpoints and junctions) but does not invoke the "structural variant" interpretation of rearrangement junctions.

First, the concentration of breakpoints on a single chromosome cannot be explained by random DNA breakage that is expected to affect all chromosomes. Such concentration can only arise from an *unstable* chromosome. The question is whether these breakpoints accumulate over multiple generations (cell division) or in one generation.

Second, by definition, an unstable chromosome cannot be stably inherited by both daughters. If the unstable chromosome is

replicated completely but unevenly segregated between the daughter cells, it should create both copy-number gains and losses. Note that this argument for copy-number gains based on uneven DNA segregation is different from the original argument where duplication was derived from an interpretation of the head-to-tail junction.

Third, if the predominance of deletion in both daughters reflects deficient replication of the unstable chromosome, then the breakpoints of each deletion (due to deficient replication) are evolutionarily concurrent. Note that this argument only applies to breakpoints of each deletion, but does not imply that all the segmental deletions are generated all-at-once.

Finally, the formation of junctions between the breakpoints of different deletions establishes that these breakpoints are generated concurrently. Therefore, the copy-number, breakpoint, and junction features of chromothripsis suggests a process that creates multiple deletions in one generation.

We can extend this analysis to complex rearrangements with copy-number gains. We first consider a *single* duplication. The duplicated DNA is contained in two segments that can be either inclusive or intersecting. If the segments are intersecting (i.e., with partial overlap), they must have arisen independently; if the segments are inclusive (i.e., one segment is contained in the other), then there is the additional possibility that the short segment is derived from a duplicated copy of the long segment. For segments generated by fragmentation of both sister chromatids (15), we expect at least some segments are intersecting and some are inclusive. The absence of intersecting segments in bridge clone **a** therefore disfavors this model. The same reasoning can be applied to complex duplications in cancer or congenital disease genomes, including complex amplifications. The inclusive pattern of amplified DNA segments that is reminiscent of the "onion-skin" structure suggests a plausible mechanism of DNA amplification after a chromosome undergoes multiple rounds of partial replication.

Here we first discuss additional Genome observations of insertions and their mechanistic interpretations. We then discuss the Rearrangement outcomes of break-induced replication and their possible connection to Translocations and rearrangements of the broken ends of chromosome 4 in bridge clones observed in the bridge clones. Finally, we propose a new mechanism of copy-number gain and amplification from micronucleation that may explain complex focal amplifications in the HCC1954 genome.

3. Genome observations of insertions and their mechanistic interpretations

In this section, we discuss the genomic features of insertions and their mechanistic implications. Insertions at rearrangement junctions are observed in both cancer genomes and experimentally generated rearrangements. (See the 4th paragraph in **Discussion** of the main text for references.) A popular interpretation for insertion rearrangements is that the inserted sequences arise from template-switching DNA synthesis (**SI Figure** 7, top), or "copy-and-paste" (14). Insertion junctions can also arise from ss- or dsDNA fragments that are ligated between DNA ends. Several possible mechanisms that can generate short DNA fragments that become insertions are shown in **SI Figure** 7, bottom.

SI Figure 7: Origin of insertions at rearrangement junctions

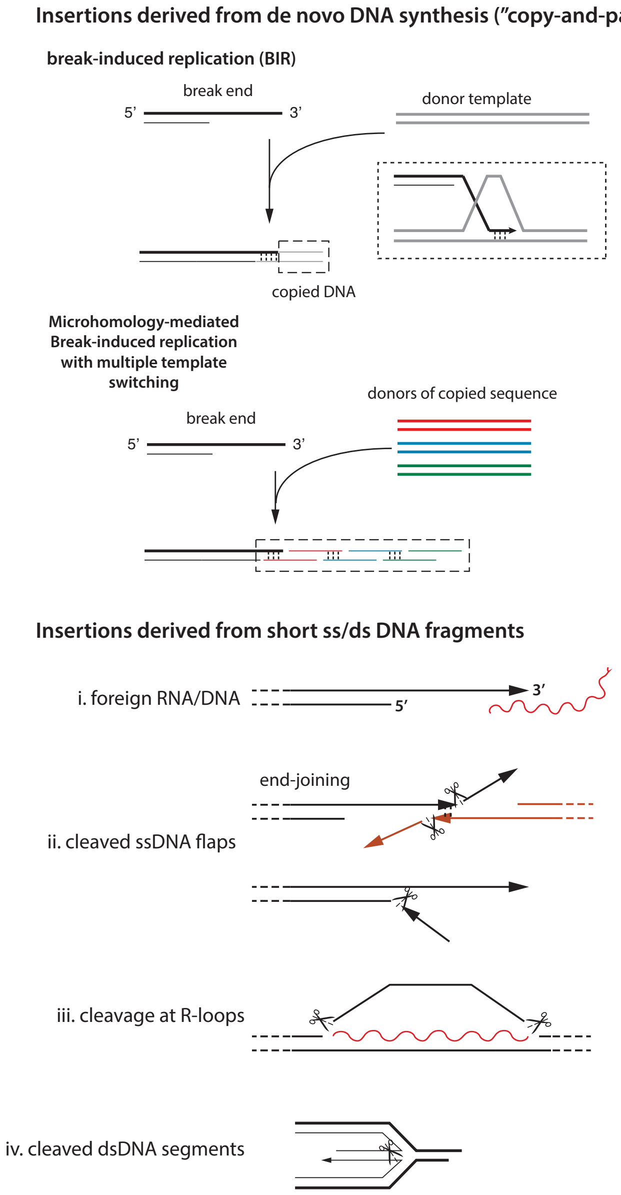

SI Figure 8: Adjacency between insertions and segmental breakpoints

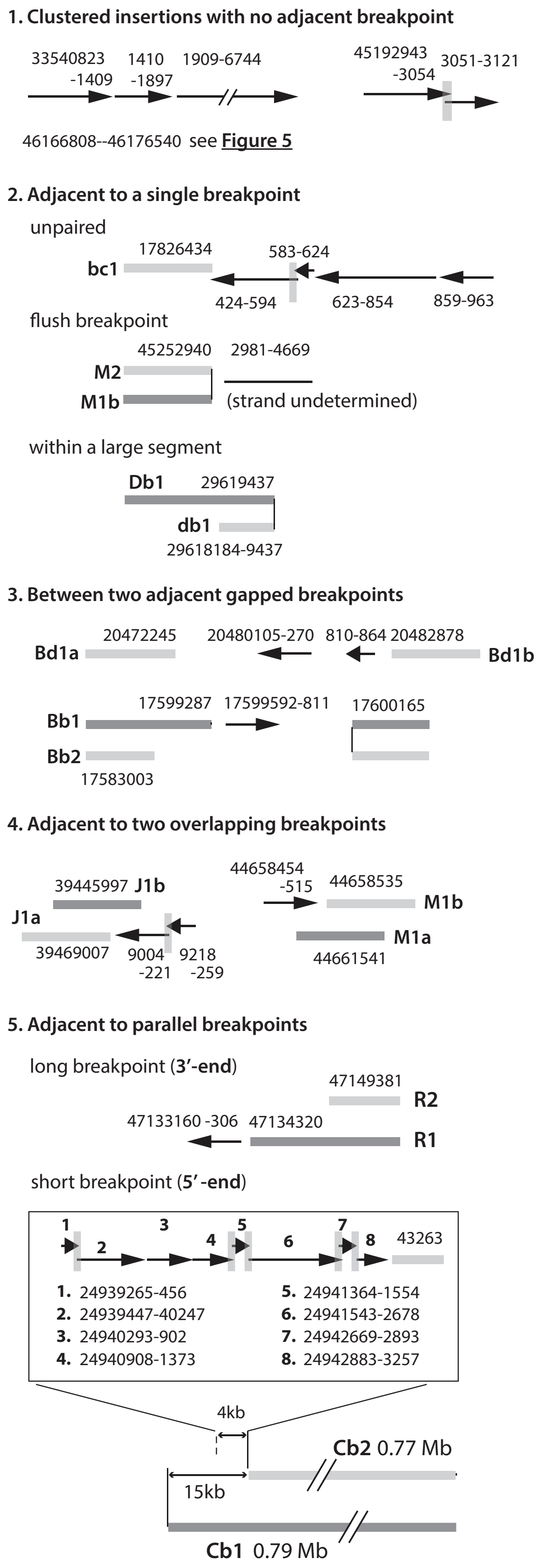

The first source of insertions is RNA or reverse transcribed cDNA (i). The integration of such sequences at rearrangement junctions between dsDNA ends was first described by Moore and Haber (25) and Teng et al. (34); the authors further showed that non-homologous end-joining was involved in the generation of insertion junctions. The second and third sources of insertions are cleaved ssDNA flaps, e.g., during microhomology-mediated end-joining repair (ii, top), DSB resection (ii, bottom), or in R-loops (iii). Such cleavage processes have been experimentally demonstrated, but the insertion of the cleaved DNA into rearrangement junctions has not. Finally, Yu et al. (39) suggested that insertions can also originate from cleaved ssDNA or dsDNA fragments at reversed replication forks. For the last mechanism, neither the cleavage nor the insertion has been demonstrated experimentally.

Based on the adjacency between the original sites of insertions and large segmental breakpoints, we can classify the insertions identified in bridge clone **a** into five categories as shown in **SI Figure 8**: (i) Single or clustered insertions not adjacent to large segmental breakpoints. (ii) Insertions adjacent to one large segmental breakpoint, including insertions that share a breakpoint with a large segment (**db1/Db1**) as described below. (iii) Insertions between two gapped breakpoints. (iv) Insertions adjacent to one of two overlapping breakpoints. (v) Insertions adjacent to two parallel breakpoints. In (ii)-(v), the ancestral strand of the insertion can be inferred from the polarity of the ancestral DNA ends of the nearby segmental breakpoints. For example, the **bc1** breakpoint is inferred to have been derived from an ancestral 5'-end (cf. **Extended Data Fig. 7a**); therefore, the insertions are inferred to have been derived from fragments of reverse strand DNA.

The most remarkable feature of insertions is when they form tiles at the original sites (**Figure 5** and **SI Figure 8**). Similar patterns are also observed in single cells as discussed in **Sec. 14**.

The insertions and their junctions show several features (**SI Figure 9**) that contradict predictions of "copy-and-paste" processes. Consider the model that insertion junctions are generated from sequential strand invasions into a ssDNA donor (**SI Figure 10**, left) as proposed by Min et al. (22), this model can partially explain insertion hotspots (ssDNA templates), but not the tiling pattern of the inserted sequences. Moreover, this model predicts strict strand coordination of the inserted sequences, which is clearly violated in almost all junctions in bridge clone **a** (**Figure 5**).

SI Figure 9: Features of short insertions observed in bridge clone **a**

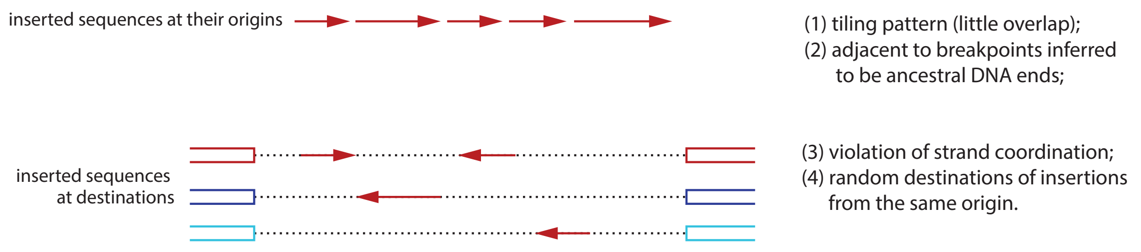

SI Figure 10: Features of short insertions predicted by copy-and-paste or end-joining models

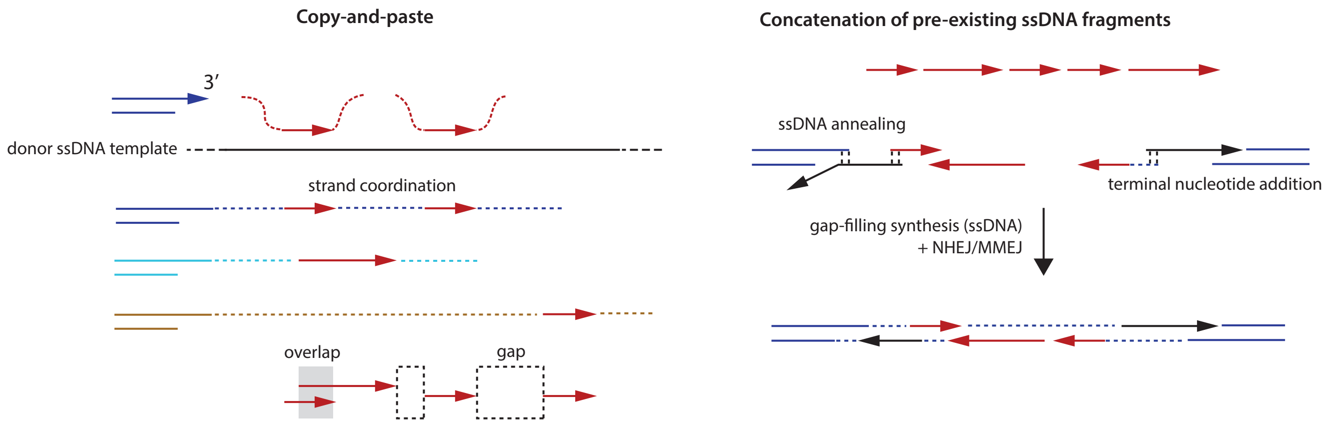

Both the tiling pattern of insertions at their origins and the strand alternation between insertions at the destination junctions can be explained if the insertion junctions are generated by concatenation of pre-existing ssDNA fragments (**SI Figure 10**, right).

Direct annealing between ssDNA fragments based on microhomology will create insertions with strand alternation. ssDNA annealing can also prime DNA synthesis that converts ssDNA fragments into dsDNA fragments, which can then be ligated at either orientation. Further support for this model is provided by the observation of complex insertion junctions in L1 clones (20) consisting of both L1 and genomic DNA sequences derived from sites of large segmental breakpoints.

There are two outstanding questions about the model shown in **SI Figure 10**, right. First, what processes generate discontinuous ssDNA fragments near DNA ends, most commonly the 5'-ends of resected DSBs, and how. Second, how are these fragments freed (displaced) from the template DNA from which they are derived? We suggest that free ssDNA fragments may arise from two possible mechanisms shown in **SI Figure 11**.

SI Figure 11: Two proposed models for the generation of short insertions originating from regions near resected DNA ends

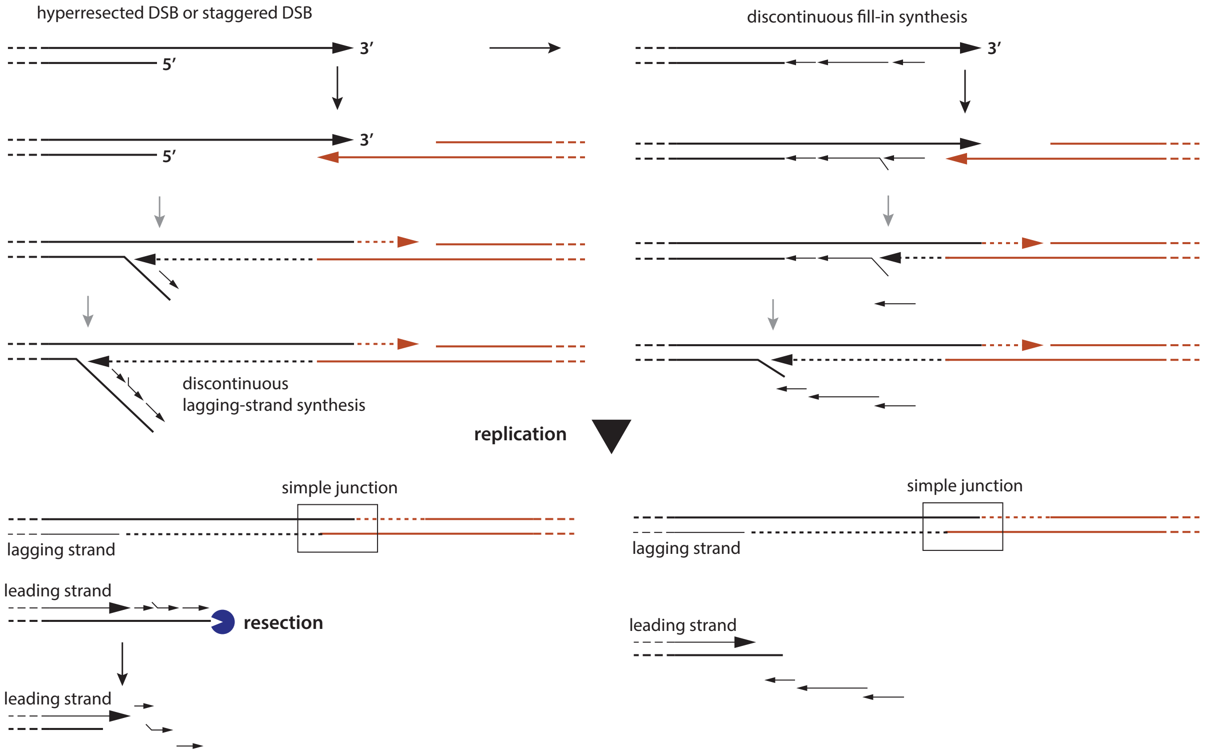

In the first model (shown on the left), strand-displacement DNA synthesis displaces the 5'-end and initiates lagging-strand synthesis. Subsequent 5'-resection releases the newly synthesized Okazaki fragments as free ssDNA fragments. Alternatively, endonuclease cleavage can fragment the lagging strand DNA into small dsDNA fragments. In the second model (shown on the right), discontinuous ssDNA fragments are generated first and then displaced from the template strand. Both models require significant strand displacement activity from strand-displacement synthesis that could be due to polδ and co-factors in a similar capacity as in homologous recombination (19) or polθ-mediated end-joining (33).

In both models, the synthesis of discontinuous ssDNA fragments may be initiated by CST (CTC1, STN1, TEN1) and its associated DNA polα/primase. See reviews by Mirman and de Lange (24) and Mirman et al. (23). In vitro assays suggested that CST preferentially binds ssDNA at the junction with dsDNA (5). If this were true in vivo, fill-in synthesis would preferentially start from regions near the 5'-ends of resected DSBs, explaining the adjacency between the insertions and the breakpoints inferred to be ancestral 5' DNA ends. As many insertions exceed 100bp, and some insertions that share a breakpoint with a large segment can reach 20kb (e.g., **SI Figure 29**C), the synthesis must involve a long-processivity polymerase such as polδ or polϵ.

In normal lagging strand synthesis, Okazaki fragments are ligated by LIG-I or LIG-III after flap removal by the endonucleases FEN1 or DNA2. The persistence of these fragments may be explained by the deficiency of these enzymes (e.g., in an abnormal nuclear environment such as micronuclei or bridges). The observation that DNA2 deficiency promotes the generation of insertion junctions in budding yeast (39) is consistent with this model.

The hypothesized process of long-tract ssDNA synthesis during DSB repair is supported by several observations of insertions

that share breakpoints with large segments as shown in **SI Figure 12**.

SI Figure 12: Kataegis within insertions and a proposed model for insertions that share a breakpoint with large segments.

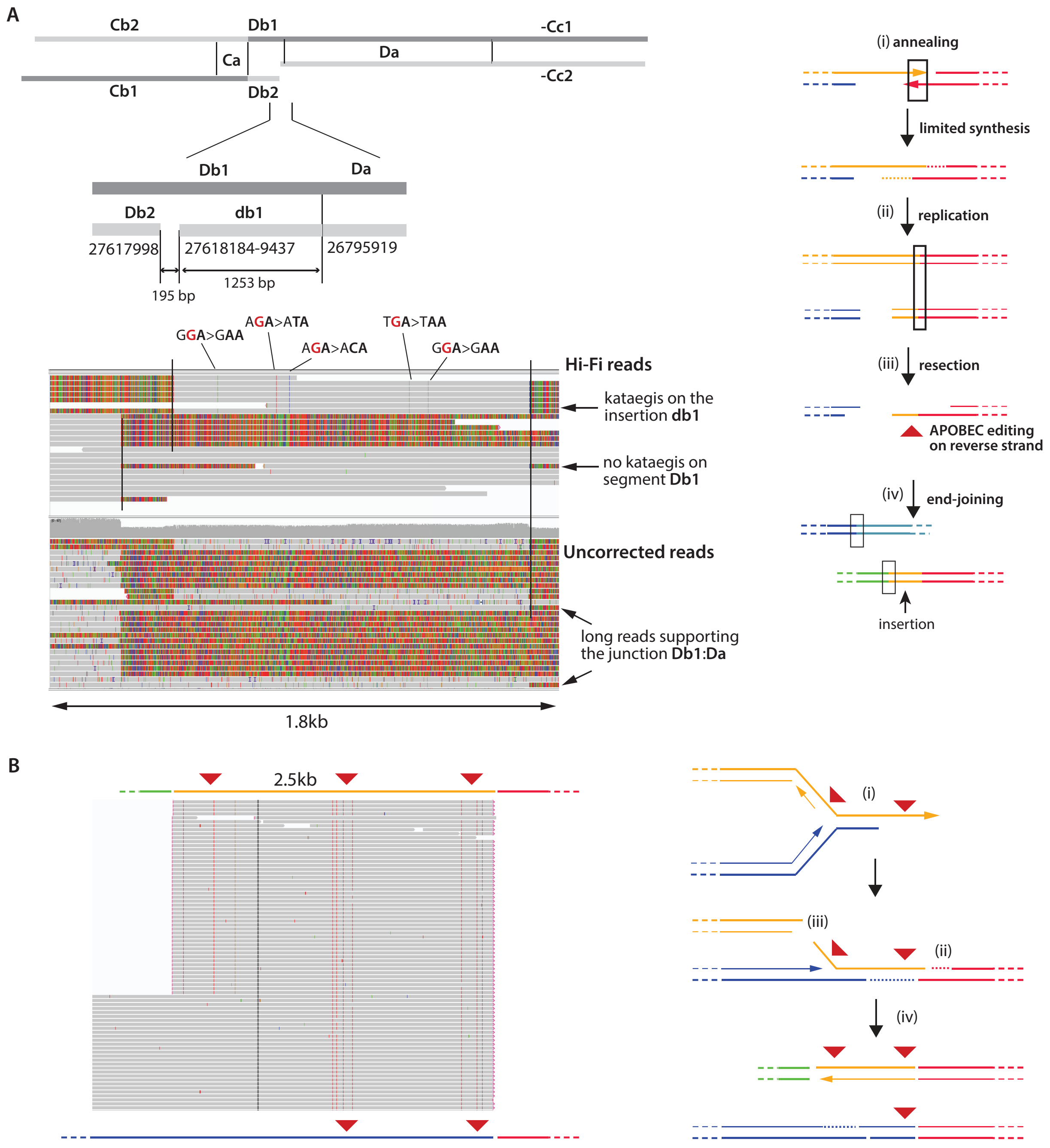

In **SI Figure 12A**, we show the **db1** insertion at its original site. This insertion shares the minus breakpoint with the **Db1** segment. Based on the structure of compound duplications consisting of segments **Cb1/Cb2**, **Ca**, **Db1/Db2**, **Da**, and **Cc1/Cc2**, we infer the **db1** segment to be derived near the 3'-end of reverse-strand DNA. This inference is validated by the presence of five G>A substitutions on the **db1** insertion but not on the larger **Db1** segment. Based on these observations, we propose the following model for this insertion shown on the right.

(i) Two staggered DNA ends anneal with each other followed by gap filling synthesis; the top strand is fully ligated but the bottom strand has a gap due to hyperresection of the left DNA end (dark blue).

(ii) DNA replication converts the gap on the bottom strand into two dsDNA ends.

(iii) Newly created ends are resected and undergo deamination. Note that the strand of deamination is opposite to the original DNA overhang. This is further supported by two substitutions (TGA>TCA and AGA>ACA) on the left end of **Da** that are restricted to the copy of **Da** linked to the short insertion **db1** but not the longer **Db1** segment. See **Extended Data Fig. 7c** for the opposite signatures of substitutions on the **db1:Ca** segment relative to substitutions on the **-Cc1** segment.

(iv) The newly created ends are ligated to other DNA ends (light blue and green), during which deaminated cytosines are converted into substitutions.

In **SI Figure 12B**, we show a 2.5kb insertion from chr8 (chr8:132,804,543-132,807,079) in the HCC1954 genome. This insertion (orange) shares a minus breakpoint (132,807,079) with a larger segment (dark blue). Both the insertion and the large segment show substitutions indicating deamination of the forward strand DNA (downward arrows), but surprisingly, the insertion contains additional deamination that is absent from the large segment. This observation indicates that the insertion and the large segment are derived from separate ssDNA of the same strand, which must have involved replication. A plausible model is shown on the right.

(i) Deamination occurs to ssDNA both near the dsDNA end and internally when the newly synthesized DNA (lagging strand) is resected. (This can also occur when the fork is reversed.)

(ii) Annealing and gap-filling synthesis create a junction and convert deaminated cytosines at the distal end to substitutions on both DNA strands.

(iii) Cleavage of the top strand creates a 5'-ssDNA end.

(iv) A leftward replication fork from the right (red segment) creates the short insertion (orange) from the top strand that contains additional deamination; additional gap-filling synthesis and ligation create the bottom segment that only contains substitutions from the distal deamination. This model is similar to the model shown in **A** except that the bottom strand is ligated but the top strand is cleaved.

In both examples, insertions are created from sequences in the overhang region of a staggered DSB end. The first step is the annealing of the staggered DSB end with a DSB end from an unreplicated DNA segment. After annealing, gap-filling synthesis extends the 3'-end of the partner DSB, but a ssDNA gap remains. Eventually, a replication fork passes through the ssDNA gap, creating two DSB ends, one on the large segment (e.g., **Db2**), the other on the short insertion segment (e.g., **db1**); these two segments can join different translocation partners. Therefore, such insertions are a special outcome of breakage-replication/fusion when one DNA strand is ligated but the other is not.

If the ssDNA overhang undergoes deamination before annealing, then gap-filling synthesis can convert deaminated cytosines into base substitutions on both strands. This occurs to the insertion in **SI Figure 12B**. Additionally, resection can occur to newly generated DNA ends, resulting in deamination on opposite strands; this is reflected in the deamination seen in the **db1** insertion segment and the deamination on the right side of **Bb2** shown in **Extended Data Fig. 7b**.

In bridge clone **a**, we have identified three additional insertions similar to **db1** (1254bp): **bb2** (8052bp), **cc2** (4173 bp), and **j2a_2** (7719bp). These insertions are noticeably longer than most of the other insertions. In the HCC1954 genome, we have also identified similar large insertions, including a 22.6kb insertion shown in **SI Figure 29C**). As insertions generated by the proposed breakage-replication/fusion mechanism is capped by the size of the ssDNA overhang, the observation of large insertions of this type supports both large ssDNA gaps due to hyperrection and highly processive (>1kb) gap-filling DNA synthesis.

4. Rearrangement outcomes of break-induced replication

Break-induced replication (BIR) is commonly associated with the repair of unpaired DNA ends (i.e., without a reciprocal end). For reviews of BIR, see Anand et al. (3), Kockler et al. (13), Verma and Greenberg (37). Here we only discuss the rearrangement outcomes of BIR and how they relate to genomic observations from experimentally generated bridge clones.

SI Figure 13: Breakpoint/rearrangement outcomes of break-induced replication

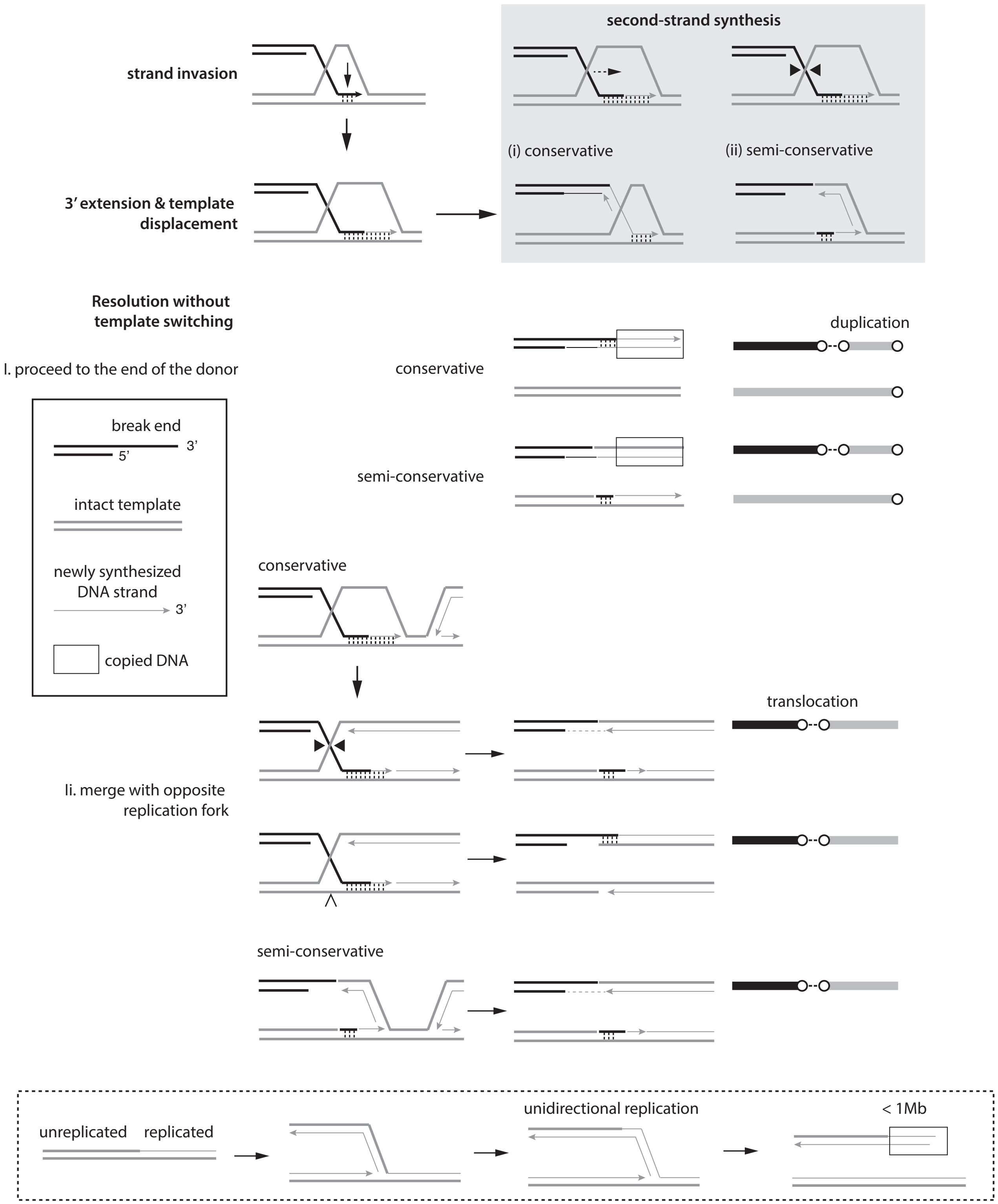

The first step of BIR is strand invasion. This step is mediated by homology-dependent pairing of a break end (black) and an intact donor template (gray). Strand invasion in BIR is similar to homologous recombination (HR), both requiring significant homology between the break end and the donor. By contrast, microhomology-mediated BIR (MMBIR) is thought to require only limited homology (11). We note that homology-dependent strand invasion/annealing occurs in both HR and SDSA (synthesis-dependent strand annealing), whereas microhomology may be used in both non-homologous and alternative end joining (26). Therefore, junction homology is not a definitive signature of BIR or MMBIR.

After strand invasion, the 3'-end (arrowhead) is extended by DNA polymerases (polδ or its subunit *POLD3*). But how lagging-strand synthesis ("second strand synthesis") is carried out is largely unknown. Studies in yeast suggested BIR synthesis to be *conservative* (9, 27); however, it is unknown if the same is true for mammalian BIR or MMBIR. Moreover, it is unclear whether lagging-strand BIR requires some or all of the enzymes involved in normal lagging-strand synthesis, including polα, polδ, and ligase I. See Discussion in Donnianni et al. (10). Both forms of second-strand synthesis (conservative and semiconservative, shaded box) are shown here.

The original model of BIR suggests that the BIR fork can progress to the end of the donor chromosome. However, the rate of BIR synthesis is much lower than normal DNA synthesis. A recent study by Liu et al. (16) suggested the rate of BIR synthesis to be 0.5kb per minute. This is much lower than normal DNA synthesis (mean rate: 1-2 kb/min; maximum rate: 4 kb/min) reported by Baris et al. (4). Even at a rate of 1 kb/min, it will take 1000 minutes, or 16.7 hours, to synthesize 1Mb of DNA. This cannot solely account for the large duplications (>10Mb) observed at broken chromosome ends in our experimentally generated clones. Moreover, Liu et al. (16) suggested that BIR progression is often interrupted by roadblocks such as transcription units. Based on these findings, we consider it *highly unlikely* that BIR synthesis (including MMBIR) can produce large segmental duplications (>1Mb) as observed in human genomes.

If BIR synthesis is not terminated at the end of the donor chromosome, it can lead to three possible outcomes.

The first is when the invading strand is displaced from the donor (template-switching), which creates an insertion outcome. This is the most commonly invoked outcome of MMBIR; however, this outcome does not result in a stable translocation outcome as the DNA end remains unresolved.

The second is when the BIR fork (black) migrates to the end of a donor template (gray) whose size is within the processivity of a BIR fork ($\lesssim$100kb). If the invasion occurs in a subtelomeric region or within the telomere, this process can cap the original DNA end with a de novo telomere (8). If the donor is a DNA fragment, this process will duplicate the donor template but the original DNA end remains unresolved.

The third is when the BIR fork merges with an opposite replication fork. See Smith et al. (30) and Costantino et al. (7). This scenario is only possible when the broken DNA end invades an unreplicated donor. It will generate a translocation between the original DNA end (black) and a sister fragment of the donor chromosome. Notably, when a replication fork merges with a BIR fork, it will create a junction between unreplicated DNA (two template strands) and replicated DNA (one template and one daughter strand); once the replisome from the unreplicated DNA migrates through this junction, it can lead to over-replication (bottom dashed box) that is similar to over-replication as shown in **Extended Data Figure 8**.

Based on the predicted rearrangement outcomes described above, we draw the following inferences about the translocations and rearrangements of the broken ends of chromosome 4 observed in the experimentally generated bridge clones. The translocation outcome in **SI Figure 32D**, **a1:chr4A** is consistent with a MM/BIR invasion, which creates short insertions (~10bp) at the site of invasion due to template switching, followed by merging of the MM/BIR fork with an opposite replication fork. The other outcomes in **SI Figure 32C** and **32D** may arise from a similar process but without template switching, or by direct ligation of the broken ends of chr4 to DNA ends generated by secondary events.

5. A proposed mechanism of copy-number gain and amplification from micronucleation

In our prior studies, we have shown that chromosomes in micronuclei (MN) can both undergo partial replication (replicated DNA shown in red) and acquire DNA breakage (**SI Figure 14**). When the damaged, partially replicated MN chromosome is partitioned between two daughter cells, the fragments retained by each daughter should be largely mutually exclusive, but some replicated fragments may be retained by both daughters (**SI Figure 14**, uneven segregation into both daughters). In our original study (40), we estimated that in the example (MN4) where chr3 was distributed to both daughters, 3% of the chr3 segments were retained by both daughters.

SI Figure 14: Segregation of DNA fragments of a damaged chromosome from a micronucleus

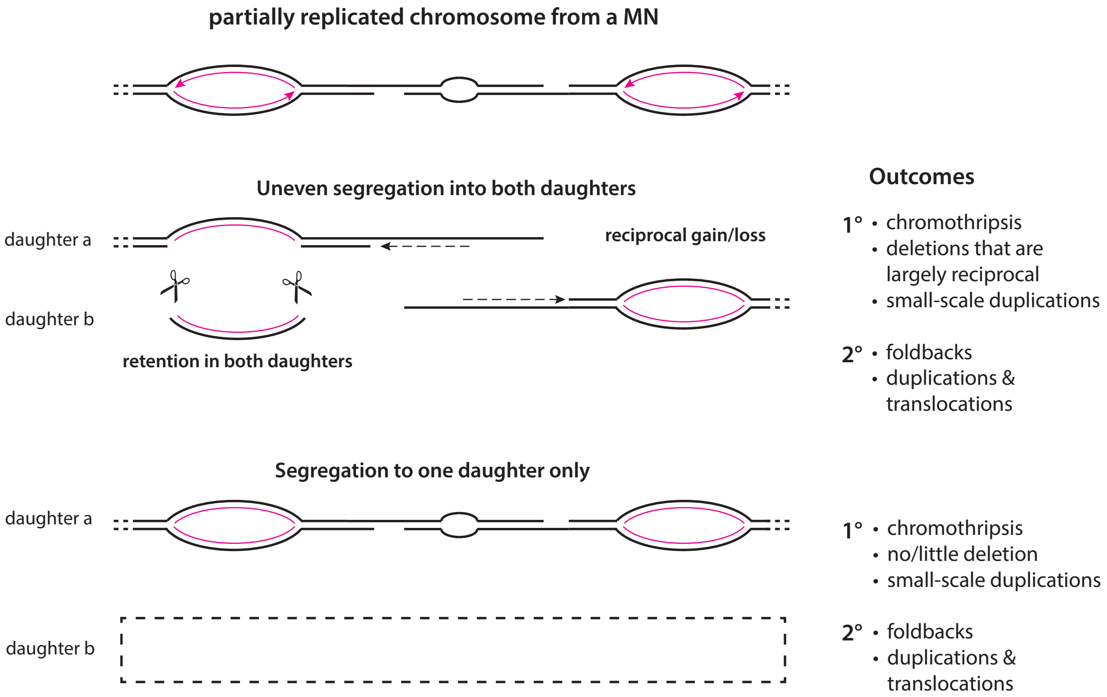

SI Figure 15: DNA segments from conventional replication of a partially replicated chromosome from a micronucleus

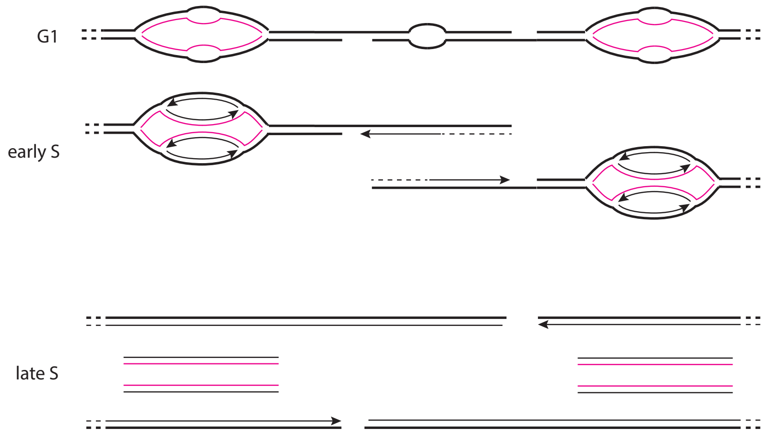

After the MN chromosome is reincorporated into the primary nucleus, a new round of replication can create dsDNA fragments from ssDNA strands on the partially replicated chromosome (**SI Figure 15**). Fusions between the newly generated DNA ends can create both foldback junctions and long-range rearrangement junctions. As the MN chromosome can have up to four DNA strands at a given locus, after one replication/fusion cycle, there can be up to four copies at loci that have undergone partial replication. Segregation of these DNA copies can result in five segmental copy-number states (0-4), not considering the rare instance of re-replication as shown in **Extended Data Figure 8**. Therefore, even if the MN chromosome is taken by one daughter,

breakage-replication/fusion will create copy-number gains and copy-number variation in its progeny. This process can explain the pattern of complex segmental copy-number gains with regional amplification as shown in **Extended Data Fig. 1b**. Notably, there was no segmental deletions of chrX (such deletions would be lethal since there is only one chrX in male cells), indicating that the ancestral chrX must have been largely retained by one daughter.

If the MN chromosome is again partitioned into a newly formed micronucleus (persistent MN), the previously replicated region may undergo another round of partial replication (**SI Figure 16**). The eventual reincorporation of this chromatid into a primary nucleus can convert all the partially replicated DNA into amplified DNA segments. This mechanism is conceptually similar to the amplification of *Drosophila* amplicons in follicle cells (2) except that re-replication takes place over multiple cell cycles.

SI Figure 16: A proposed model for generating amplified DNA with an onion-skin structure from a chromosome having undergone multiple rounds of partial replication in micronuclei.

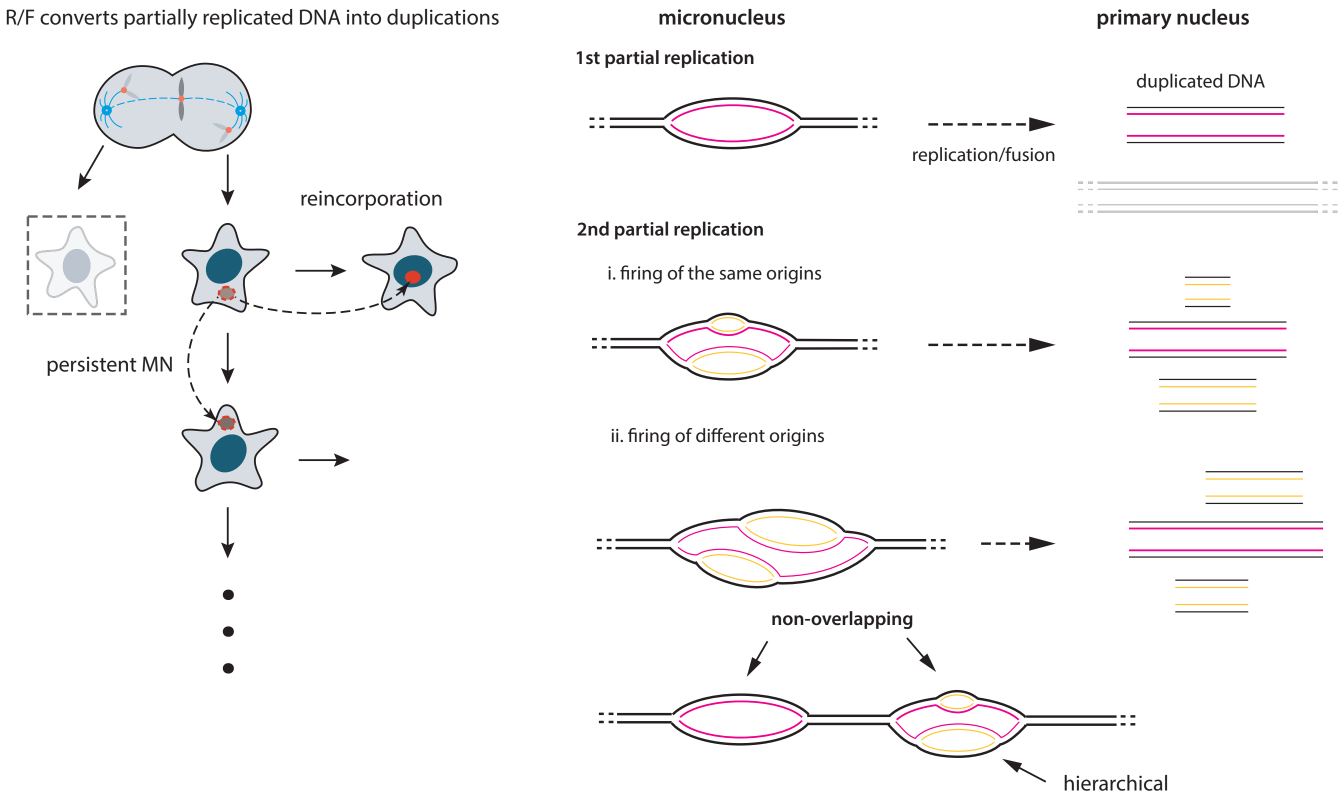

Amplified DNA generated from a chromosome that has undergone multiple rounds of partial replication has the following features. First, partial replication will create discontinuous replicons (partially replicated DNA from different origins); therefore, duplicated DNA derived from these replicons should be mutually exclusive. Second, for replicons generated by replisomes initiated from the same replication origin (fired multiple times in *different* rounds of partial replication), they should form a nested hierarchy; for replicons generated by replisomes initiated from nearby replication origins on different sister DNA copies, they can have partial overlap.

This mechanism provides a compelling explanation for complex amplifications on chr5, chr8, and chr21 in the HCC1954 genome with the following sequence features. First, the concentration of breakpoints in these regions indicates that these chromosomes were sequestered from the rest of the genome (i.e., a micronucleus). Second, many junctions between amplified DNA contain chains of insertions *derived from the same amplified regions*. Finally, the amplified copy-number states suggest multiple rounds of duplications, but the amplified DNA segments are largely non-overlapping (**SI Figure 25 and 26**). The hierarchical pattern of amplified DNA is consistent with predictions of the model shown in **SI Figure 16**.

A specific prediction of the model of "onion-skin" amplification from persistent MN is that amplification should preferentially occur to sequences in *early*-replicating regions. This prediction can be tested in cancer genomes.

Here we present additional genomic observations supporting the Breakage-Replication/Fusion model.

- Sec. 6 Footprints of breakage-replication/fusion in L1 clones contains additional examples of adjacent parallel breakpoints (including foldbacks) and tiling insertions related to **Figure 1d**.
- Sec. 7 Footprints of breakage-replication/fusion in focal amplifications in the HCC1954 genome contains additional examples of adjacent parallel breakpoints and insertions related to **Figures 1-2,5,6** and **Extended Data Figures 2,9**.
- Sec. 8 Translocations and rearrangements of the broken ends of chromosome 4 in bridge clones summarizes translocations and rearrangements of the broken ends of chr4 in the subclones of bridge clone **a** and other primary bridge clones generated in Umbreit et al. (36).
- Sec. 9 Footprints of breakage-replication/fusion in a post-crisis RPE-1 clone and a bridge RPE-1 clone contains examples of adjacent parallel breakpoints and tiling insertions identified in a post-crisis clone from Maciejowski et al. (18) and a bridge clone from Umbreit et al. (36).
- **Sections 10, 11**, and **12** are all about bridge clone **a** and are related to **Figures 3-4** and **Extended Data Figures 4-8**.
- Sec. 13 Adjacent overlapping breakpoints in chromothripsis in the K-562 genome contains examples of adjacent overlapping breakpoints in the K-562 cells related to **Extended Data Figure 8**.
- Sec. 14 Insertions in single cells after bridge resolution contains examples of tiling insertions related to **Figure 5** and **Extended Data Figure 9**.

6. Footprints of breakage-replication/fusion in L1 clones

In this section, we present additional examples of rearrangements showing signatures of breakage-replication/fusion in clones derived from single cells with transient L1 expression. We first show three instances of adjacent parallel breakpoints that are directly related to L1 ORF2p-induced double-strand breakage and reverse transcription in **SI Figure** 17.

SI Figure 17: Adjacent parallel breakpoints at L1 ORF2p-induced DSB sites

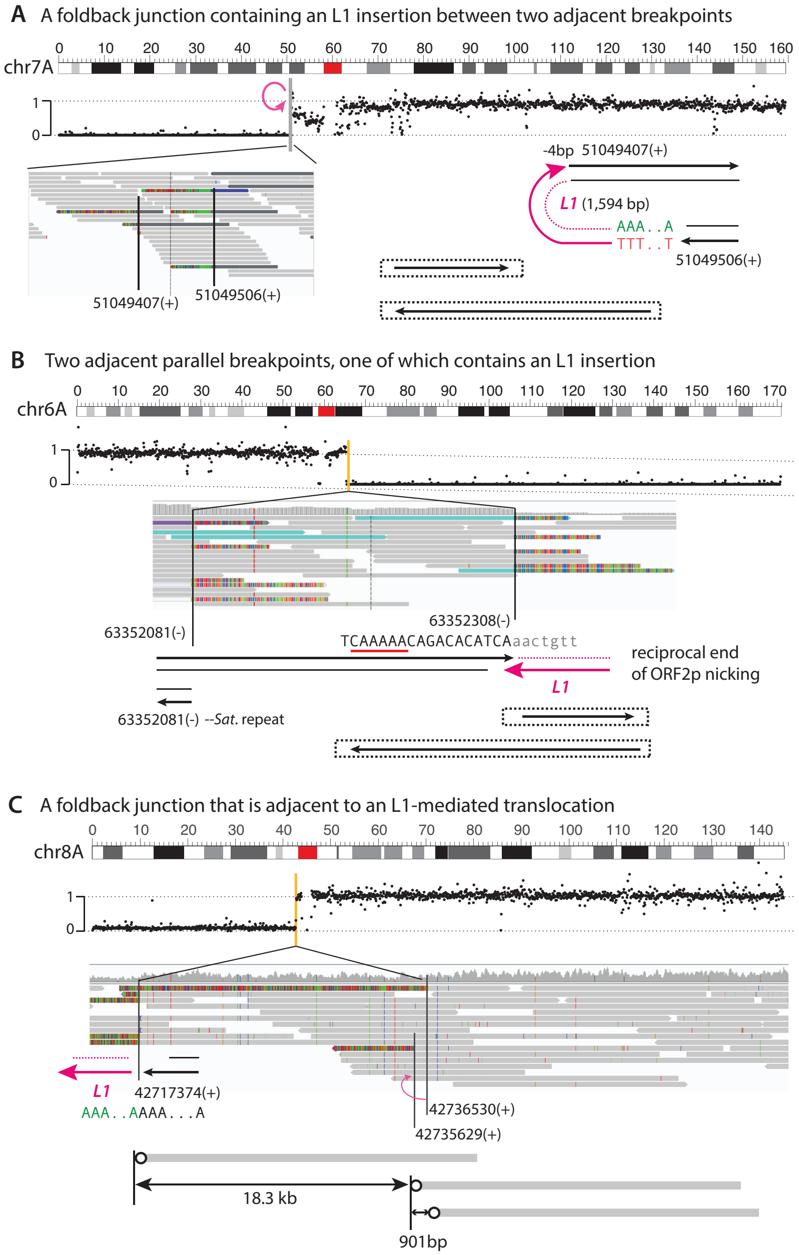

SI Figure 17A shows a foldback junction containing a truncated L1 (1,594 bp) between two adjacent parallel breakpoints (49bp distance). The junction is resolved by long reads. The solid magenta line represents the first cDNA strand (arrow points to the 3') from reverse transcription carried out by ORF2p, the dotted line represents the second strand of the insertion. We infer the two breakpoints to have been generated by ORF2p nicking opposite DNA strands, first at 51,049,506, from which reverse transcription starts, then at 51,049,407 on the complementary DNA strand. The two ssDNA ends are likely tethered by annealing (4bp homology, '-4bp') between the insertion and the leftmost breakpoint at 51,049,407, but DNA replication is required to form the foldback junction. The reciprocal DNA ends (lost from the clone as indicated by complete terminal deletion) are shown in dashed boxes.

SI Figure 17B shows two adjacent parallel breakpoints (227bp distance) that form separate translocation junctions, one of which containing an L1 insertion. The breakpoint at 63,352,308 joins the 3'-end of a reverse-transcribed L1; the breakpoint at 63,352,081 joins a satellite repeat sequence. The junctions are not fully resolved as long reads are unavailable. We infer that both breakpoints are derived from a staggered DSB generated by ORF2p based on presence of an ORF2p EN cutting site near the distal breakpoint (`Tc|AAAA`).

SI Figure 17C shows a foldback junction next to a translocation with a 731bp L1 insertion. The two breakpoints of the foldback junction [42735629(+) and 42736530(+), 901bp distance] are 18kb downstream from the breakpoint [42717374(+)] with L1 insertion. We infer that all three breakpoints are derived from one ancestral DSB end created by L1 ORF2p: The 3'-end on the reverse strand undergoes reverse transcription to create the insertion junction; the 5'-end on the forward strand undergoes two rounds of replication and one fusion to create the foldback junction.

In addition to rearrangement junctions with L1 insertions, we have also identified many examples of rearrangement junctions that show signatures of breakage-replication/fusion but do not display direct footprints of L1 retrotransposition. Given that almost no segmental rearrangement was observed in control clones without L1 expression, we expect many of these events arise either directly from, or downstream of ORF2p-induced double-strand breaks. As described in Mendez-Dorantes et al. (20), L1 ORF2p creates two DNA ends but usually only one end (with poly-T sequences) is extended by ORF2p-mediated reverse transcription; therefore, translocations/rearrangements from the reciprocal end do not have L1-mediated insertions. Moreover, L1-mediated translocations can generate unstable chromosomes that undergo secondary alterations, during which the footprints of ORF2p-mediated reverse transcription are deleted.

SI Figure 18 shows two nested deletions on chr3 with junctions containing inverted genomic DNA sequences. The presence of poly-T sequences (potential ORF2p EN target sites) near breakpoints at **98,025,382** and **98,025,375** supports an origin of both breakpoints from an ancestral DSB generated by ORF2p. The insertions are derived from sequences next to two deletion boundaries: insertion **98,025,375**-98,025,647 next to **98,025,382(-)**, insertion 98032161-**98032634** next to **98,032,635(+)**. The insertions are possibly derived from the 3' overhangs of staggered DSB ends.

SI Figure 18: Two nested deletions with local sequence inversion in an L1 clone

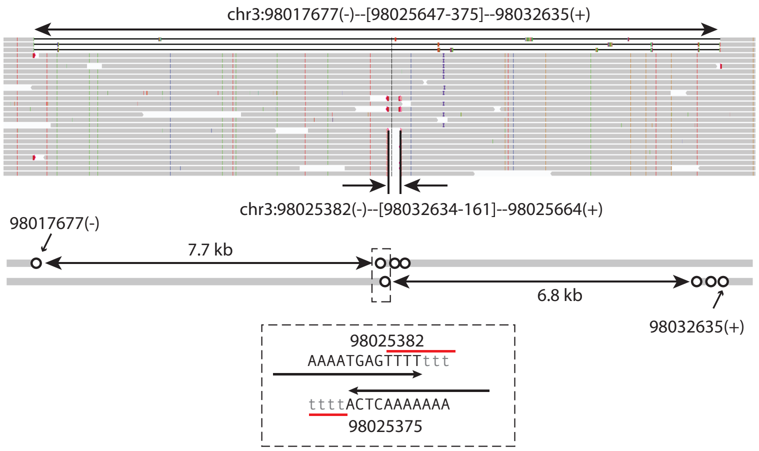

SI Figure 19 shows an example of multiple translocations involving chr2 (panel **A**), chrX (panel **C**), and chr5 (panel **E**). Based on the translocation junctions, we infer two translocated chromosomes, der(2)t(2;X) (panel **B**) and dic(5;X) (panel **D**). The inferred dicentric chromosome is supported by copy-number variations on the p-arm of chrX and the q-arm of chr5 that are between the two centromeres. Notably, three out of four translocation breakpoints are linked to foldback junctions: chr2:201,802,139(-)/chr2:201,802,154(-), chrX:1,878,418(+)/1,878,418(+), and chr5:103,246,812(-)/103,248,389(-). On chr2, there are two additional foldback junctions, which indicate secondary replication/fusion cycles initiated by an ancestral DSB. On chrX, the breakpoint at chrX:1,869,299(-) is adjacent to five strand-coordinated substitutions, indicating an origin from a 3'-ssDNA end. This breakpoint and the opposite breakpoints of the foldback junction [both at chrX:1,878,418(-)] suggest an ancestral DSB followed by breakage-replication/fusion as shown in **Figure 1b**. The ancestral DSB ends may have been generated by L1 based on the presence of an ORF2p EN target site (TTTT|A) near 1869299(-). The inserted sequence is likely cleaved before completion of second-strand synthesis.

SI Figure 19: Multi-chromosomal translocations with foldback junctions

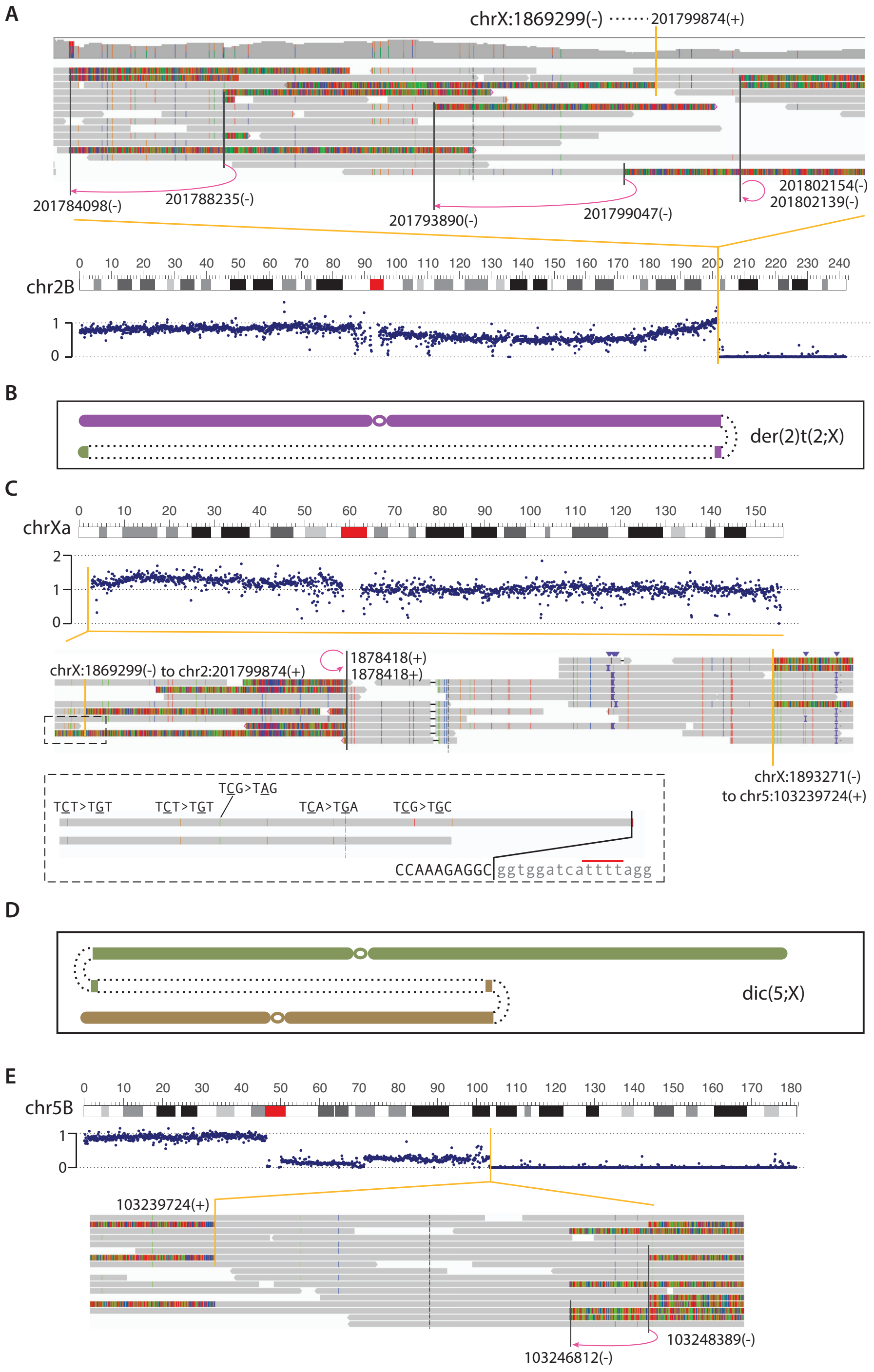

SI Figure 20 shows an example of three foldbacks in the pericentric region of chr1 (115.4Mb-cen). Based on the DNA copy number (blue dots for the altered 1B homolog) in panel **A**, we infer that the region of copy-number gain is not adjacent to a broken chromosome end. Moreover, the adjacency between foldback breakpoints (panel **B** and **C**) indicates that these breakpoints originate from reciprocal DNA ends that undergo breakage-replication fusion: The two breakpoints 116,074,510(+) and 116,074,782(+) are derived from a DNA end that is reciprocal to the ancestral DNA end of the breakpoint 116,074,348(-); the presence of breakpoint 116,078,420(-) further indicates that the breakpoint at 116,074,348(-) to have been derived from a ssDNA end. Therefore, these breakpoints are generated by two rounds of breakage-replication/fusion. Finally, based on the copy number of breakpoint 115,738,331(+) determined from the copy number of its partner breakpoint on chrX (≈0.8), we infer that

the foldback junctions at 115,373,458(+)/115,372,059(+) and at 116,074,348(-)/116,078,420(-) are both duplicated at least once (shown in panel **E**). This example supports the model of DNA amplification shown in **Figure 2b** of the main text.

SI Figure 20: Nested foldbacks at an internal locus

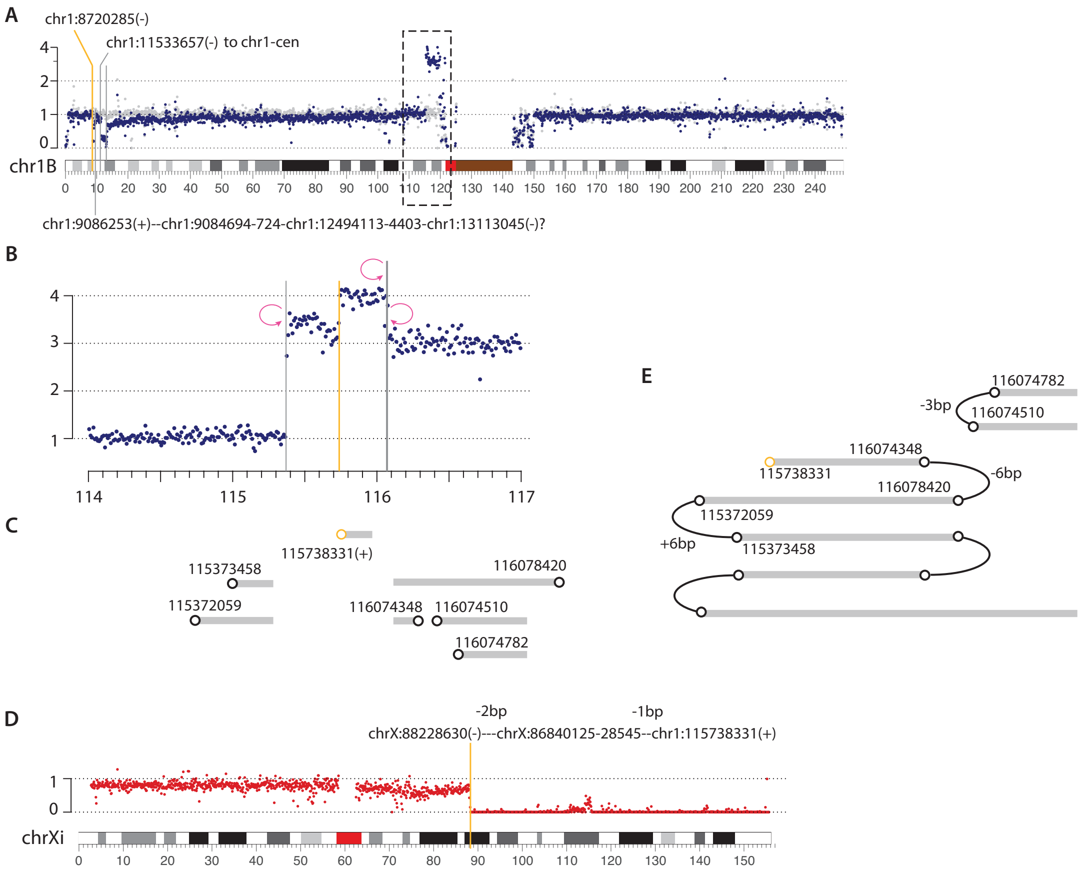

SI Figure 21 shows an example of chromothripsis after an ancestral DSB generated by L1 (with L1 insertion) that causes deletion of the terminal segment from 103Mb on chr7. We identified four pairs of adjacent parallel breakpoints near 66.94Mb and flanking duplications between 82.48 and 83.36Mb (green boxes). The inferred structure of duplicated DNA segments is shown above the copy-number plot.

SI Figure 21: Adjacent parallel breakpoints in L1-induced chromothripsis

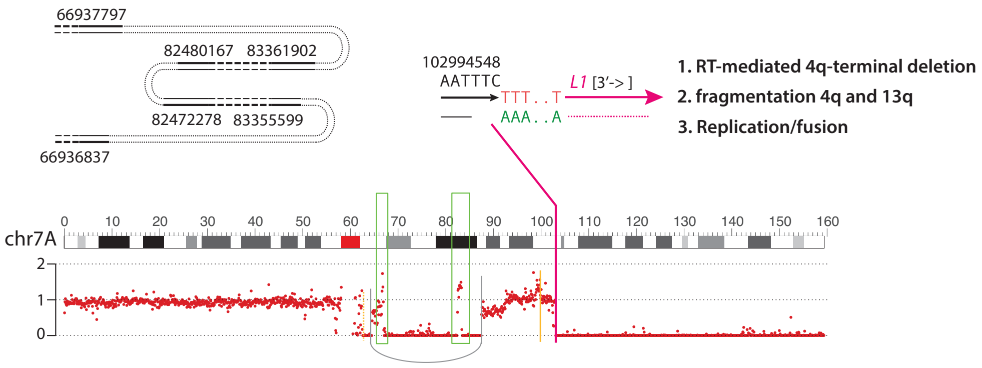

Finally, **SI Figure 22** shows an example of chromothripsis involving both the p-terminal region of chr5B and the p-arm of chr12A. Black arcs represent intra-chromosomal junctions and magenta lines represent inter-chromosomal breakpoints. A representative example of tiling insertions near chr12:7.7Mb is shown below.

SI Figure 22: Tiling insertions in L1-induced chromothripsis

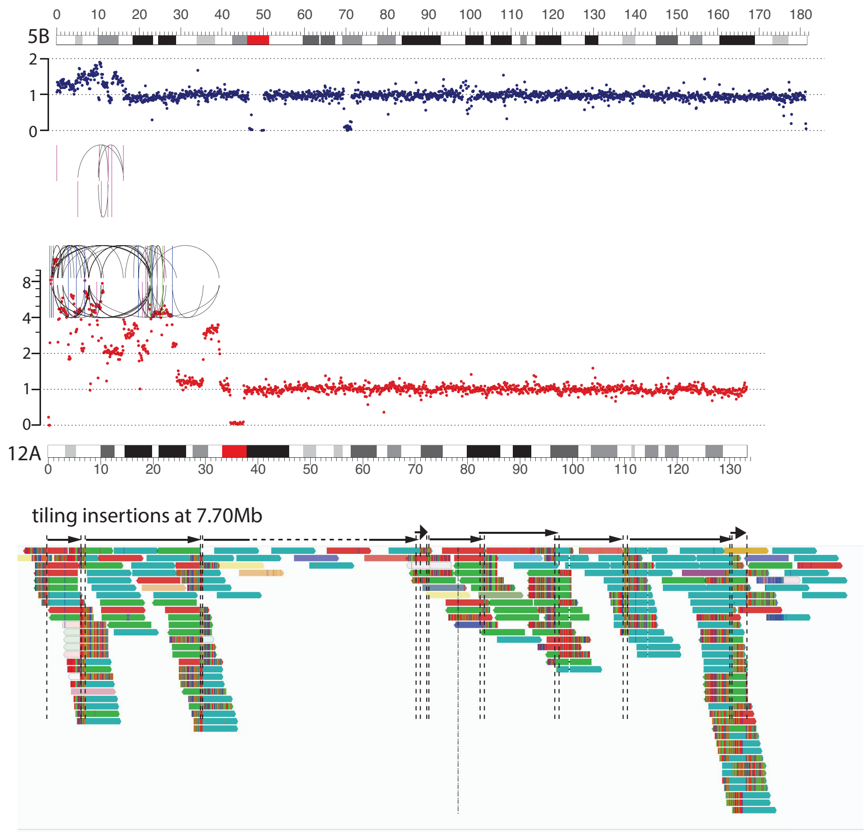

7. Footprints of breakage-replication/fusion in focal amplifications in the HCC1954 genome

SI Figure 23: Haplotype-specific DNA copy number and rearrangements in the HCC1954 genome.

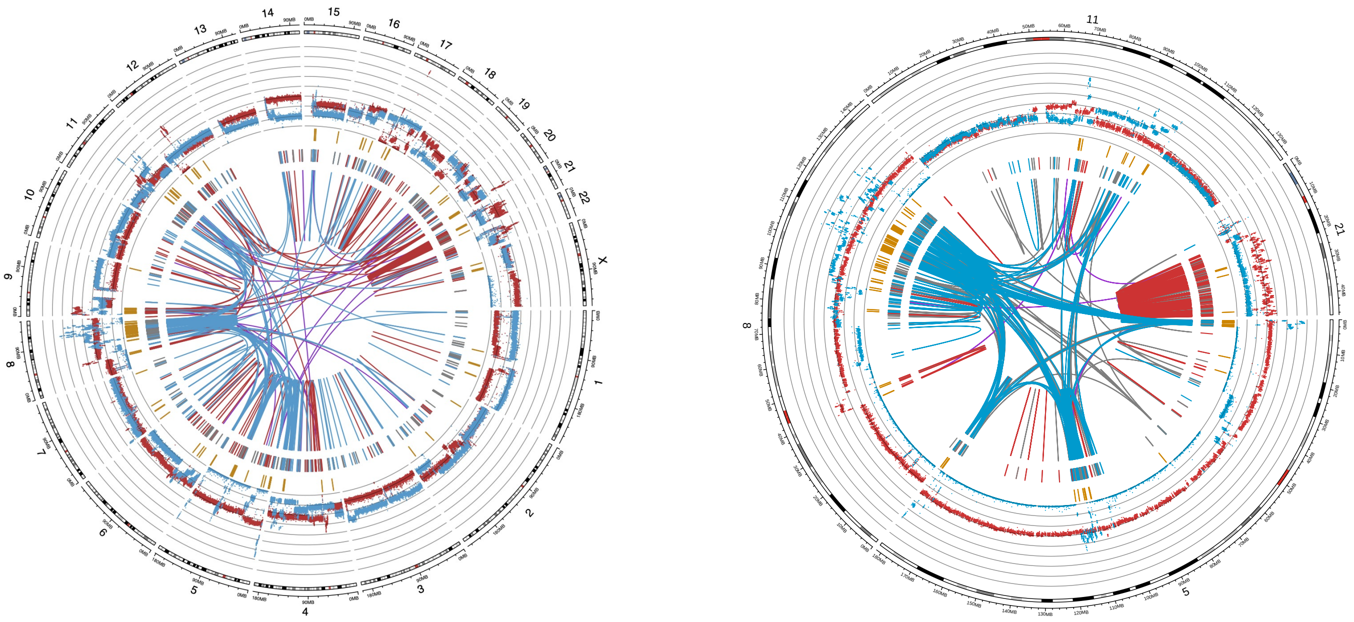

The breast cancer cell line HCC1954 is one of the first cancer genomes analyzed by whole-genome sequencing (32). The HCC1954 genome displays extraordinary complexity of amplified DNA from chr5, chr8, and chr21 as shown in **SI Figure 23**. In the two CIRCOS plots (left for the entire genome, right for chr5, chr8, chr11, and chr21), haplotype-specific DNA copy number data are shown as red and cyan dots on the outer ring, rearrangements are shown as inner arcs and colored based on the parental chromosome of the two breakpoints represented by red or cyan vertical bars. The orange bars next to the copy-number traces represent foldback junctions. The haplotype phase of parental chromosomes is determined from linked-reads and Hi-C data using `mLinker` as described previously (6, 35). Linked-reads data for the HCC1954 and its matching germline reference HCC1954BL were generated by 10x Genomics. The linked reads were also used to determine the haplotypes of rearrangement breakpoints. Hi-C data for the HCC1954 and HCC1954BL were generated by us and available from the NCBI short read archive under BioProject PRJNA1079784. For analyses presented in this section, we have also used PacBio and nanopore sequencing data that are available

SI Figure 24:. Haplotype-specific copy number of chr17 in HCC1954.

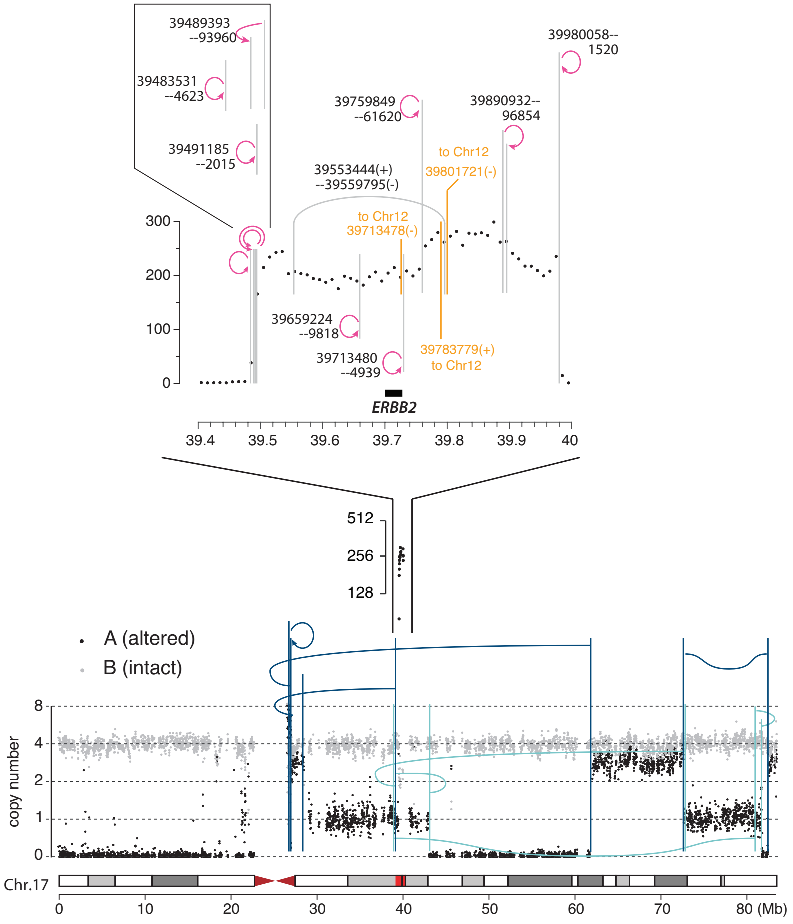

under BioProject PRJNA1086849.

In **Figure 2** and **Extended Data Fig. 2**, we have shown focally amplified regions on chr8p, chr12p, chr17q and chr20q with nested foldbacks. In further support of the model of linear ecDNA amplification of the *ERBB2* amplicon as shown in **Fig. 2b**, **SI Figure 24** shows that this amplicon does not overlap with other segments on 17q, indicating an origin from chr17q fragmentation. For further analysis of 17q segments, see Brunette et al. (6).

In this section, we focus on amplifications on chr5, chr8q, and chr21q with both foldbacks and complex long-range rearrangements. Based on rearrangement junctions, chr21 breakpoints predominantly form intra-chromosomal junctions; there are many inter-chromosomal rearrangement junctions both between chr5 and chr8, and connecting chr5 and chr8 segments to other chromosomes. Although it is impossible to determine the complete structure of amplified DNA, we are able to infer the structure of amplified segments based on their *cis* Hi-C contacts with segments from other chromosomes that are joined together in the rearranged chromosome. These results are shown in **SI Figure 25 and 26**.

For chr21, amplified DNA segments are joined to segments from chr2, chr8 (both A and B homologs), chr9, chr12, chr14, chr20, and chr22. We show representative Hi-C contacts between chr21 and regions from chr2 and chr8 (both homologs) in **SI Figure 25A**. The first set of segments join segments join chr8B:39-43Mb; the second set of segments join chr2A:199-205Mb and chr8A:80-85Mb; a third set of segments join chr8A:105-121Mb. The boundaries of these segments are outlined.

SI Figure 25: Amplified chromosomal segments from chr21 and chr5 inferred from inter-chromosomal Hi-C contact maps

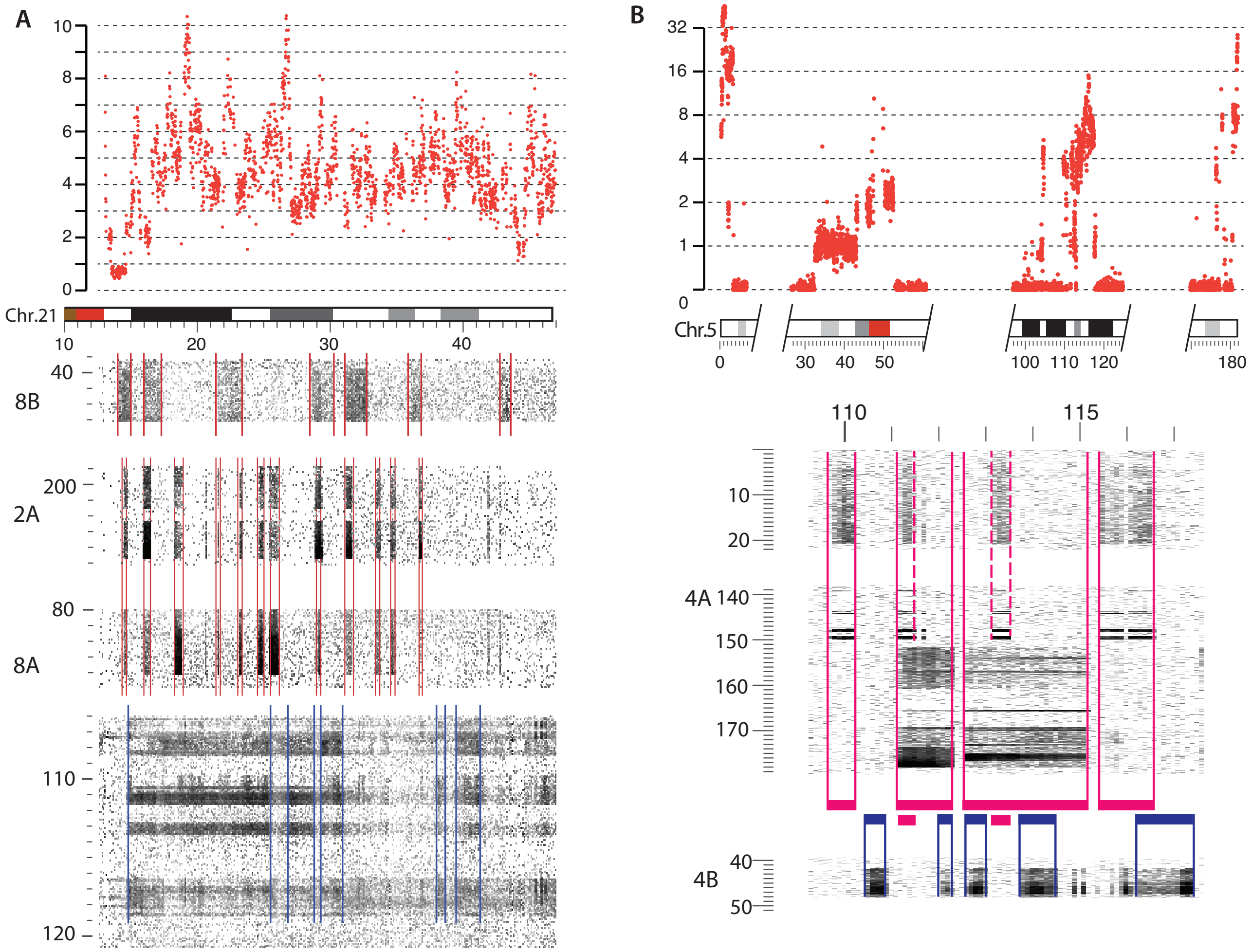

As shown in **SI Figure 6**, multi-copy DNA gains or amplifications can have many different segmental configurations reflecting different pairing between breakpoints. However, the segments inferred from the Hi-C maps show a largely *inclusive* pattern, i.e., smaller segments are either entirely contained in a larger segment, or are mutually exclusive; segments with large partial overlap (intersecting) are rare. The same pattern is observed for amplifications from chr5 (**SI Figure 25B**) and chr8 (**SI Figure 26**).

SI Figure 26: Amplified chr8 segments inferred from inter-chromosomal Hi-C contact maps

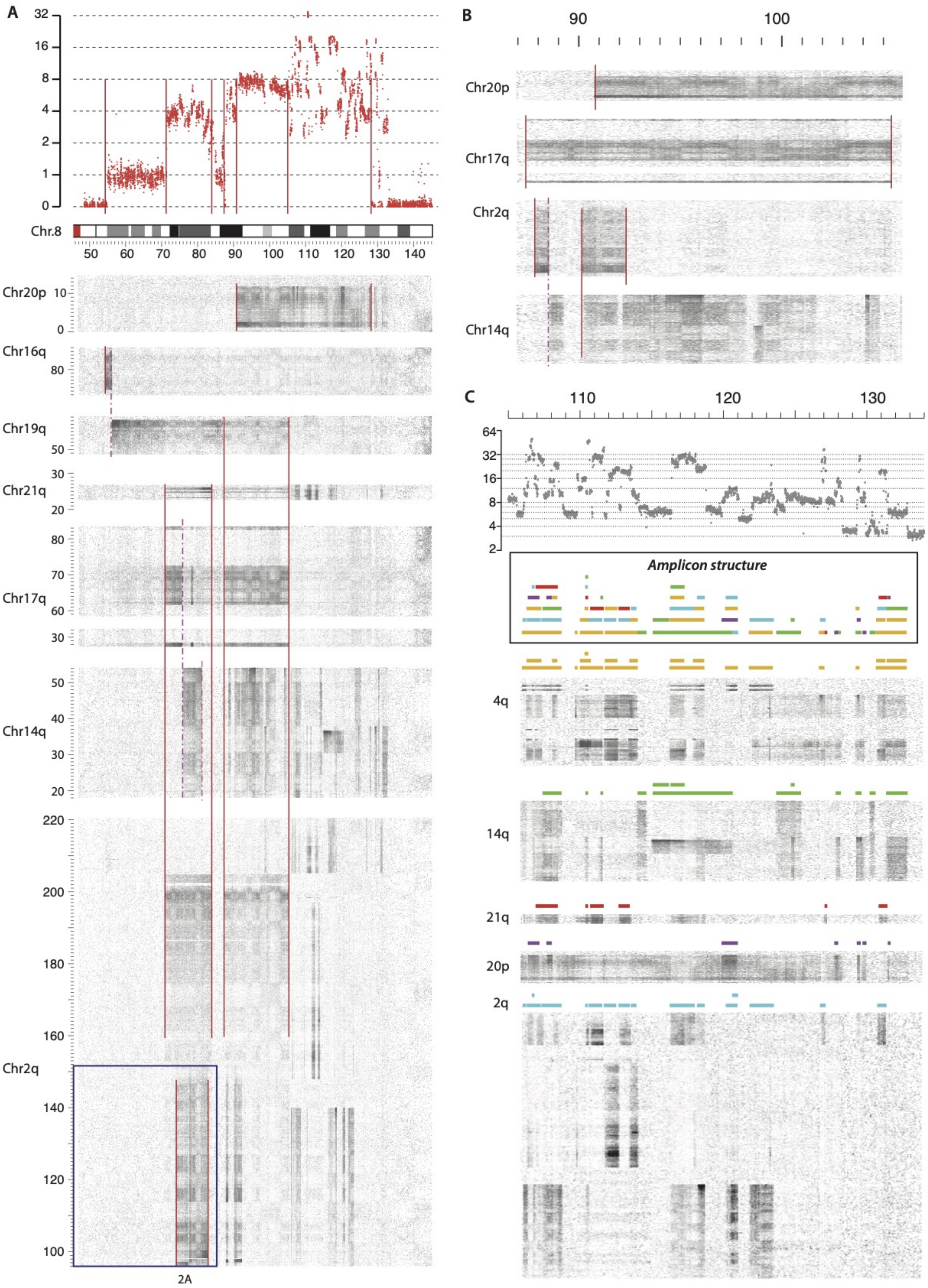

Amplified DNA from chr5 is derived from three regions: 0-5Mb, 109-117Mb, and 179-182Mb (**SI Figure 25B**, top; copy number data above two are log2 transformed). There are *cis* Hi-C contacts between the largest region of amplification (109-117Mb) with chr2, chr4, chr8, chr11, chr20, chr22 and chrX. We infer that there are three set of segments (**SI Figure 25B**, bottom), the first join segments from the p-terminal region of chr4A, the second join segments from the q-terminal region of chr4A, and the third join regions from chr4B:40-50Mb. In total, there are 11 different segments based on the Hi-C contact map; only two segments display partial overlap (i.e., intersecting).

Amplified DNA from chr8q is derived from 70-130Mb and consists of multiple segments that join DNA from multiple chromosomes. **SI Figure 26A** show the Hi-C contact maps between 8q and various chromosomes. Except for chr2 for which both homologs are involved in rearrangements with chr8 segments, segments from the remaining chromosomes are all derived from a single homolog. Amplified chr8 segments are derived from three regions. The first is from 70Mb to 84Mb; no two segments from this region are intersecting. The second is from 87 to 106Mb; again, no two segments from this region are intersecting (**SI Figure 26B**). These two regions are contained in a larger single-copy segment from 54 to 105Mb that is determined from the Hi-C contact map between chr8 and chr19. This larger segment does overlap with another segment (91-129Mb) that is inferred from the Hi-C contact map between chr8 and chr20. In addition, there are many small segments from 106-132Mb that are amplified to very high copy-number states; partial overlap between these segments is also rare (**SI Figure 26C**). (We have highlighted segments resolved by the Hi-C contact maps using red bars.) Together, these data all indicate that partially overlapping segments are very rare.

SI Figure 27: Examples of adjacent parallel breakpoints with reciprocal breakpoints in amplified regions in HCC1954

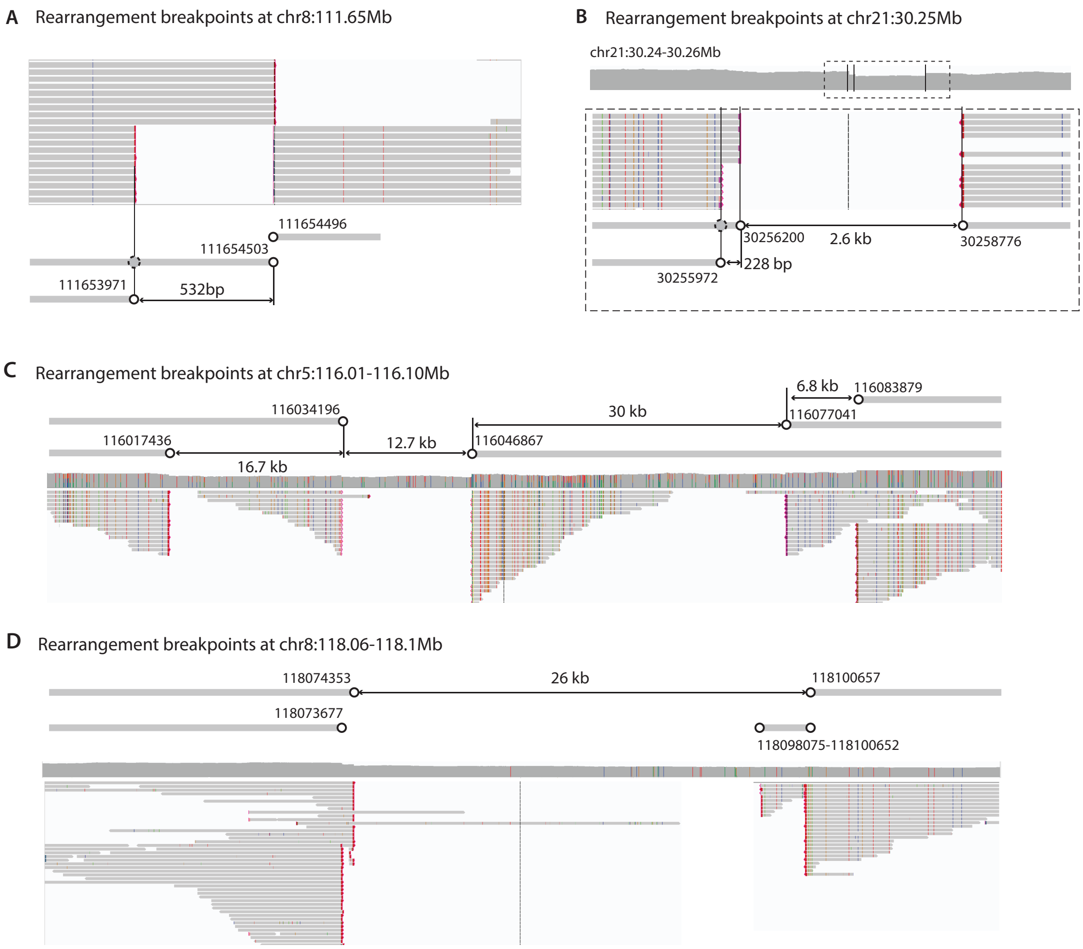

In contrast to amplified segments joined by foldback junctions (chr17q, chr8p, chr12p, and chr20q) as shown in **Figure 2** and **Extended Data Figure 2**, amplifications on chr5, chr8q, and chr21 are joined by both foldback and long-range junctions. We identified 56 pairs of adjacent parallel breakpoints in these regions, a few of which have reciprocal breakpoints (shown in **SI Figure 27**). Notably, 46 pairs form long-range rearrangement junctions and only 10 form foldbacks. By contrast, only 11 adjacent

parallel breakpoints forming long-range instead of foldback rearrangement junctions were identified elsewhere (**Supplementary Table 3**). The prevalence of adjacent parallel breakpoints in chr5, chr8q, and chr21 that form long-range junctions instead of foldbacks indicates that many of amplified DNA segments are generated contemporaneously during breakage-replication/fusion cycles after these chromosomes were fragmented in a single ancestral catastrophe. This is further supported by the observation of short insertions mapped to regions near the breakpoints of large segments.

In **SI Figure 28**, we show insertions (short bars) near breakpoints (open circles) flanking the amplified segments from chr5:116-117Mb. Based on the Hi-C contact map (**SI Figure 25**), we infer this region to be joined to segments from chr4B. Near the left boundary (top IGV snapshot), there are 13 insertions, all non-overlapping; near the right boundary (bottom IGV snapshot), there are three insertions in the offset region between two adjacent breakpoints, and another insertion near the distal breakpoint.

SI Figure 28: Short insertions near breakpoints flanking the amplified segments from chr5:116-117Mb

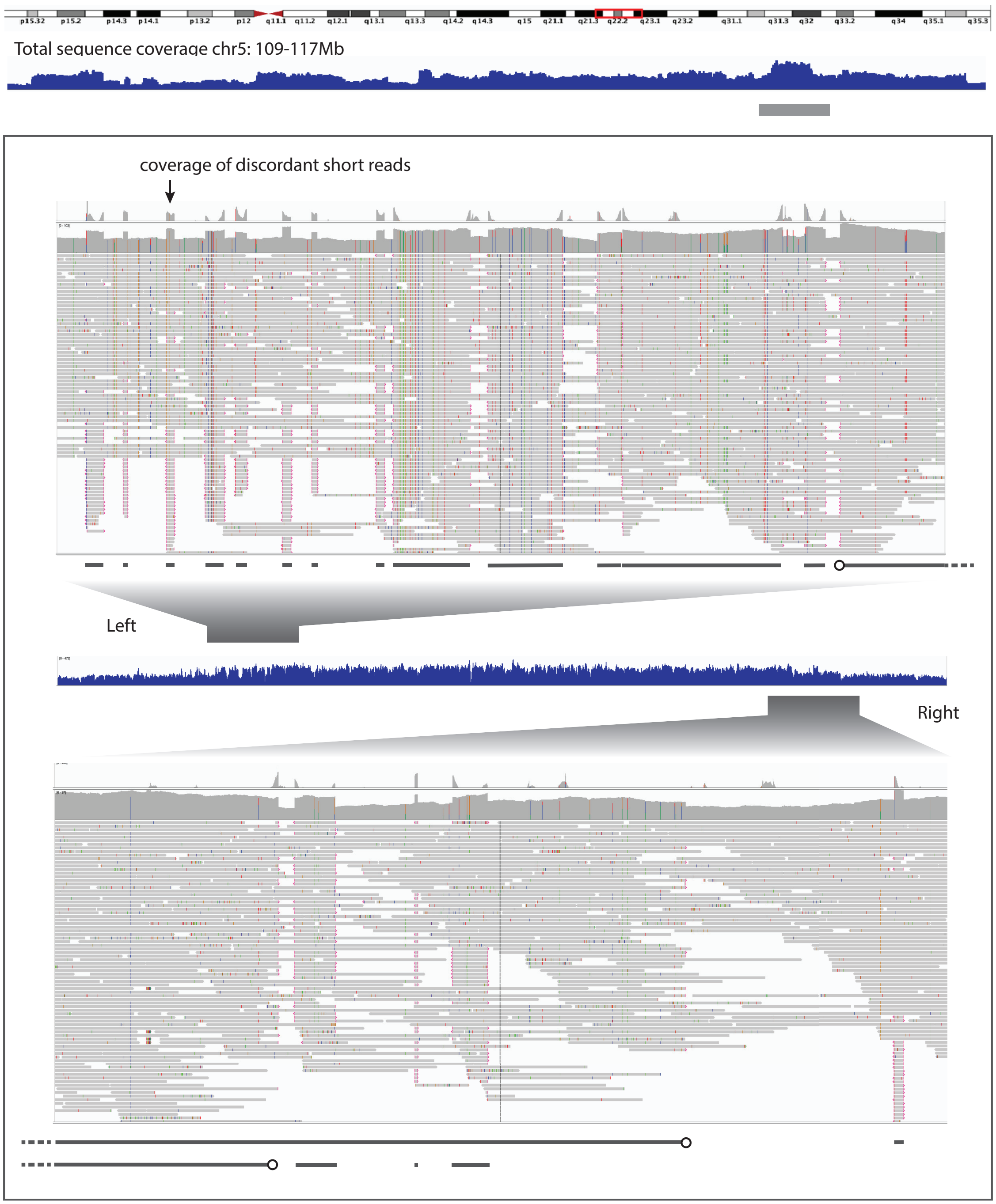

In **SI Figure 29**, we show insertions near breakpoints flanking the amplified segments from chr5:180-181.5Mb. Based on the Hi-C contact map (not shown here), we infer this region to join segments from chr4A:155-179Mb. Near the left boundary (panel **C**, left), there are eight non-overlapping insertions (there sizes annotated as basepairs). The longest one is 22.6kb and shares a breakpoint with the large segment (see **SI Figure 12**). Near the right boundary (panel **C**, right), there are two adjacent breakpoints with different translocation partners; in addition, we also identified three insertions near these breakpoints.

SI Figure 29: Short insertions near breakpoints flanking the amplified segments from chr5:180-181Mb

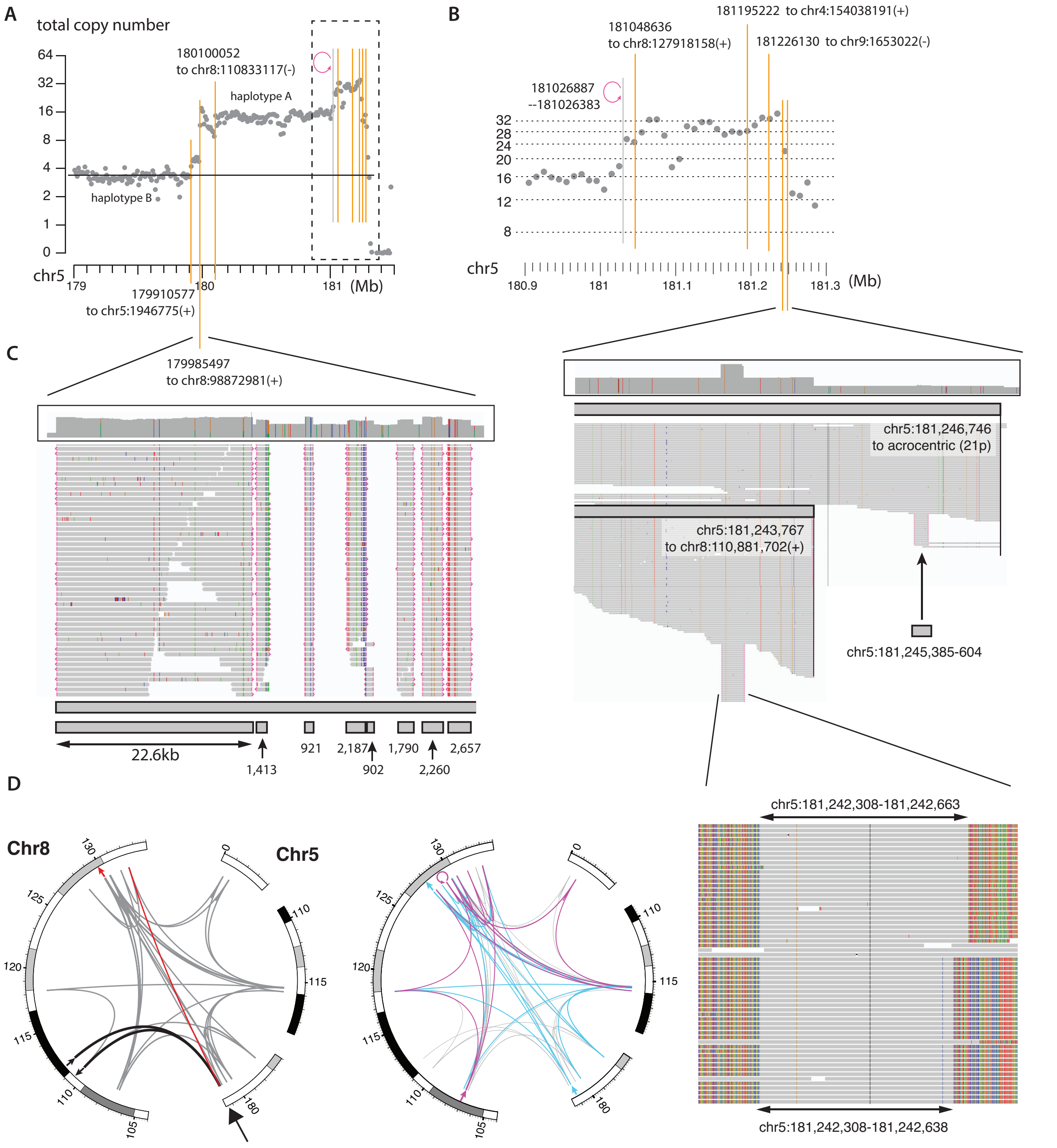

 Cheng-Zhong Zhang, Carlos Mendez-Dorantes, Kathleen H. Burns, David Pellman

Before discussing the special instance of insertions shown in **SI Figure 29D**, we want to first summarize the genomic features of insertions identified in the HCC1954 genome (**Supplementary Table 20**). In total, we identified 372 insertions that are shorter than 20kb (median: 613bp; mean: 2,322bp); only 18 insertions are above 10kb. We identified 52 pairs of adjacent overlapping breakpoints within 25kb (**Supplementary Table 21**). The distance between overlapping breakpoints (median: 6,408 bp; mean: 7,295 bp) is much longer than the size of insertions (median: 613bp; mean: 2,322bp), and 16 out of 52 pairs of adjacent overlapping breakpoints are separated by >10kb. The different distance ranges highlight the different underlying mechanisms for insertions (**Figure 5** and **Sec. 3**) and adjacent overlapping breakpoints (replication bypass, **Extended Data Fig. 8c**)).

The insertions identified in the HCC1954 genome further display the following features. First, many insertions originate from amplified regions from chr5 (103 insertions), chr8 (125 from 8q, 2 from 8p), and chr21 (42). Second, insertions are often mapped to adjacent origins, as shown in **SI Figure 28** and **29**. Finally, many insertions form chains at rearrangement junctions. In **SI Figure 29D**, the first plot (left) shows a junction containing 47 insertions (gray arcs) between chr8:111,694,761 (black arrow) and chr8:129,889,115 (red arrow). See **Supplementary Table 22** for the original sites and the joining order of the insertions in this chain. Two more examples of junctions with multiple insertions (magenta and blue) are shown in **SI Figure 29D**, middle (**Supplementary Table 23** and **24**). These examples highlight the proximal origins of insertions in all three chains that are shown in **SI Figure 28** and **29C**.

The two insertions near chr5:181,243,767 as shown in **SI Figure 29C** are special: They show flush breakpoints on one side and ‘staggered’ breakpoints on the other (**SI Figure 29D**, right). The longer sequence (356bp) is inserted between breakpoints at chr8:111,694,761 and chr8:110,510,787 (black arrows in **SI Figure 29D**, left); the short sequence (331bp) is one of 47 insertions in the longer junction. We refer to such pairs of insertions as ‘nested’ insertions. More examples of nested insertions are shown in **SI Figure 30**.

SI Figure 30: Examples of nested insertions originating from amplified regions of chr5 in HCC1954

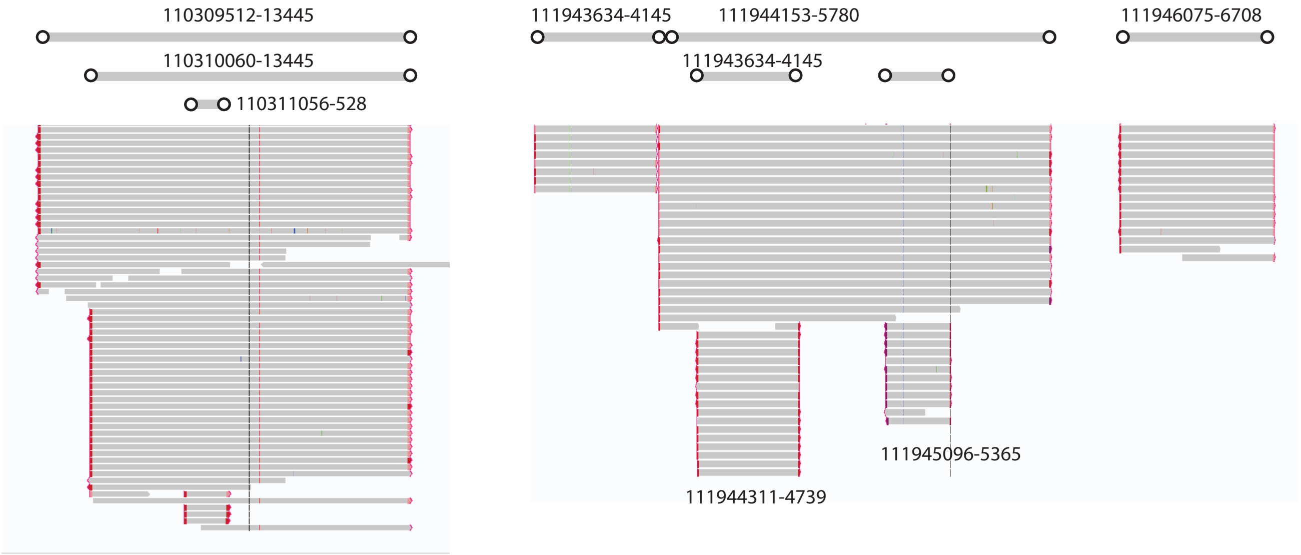

For nested insertions with a flush breakpoint, the flush breakpoint must be generated by ligation before the two DNA strands are replicated, whereas the staggered breakpoints are generated by ligation after replication. The most plausible explanation for nested insertions is that the shorter insertion derived from the longer ssDNA fragment during two replication/fusion cycles. See **SI Figure 12** for a possible mechanism.

A particular interesting example is the insertion chain shown in magenta in **SI Figure 29D**. This insertion chain is added to the breakpoint at chr8:106,992,630(-). However, the last three insertions in the chain, chr8:129,328,018-832, chr8:129,331,231-2,889, and chr8:129,328,841-8,018 result in a foldback junction. Note that the two insertions chr8:129,328,018-832 and chr8:129,328,841-8,018 are nested, share a flush breakpoint chr8:129,328,018, but joined at an inverted orientation in the junction. The formation of a foldback requires DNA replication after the concatenation of the insertions. Therefore, in the ancestral chromosome, the insertions are added to the DNA end at chr8:106,992,360, but not fully ligated; the DNA then undergoes another round of replication/fusion to create a foldback junction with a palindrome of insertions.

SI Figure 31: All instances of overlapping insertions in HCC1954

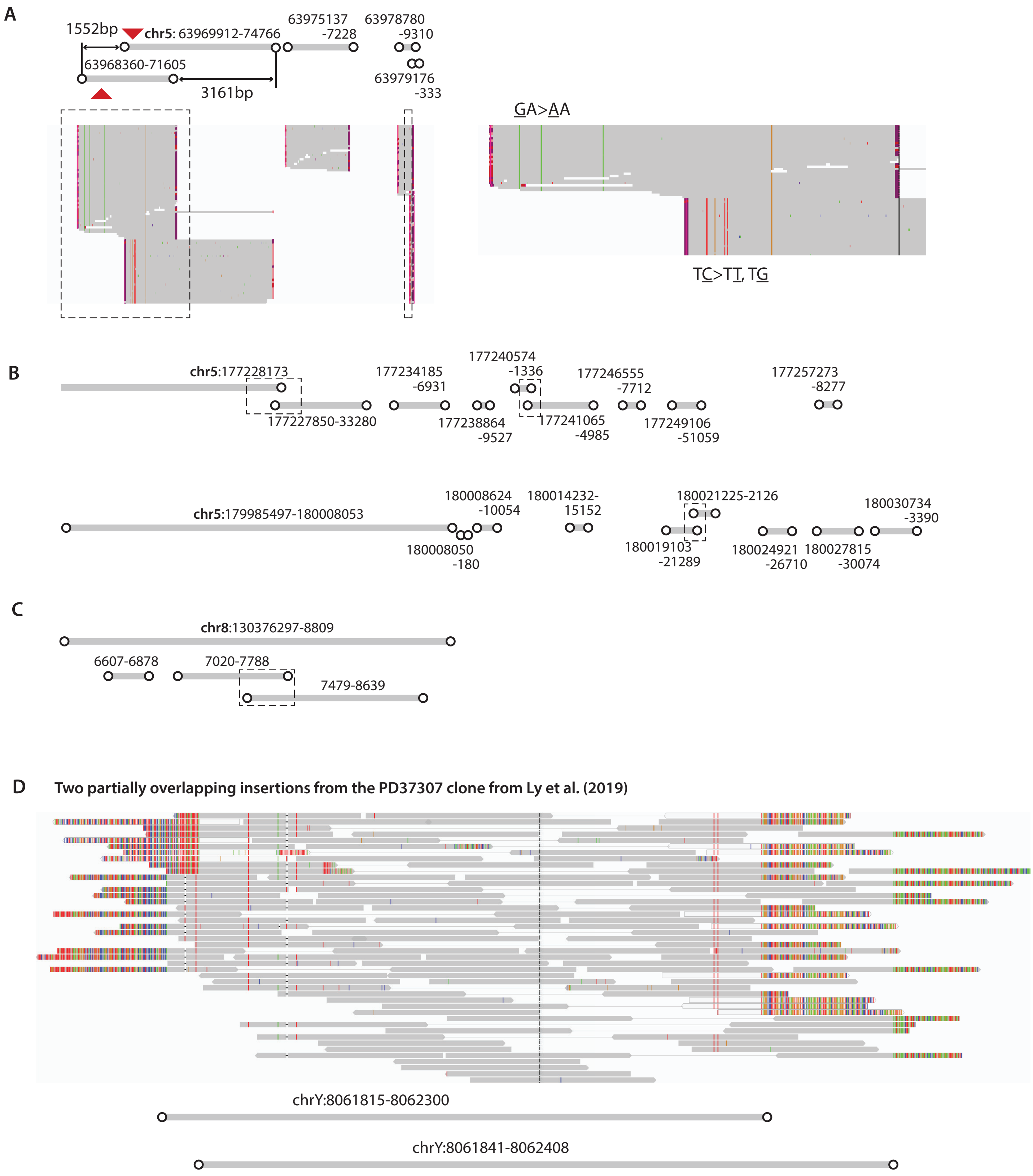

 Cheng-Zhong Zhang, Carlos Mendez-Dorantes, Kathleen H. Burns, David Pellman

Finally, **SI Figure 31A-C** show all six instances of adjacent insertions with partial overlap (dashed boxes). In the first example shown in **SI Figure 31A**, the two insertions show deamination on opposite strands on the left side (zoomed IGV screenshot on the right), providing definitive evidence that they are derived from opposite DNA strands. (By contrast, the nested insertions shown in **SI Figure 12B** display the same deamination signature.) Moreover, the simultaneous deamination of both DNA strands implies that they are not in a duplex; one possibility is that they are unwound by a helicase. In further support of the model, we identified an example of two 'sister' insertions with staggered breakpoints on both sides (**SI Figure 31D**) in the PD37307 clone (**Extended Data Figure 3**). Presumably, these two insertions originate from two complementary strands of a short dsDNA.

In summary, the following genomic observations of complex amplifications and insertions in HCC1954 are consistent with breakage-replication/fusion. First, many amplified segments are bound by adjacent parallel breakpoints, indicating DSB ends on the ancestral DNA fragments. Moreover, most of these breakpoints form long-range junctions instead of foldback junctions, indicating that many ancestral DSB ends are generated all-at-once. Second, short insertions originate from sites near the ancestral DNA ends including ssDNA overhangs, consistent with the observations in the bridge clone (**Figure 4** and **5**). Finally, amplified segments, including both large segments and short insertions, are either non-overlapping or nested, but rarely show partial overlap. The last feature is consistent with an origin from ss- or dsDNA fragments from a single chromatid.

8. Translocations and rearrangements of the broken ends of chromosome 4 in bridge clones

Primary clone	subclone	Homolog A	Homolog B
PC1a (F11)	a6	**10.54 Mb** (+) — acrocentric<br>**17.68 Mb** (+) — **chr14:20.05 Mb** (+)<br>**36.24 Mb** (+) — ? (4-cen)<br>**38.00 Mb** (+) — acrocentric<br>**38.76 Mb** (-) — acrocentric	intact
	a5	**39.62 Mb** (+) — telomere	**44.64 Mb** (+) —acrocentric<br>(13p,14p,21p) or chr9
	a4-2	**19.94 Mb** (+) — **chr19:41.87 Mb** (+)	
	a2	**25.73 Mb** (-) — acrocentric	
	a4-1	**47.84 Mb** (-)— acrocentric	**44.54 Mb** (+) — acrocentric (13p/21p)
	a4-3	**19.88 Mb** (+) — **49.17 Mb** (+);<br>(subclonal) 20.80 Mb (+) — ?	
	a3	**39.66 Mb** (+) — telomere (3x)<br>**27.51 Mb** (+) — acrocentric (3x)<br>39.95 Mb (-) — ?	**46.86 Mb** (+) — acrocentric
	a1-s2,7,8,11,12,16	**33.10 Mb** (+) — **164.21 Mb** (+)	variable (unmapped)
	a1-s13,14		**5.82 Mb** (-)—**129.61 Mb** (+)
	a1-s18		**47.08 Mb** (-)—**185.14 Mb** (+)
PC2b (F3)		**77,011** (+)—**138,703** (+) plus not mapped	variable/not mapped
PC1b (F2)		**36.64 Mb** (+) — **149.25 Mb** (+)	**47.88 Mb (+)** foldback, end not mapped
PC2a (F9)		**35.16 Mb** (-) — acrocentric	p-arm loss/centromeric
PC3a/3b(E5/E8)		mostly lost (not mapped)	intact
PC4a (K2)		**176.87 Mb** (-) — acrocentric	p-arm loss/centromeric
PC4b (K11)		**185.07 Mb** (-) — **chr6:94.91 Mb** (+)	intact
PC5a (O16)		**1.12 Mb** (+), **23.73 Mb** (+), **26.33 Mb** (+), , **31.59 Mb** (-) —acrocentric (14p);<br>**18.39 Mb** (+), **18.42 Mb** (-) — 4-cen;<br>**37.01 Mb** (+) — 7-cen/4-cen	**188.50 Mb** (-) — 4p-tel
PC6a (N11)		**188.88 Mb** (-) foldback, end not mapped	intact
PC6b (N6)		**188.70 Mb** (-) — 4p-tel.	intact
PC7a (B4)		**181.74** Mb (-) — ? (4-cen)<br>**181.06 Mb** (+) — ? (chrUn_KI270746v1)<br>**183.86 Mb** (+) — acrocentric	p-arm loss

The above table summarizes long-range translocations and foldback rearrangements of the broken chr4 identified in every bridge clone originally generated in Umbreit et al. (36). The primary clone IDs are according to the original names used in the paper; the names in parentheses are used as aliases in the Short Read Archive. Note the rarity of foldback junctions (PC1b, homolog B; PC6a, homolog A) in comparison to long-range translocation junctions. Also note the prevalence of junctions between chr4 and repeat sequences from the acrocentric arms, centromeric repeats, or other unmappable repeats. The probable locations of the repeats are inferred by aligning the junction/split sequences to the `CHM13` reference by `BLAT`.

We are able to determine several translocations at the ends of rearranged chr4 (**SI Figure 32** on next page). **A**. De novo telomere addition is identified in two subclones: In **a5**, telomeric repeats are inferred to be added to the end of the rearranged chromosome; in **a3**, telomeric repeats are inferred to be added to a duplicated end, so may be interstitial. **B**. Other repeats identified at the ends of broken chromosome 4. **C**. *Top*: An interchromosomal translocation that caps the broken end of chr4 in subclone **a4-2**. A short sequence mapped to a locus (19942536-3070) near the chr4 end at 19940488(+) is inserted into the junction between chr4 and chr19. *Bottom*: Another example of interchromosomal translocation that caps the broken end of chr4 in the PC4b bridge clone. In both examples, the translocated segments from chr19 (~17Mb) and chr6 (~75Mb) are beyond the maximum size of a single replicon; these translocations are most likely generated by fusions between the broken chr4 ends and a replicated DNA fragment that could have been initiated by strand invasion. See Rearrangement outcomes of break-induced replication. **D**. Three examples of intrachromosomal rearrangements that cap the broken ends on 4p by a 4q-terminal segment. In the first example, the broken end on the 4A homolog (copy number in red dots) joins a 4q terminal segment, with a breakpoint located within a pre-existing duplication; the junction sequence (in bold) contains an insertion that is partially mapped to the locus adjacent to the 4q breakpoint. Note the presence of 5-10bp short sequences (**1**,**2**,**1'**) that can be mapped to sequences near

the breakpoints. These features could reflect template-switching events after strand invasion; however, the duplicated 4q segment (~30Mb) can only be generated by conventional DNA replication as the examples shown in **C**. In the second example, the broken chr4B homolog (copy number in blue dots) has a large inverted duplication on the 4p terminus joining a 4q segment on the same homolog. In the last example, the broken 4B homolog has a short inverted duplication joining a large 4q terminal segment on the 4A homolog. The last two examples are identified in subclones of **a1** that have the same copy number of homolog A.

SI Figure 32: Examples of translocations of chr4 break ends

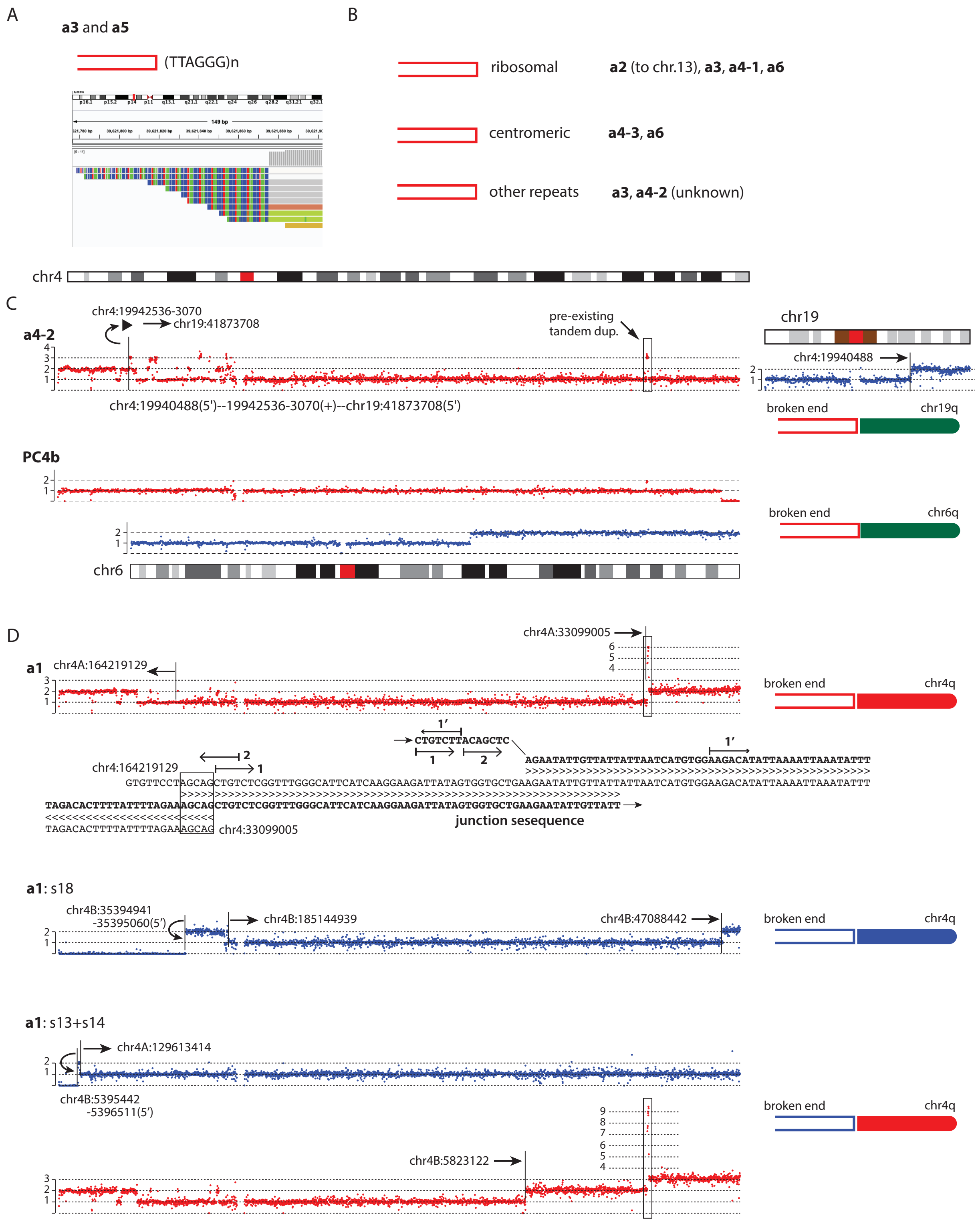

9. Footprints of breakage-replication/fusion in a post-crisis RPE-1 clone and a bridge RPE-1 clone

SI Figure 33: Copy number and rearrangement of chr11 in the X-29 clone from Maciejowski et al. (18)

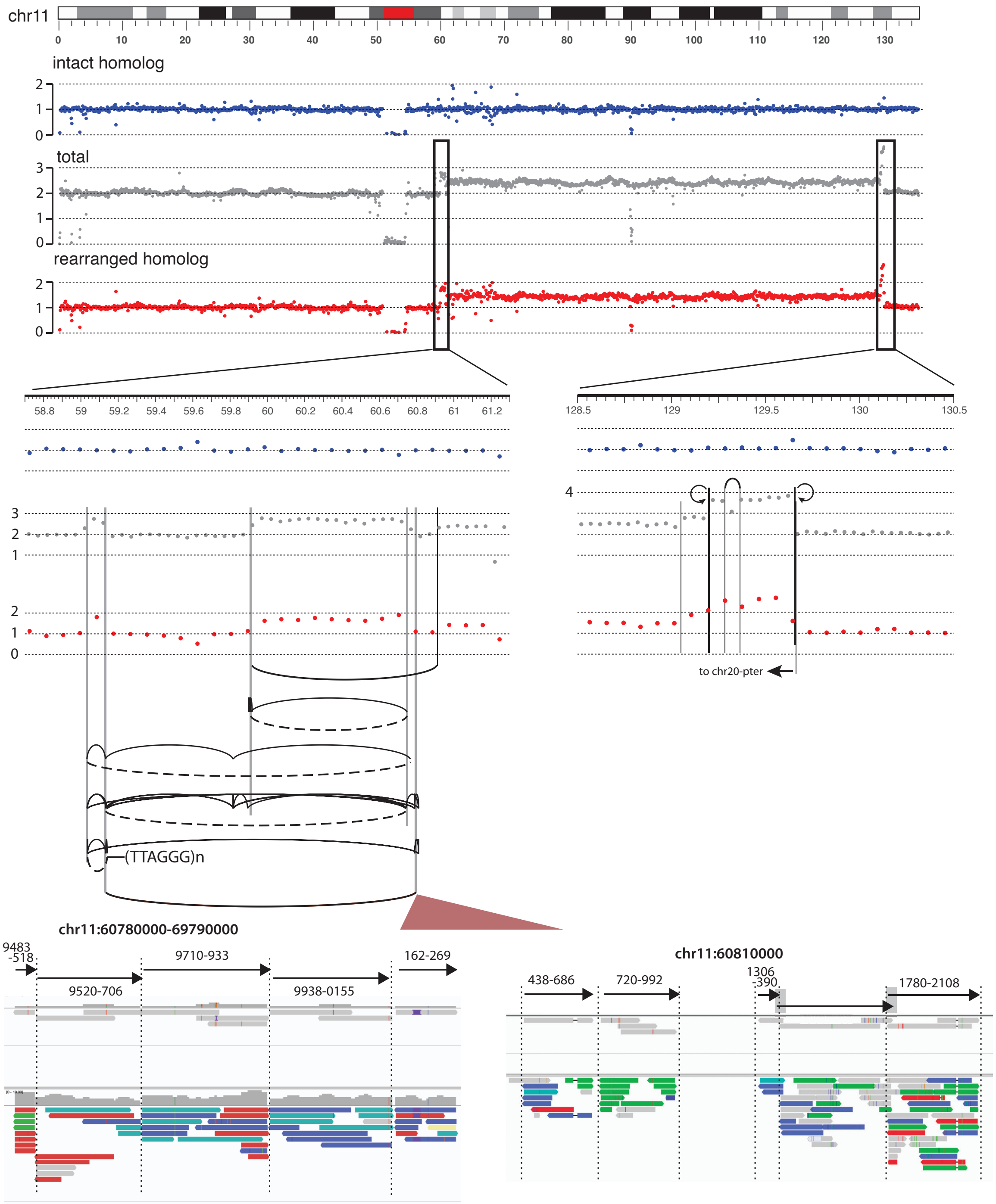

Top: Haplotype-specific (90kb bins) and total DNA copy number (50kb bins) of chr11 in the X-29 clone.
Middle: Zoomed-in view of copy-number transitions near 60Mb and 130Mb. Breakpoints near 60Mb are shown in **SI Figure 34**.
Middle Bottom: Rearrangement junctions at 60Mb and 130Mb, left panel is shown **Extended Data Fig. 9b**.
Bottom: Two hotspots with tiling insertions near 60.78Mb, and 60.81Mb. The top IGV snapshots show contigs assembled from split and discordant reads shown in the bottom. Additional hotspots include 59.03Mb, 59.13-59.14Mb, 59.82Mb, 59.90-59.91Mb,

60.74Mb, and 60.79-60.81Mb. Insertions near 59.03Mb and 59.13Mb are shown on the next page.

Although we cannot determine the structure of duplicated segments based on bulk sequencing data alone, we can determine the copy number of each breakpoint. Because all copy-number transitions occur to a single parental chromosome based on the haplotype-specific coverage, we use the total coverage to derive more accurate quantification. The copy-number gains are approximately 0.8x (59.03-59.13Mb and 59.9Mb-60.74Mb) and 0.4x (60.76-60.79Mb and 60.9Mb-130Mb).

The copy-number transitions are associated with the following breakpoints (**SI Figure 34**): paired parallel breakpoints at 59.03Mb(+), 59.13Mb(-), 59.91Mb(+), and 60.74Mb(-); single breakpoints at 60.77Mb(+), 60.80Mb(-), and 60.90Mb(+). For single breakpoints, their copy number is directly determined by the copy-number difference (~0.4). We can then use the copy number of single breakpoints to determine the copy number of their rearrangement partners (red dashed lines for junctions with insertions, solid line for simple junctions). A simple calculation indicates that all the breakpoints have the same copy number transition ~0.4.

Two possible configurations of rearranged segments consistent with the copy number of breakpoints are shown on the bottom. For the copy-number gain between 59.03Mb and 59.13Mb, we identify adjacent breakpoints on both sides, each with short insertions mapped to adjacent regions. It is likely that the two segments show similar breakpoint pairing as the **G1/G2** segments in bridge clone **1a** (**Fig. 5a**). We cannot verify the pairing of breakpoints on the duplications between 59.91Mb and 60.74Mb.

SI Figure 34: Inferred segments of copy-number gains in X-29 from Maciejowski et al. (18)

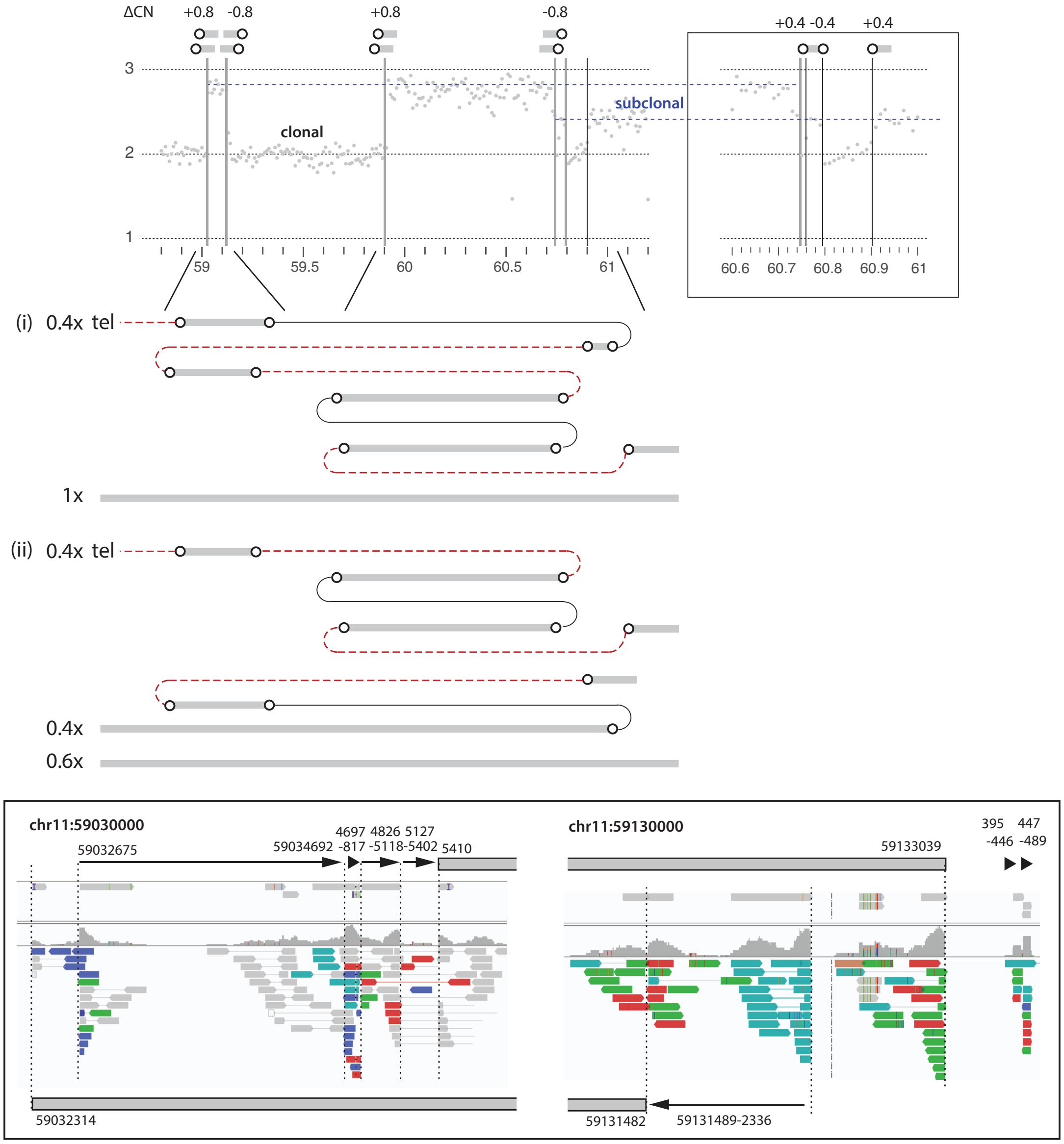

SI Figure 35: Copy-number variation and segmental structure of rearrangement in Primary Clone 5a from Umbreit et al. (36)

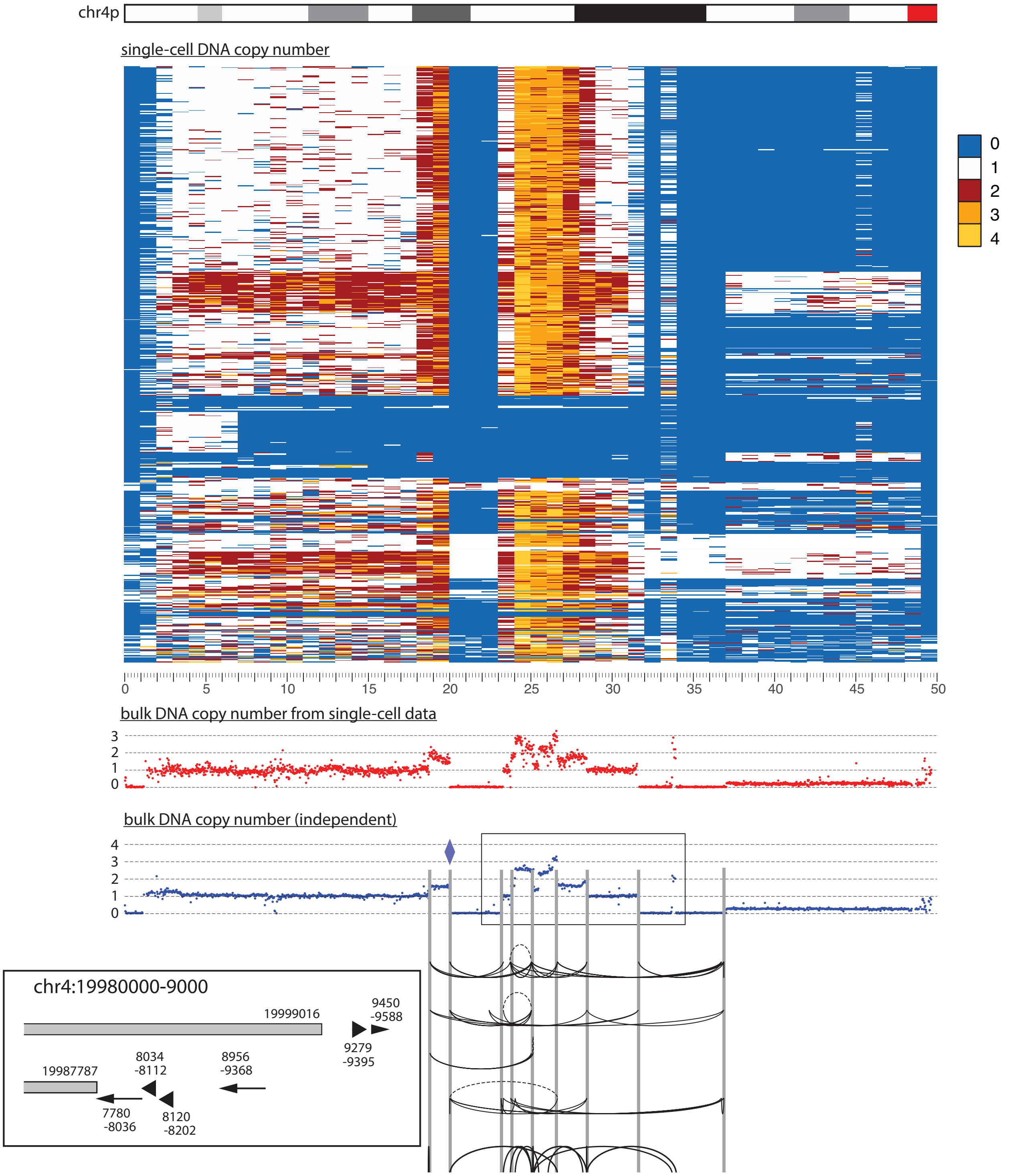

Top: Single-cell copy-number profiles of the altered haplotype.
Middle (red): Bulk DNA copy number calculated from the single-cell data.
Middle bottom (blue): Bulk DNA copy number calculated from bulk DNA sequencing. A zoomed view of copy-number transitions between 22 and 35Mb is shown in the next page.
Bottom: Simple and complex rearrangement junctions, similar to **Extended Data Figure** 9. Diamond represents a hotspot of insertions near 19.98Mb that are shown in the inset.

SI Figure 36: Inference of breakpoint copy number based on segmental copy number transitions

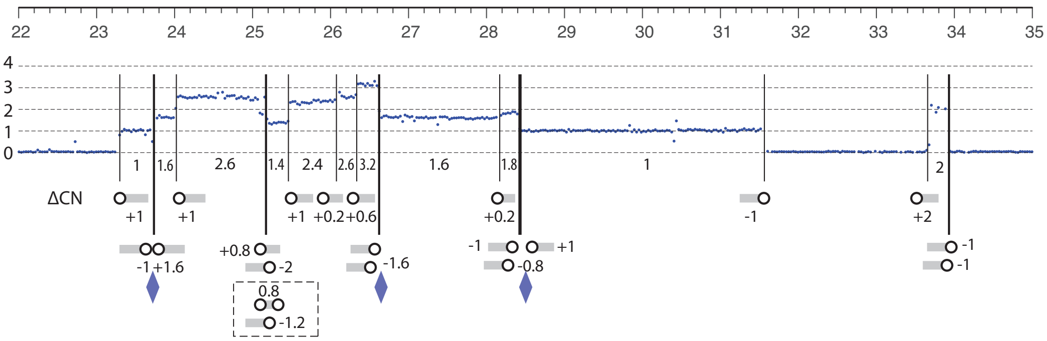

We can also draw inferences about the segmental structure of duplications based on the copy-number transitions at rearrangement breakpoints (**SI Figure 36**) and their pairing in the rearrangement junctions.

For unpaired breakpoints, their copy number states are determined directly from the copy-number differences:

- 23.29Mb(+1), 24.01Mb(+1), 25.46Mb(+1), 26.04Mb(+0.2), 26.33Mb(+0.6), 28.18Mb(+0.2), 31.59Mb(-1), 33.68Mb(+2).

We then use the junction information to determine the copy number of adjacent breakpoints. There are two adjacent gapped breakpoints at 25.014Mb(-) and 25.018Mb(+): based on the junction between 25.018Mb(+) and 23.29Mb(+1), we determine its copy number to be 25.018Mb(+1); because there is no net copy-number change at 25.01Mb, we further determine the breakpoint at 25.014Mb to be 25.014Mb(-1). From the junction between 25.014Mb(-1) and 23.72Mb(-), we determine the latter to be 23.72Mb(-1); finally, because the net copy-number change at 23.72Mb is +0.6, we determine the copy number of 23.73Mb to be 23.73Mb(+1.6). Together, we have:

- 23.72Mb(-1), 23.73Mb(+1.6), 25.014Mb(-1), 25.018Mb(+1).

We next consider the copy-number transition at 25.17Mb with two breakpoints 25.170Mb(+) and 25.173Mb(-). The breakpoint 25.173Mb(-) forms a junction with the breakpoint 33.68Mb(+2), therefore its copy number is 25.173Mb(-2); based on the segmental copy-number difference, we determine 25.17Mb(+) to be 25.17Mb(+0.8). Note that this inference is independent of the *cis* or *trans* relationship between the two breakpoints at 25.17Mb. The junction between 25.17Mb(+0.8) and 28.417Mb(-) then determines the latter to be 28.417Mb(-0.8). Finally, the copy-number transition at 28.42Mb has a net copy-number change of -0.8 that is equal to the copy number of 28.417Mb(-0.8); therefore, the two remaining breakpoints 28.423Mb(-) and 28.424Mb(+) must have the same copy number. Because these two breakpoints form junctions with 33.93Mb(-) and 33.94Mb(-), the copy number of the latter two breakpoints must be equal; therefore, the latter two breakpoints must each have copy number -1 to balance the copy-number gain at 33.68Mb(+2). Together, we have

- 25.170Mb(+0.8), 25.173Mb(-2), 28.417Mb(-0.8), 28.423Mb(-1), 28.424Mb(+1), 33.93Mb(-1), 33.94Mb(-1).

The total copy number of two breakpoints at 26.61Mb(-) and 26.62Mb(-) is -1.6 but the individual copy number state cannot be determined. There is a short internal deletion of 28,419,969-28,420,319 whose copy number cannot be determined.

The breakpoint copy number can be used to determine the structure of rearranged segments. For example, the breakpoint at 23.72Mb(-1) has to be in *cis* with the breakpoint at 23.29Mb(+1) because the flanking sequence to the left of 23.29Mb is deleted. The identical copy number of these two breakpoints further suggests that they are retained only in this segment. Similar reasoning can be applied to determine the presence of one unique segment between 28.42 and 31.59Mb and two unique (sister) segments between 33.68 and 33.93Mb. Moreover, the breakpoint at 28.18Mb(+0.2) has to be in *cis* with either 28.417Mb(-0.8) or 28.423Mb(-1).

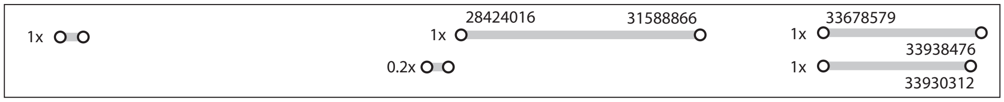

For the remaining segments, their breakpoints cannot be determined.

10. Breakpoints of rearranged segments in bridge clone a

In this section we show how the breakpoints of rearranged segments (**Extended Data Figure 4**) in bridge clone **a** are determined from copy-number variation in the subclones. We present the segmental copy-number analysis for the following regions. **A**:pter-16.5Mb; **B**:17-22Mb; **C,D**:24.9-27.6Mb; **E-I**:24.9-26.3Mb; **L,M,N**:41-46Mb; **O,P,Q**:46-47Mb; **S,T,U**:48-49Mb; **R**:47-48Mb; **J,K**:39-41Mb. The sequence coverage in region **V**:49.17-49.32Mb is unreliable and we determine the V segments solely based on the rearrangement breakpoints.

For each region, we first show the DNA copy number of haplotype A (25kb bins) in each subclone (**a1-a6**). For regions **O-U**, we also show the total coverage (10kb bins) to better resolve copy-number transitions. The copy-number data are shown together with the segments of copy-number alterations (identical segments are shown only once). The segmental breakpoints and the copy number state of each segment in each subclone are summarized in **Supplementary Table 8**. We then summarize segments identified in all the subclones and discuss how the segments are determined from the copy-number data. Finally, we show the order of these segments in the rearranged segments determined from the copy number and the rearrangement junctions. The rearrangement junction data are presented in **Supplementary Table 9** and insertions at rearrangement junctions are listed in **Supplementary Tables 13-16**.

We use the segments from regions **C** and **D** as an example to demonstrate how the ancestral DNA fragments can be inferred from the segments of rearrangement. All the segments and their evolutionary classification are shown in **Supplementary Table 10**. Special considerations are needed to infer the structure of duplicated segments in region **R** and both the structure and the rearrangement of segments from region **J**. These analyses are presented in detail.

Subclones are sorted by the number of segments they retain: **a1** has the fewest and **a6** has the most. Segments are colored by the subclone where they are first identified (usually present at the **lowest** copy-number state): gray for **a1**; yellow for **a2** and **a3**; orange for **a4**; green for **a5**; blue for **a6**.

Segments are named by the same convention as in **Extended Data Figure 4**:

i Segments inferred to have been derived from sub-fragments of a single ancestral fragment are appended with ‘a’,‘b’, etc. (e.g., **Ba-Bd**).

ii Segments inferred to have been derived from sister DNA generated by replication of a single ancestral fragment are labelled ‘1’ and ‘2’ with ‘1’ being the longer segment. These segments are bounded by flushed or adjacent parallel breakpoints.

iii Partial duplications (i.e., duplicated segments that are truncated relative to the ancestral segment) have lowercase letters, e.g., **bc** is a truncated duplication of **Bc**.

iv When more than one truncated segments are present, they are distinguished using ‘_1’, ‘_2’, e.g., **r1_1**, **r1_2**.

SI Figure 37: Subclonal copy-number variation in bridge clone **a** in region A:p-ter–16.5Mb

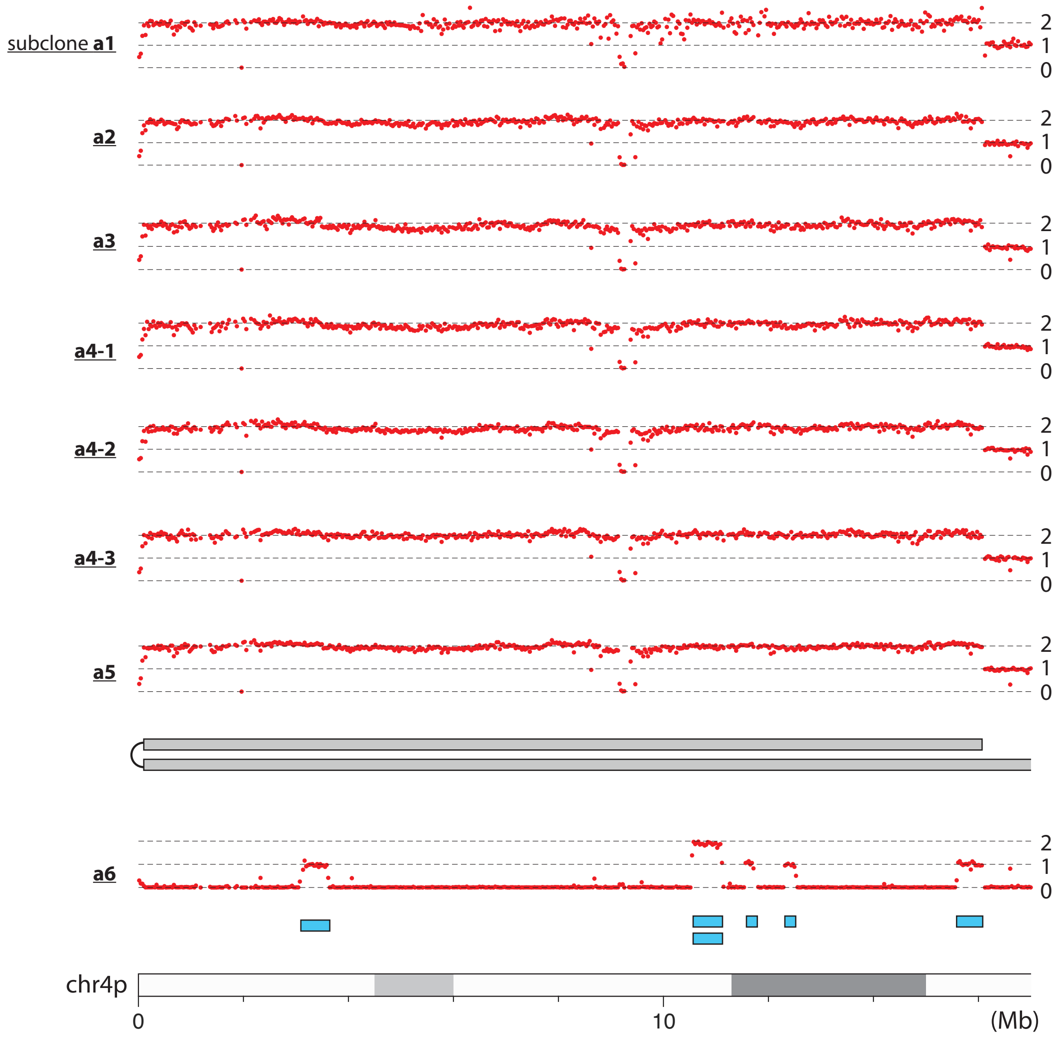

SI Figure 38: Segments of copy-number variation in region A:p-ter–16.5Mb

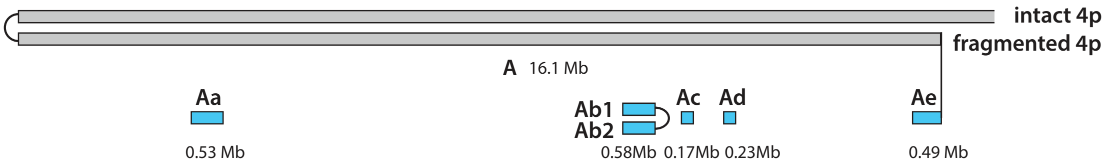

a1-a5 contain an extra copy of segment **A**:pter-16.5Mb; segments in **a6** are generated by a secondary chromothripsis event that creates six subfragments from segment **A**, including a pair of duplications **Ab1/Ab2** that are connected by a foldback junction. This secondary chromothripsis also involves chr14q.

SI Figure 39: Subclonal copy-number variation in bridge clone **a** in region B:17-22Mb

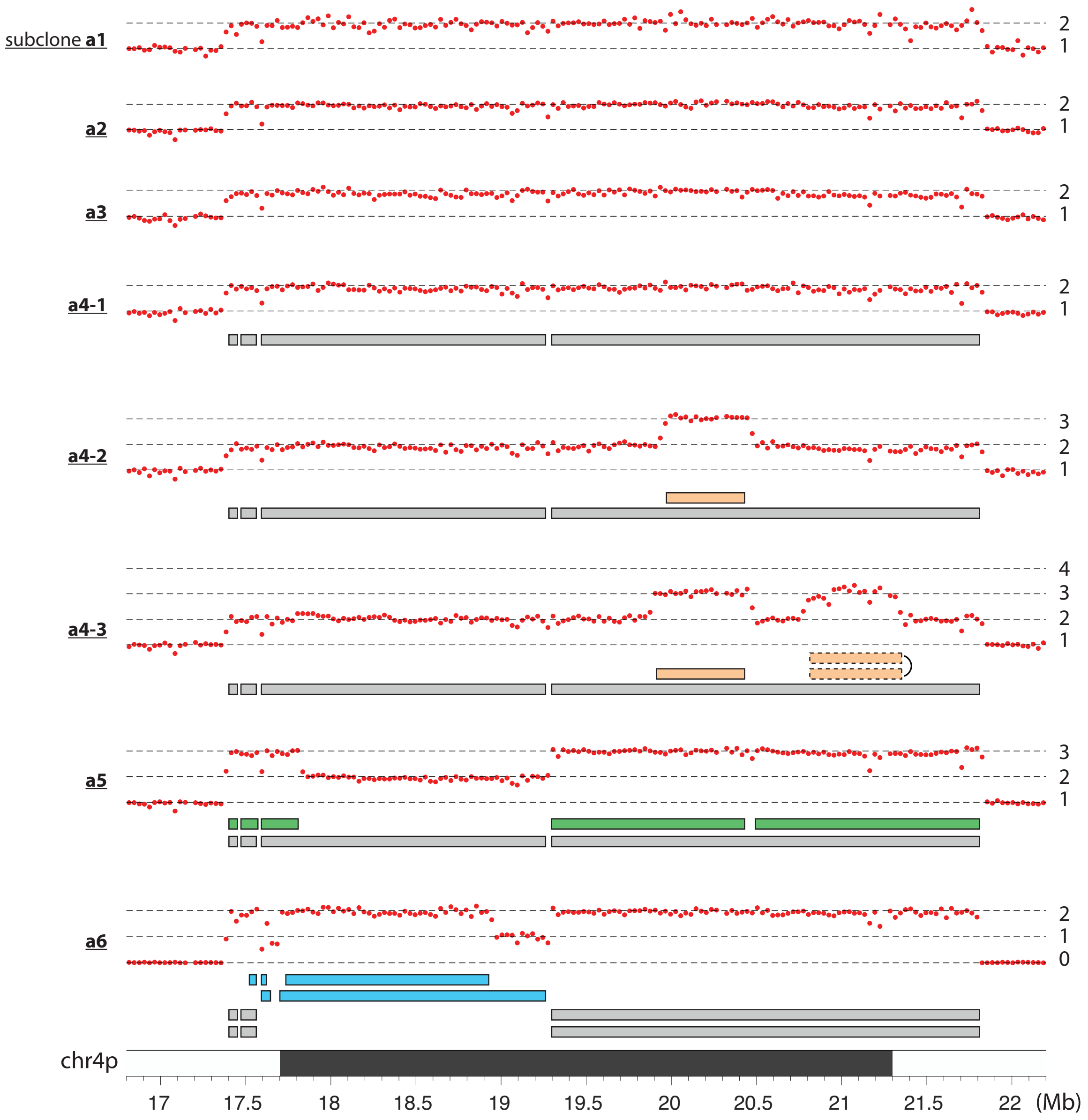

Cheng-Zhong Zhang, Carlos Mendez-Dorantes, Kathleen H. Burns, David Pellman

SI Figure 40: Segments of copy-number variation in region B:17-22Mb

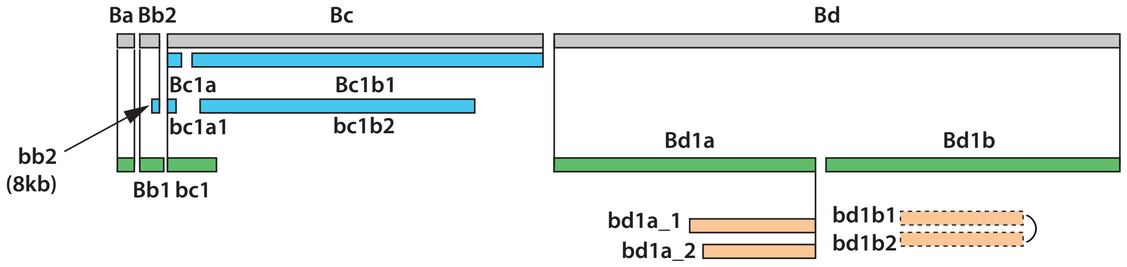

Segments **Ba**,**Bb2**, and **Bd** are determined in subclone **a6** where the flanking sequences are deleted. The same reasoning leads to the determination of *cis* segments **Bc1a** (17.60-17.66Mb) and **Bc1b1** (17.68-19.26Mb), and their descendants/cousins **bc1a1** (17.60-17.63Mb) and **bc1b2** (17.69-18.95Mb). We note that the telomeric (left) breakpoints of **Bc1b1** and **bc1b2** are adjacent (17.68 and 17.69Mb); their pairing with the centromeric (right) breakpoints (18.95 and 19.26Mb) cannot be uniquely assigned but this uncertainty does not impact the relationship between **Bc1b1** and **bc1b2**. The **Bc1a/Bc1b** segments determine the ancestor segment **Bc**. Segment **Bb1** is defined in subclone **a5** as a sister of **Bb2** based on adjacent centromeric breakpoints. Segments **Ba-Bd** account for the copy number gain between 17.38 and 21.83Mb in subclone **a1-a3** and **a4-1** besides an intact copy of 4p.

Assuming the copy of intact 4p is preserved in all subclones except **a6**, we can then define the remaining segments as follows: Segments **bc1** and **Bd1a/Bd1b** account for the additional copy-number gains in **a5**. The copy-number gains near 20Mb in **a4-2** and **a4-3** are attributed to segments **bd1a_1** and **bd1a_2** that are truncated copies of **Bd1a**. Both segments are inferred to be located near the ends of the rearranged chromosome. The copy number gain near 21Mb in **a4-3** is attributed to duplications **bd1b1/bd1b2** that are joined by a foldback junction. **a6** also contains a small (8kb) segment **bb2** that shares the right boundary with **Bb2**.

We then determine the segmental structure of rearranged DNA combining segmental breakpoints and junctions.

For **B** segments, we have the following synteny

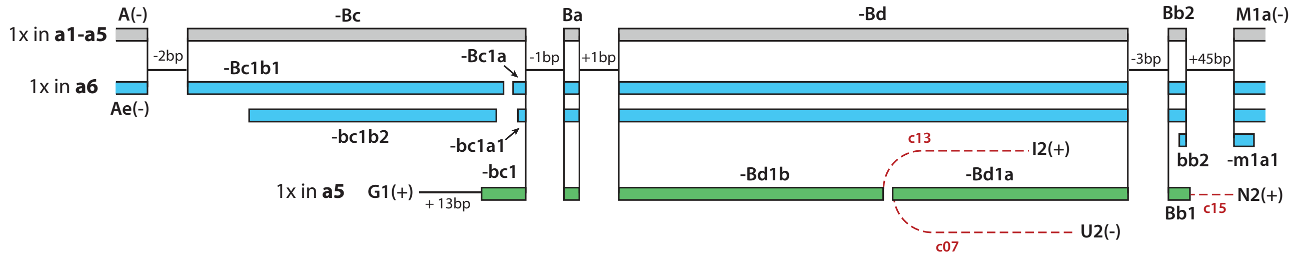

We have labelled microhomology ('-') or insertion ('+') features at the junctions. Note that junctions generated before duplication (shared by two or more segments) are mostly blunt (having little homology or insertion).

In subclone **a6**, several breakpoints cannot be mapped exactly (the junction sequences suggest locations in the acrocentric arms); the long-range linkage between segments **-bc1a1/-Bc1a**, **Bb2/bb2**, and **-M1a/-m1a1** also cannot be determined. One possible arrangement of the rearranged segments is shown below.

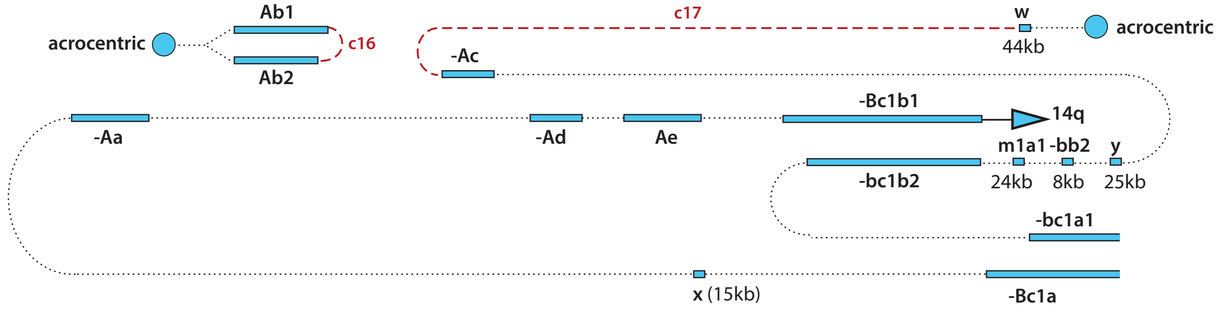

SI Figure 41: Subclonal copy-number variation in bridge clone **a** in region C:24.9-26.3Mb and D:26.7-27.6Mb

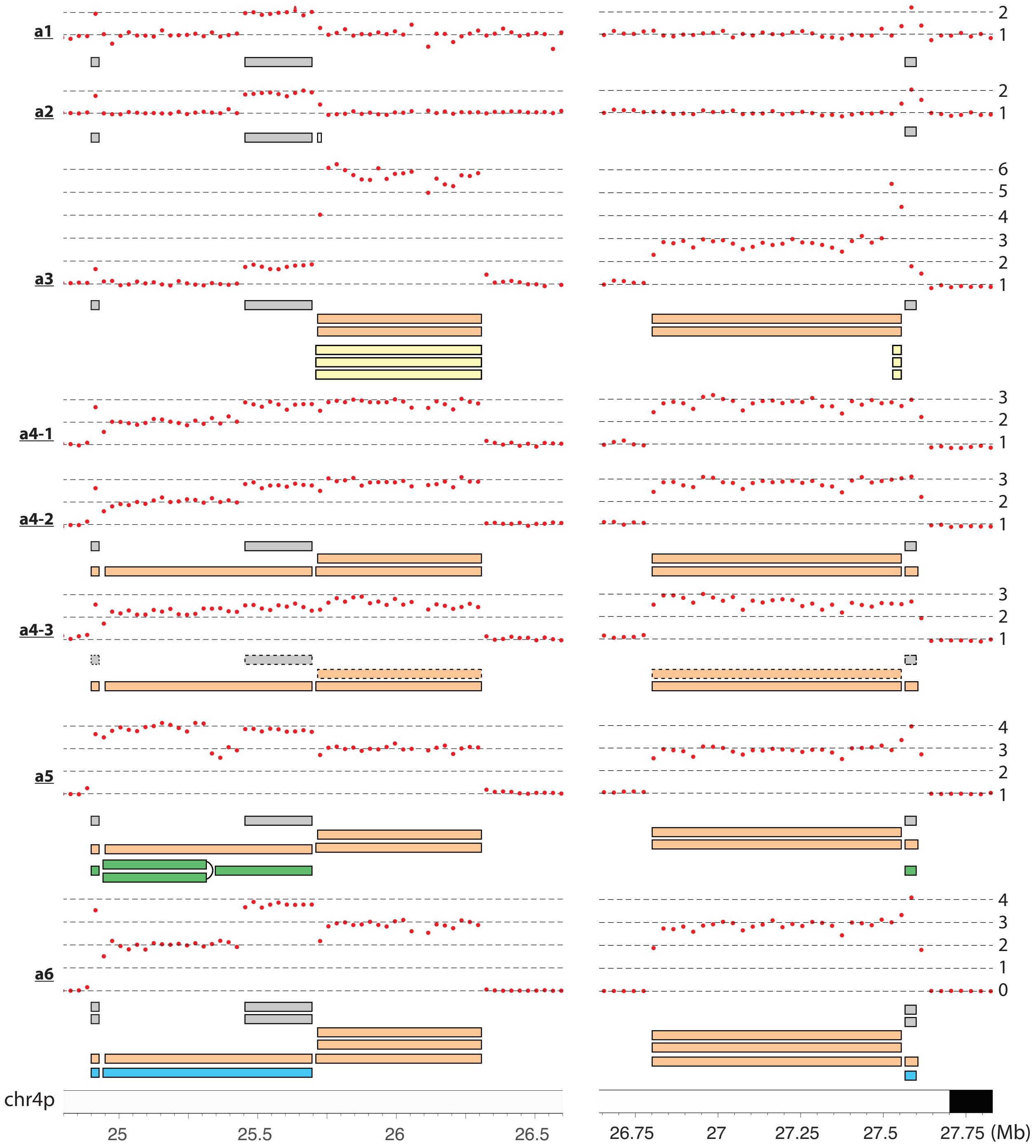

SI Figure 42: Segments of copy-number variation in region C:24.9-26.3Mb and D:26.7-27.6Mb

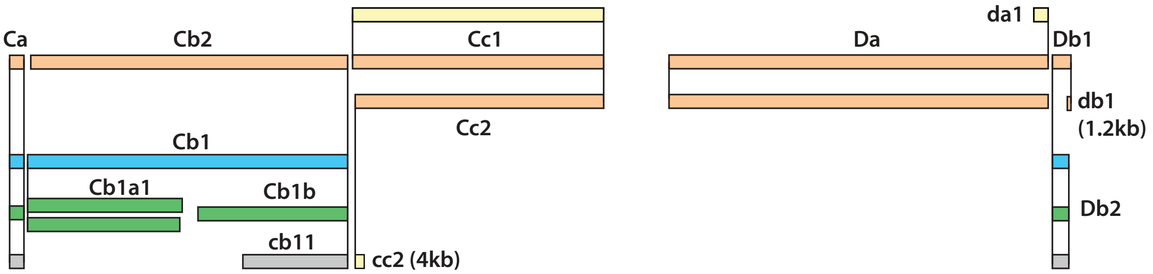

First, we determine the breakpoints of the following segments and their copy number states in subclone **a6**: **Ca**(4x), **Cb1**(1x)/**Cb2**(1x)/**cb11**(2x), **Cc1**(1x)/**Cc2**(2x), **Da**(3x), **Db1**(1x)/**Db2**(3x). Numbers in parentheses indicate segmental copy number. These segments are separated by complete deletions in subclone **a6**. We then determine the copy number of these segments in each subclone as well as additional segments that are private to each subclone. The segmental copy-number data are shown in **SI Figure 43A**. From these data, we can identify ancestral breakpoints or segments (**SI Figure 43B**) and infer the structure of ancestral DNA fragments (**SI Figure 43C**). Finally, based on the segmental structure of rearranged DNA determined from the rearrangement junctions, we infer there were four different rearranged DNA molecules in the ancestral rearranged chromosome (**SI Figure 43D**): **Cb2→-Cc1**, **Cb1→F1**, **db1→-Cc2**, and **cb11→F2**.

SI Figure 43: Inference of ancestral DNA fragments from subclonal copy-number variation in region C:24.9-26.3Mb and D:26.7-27.6Mb

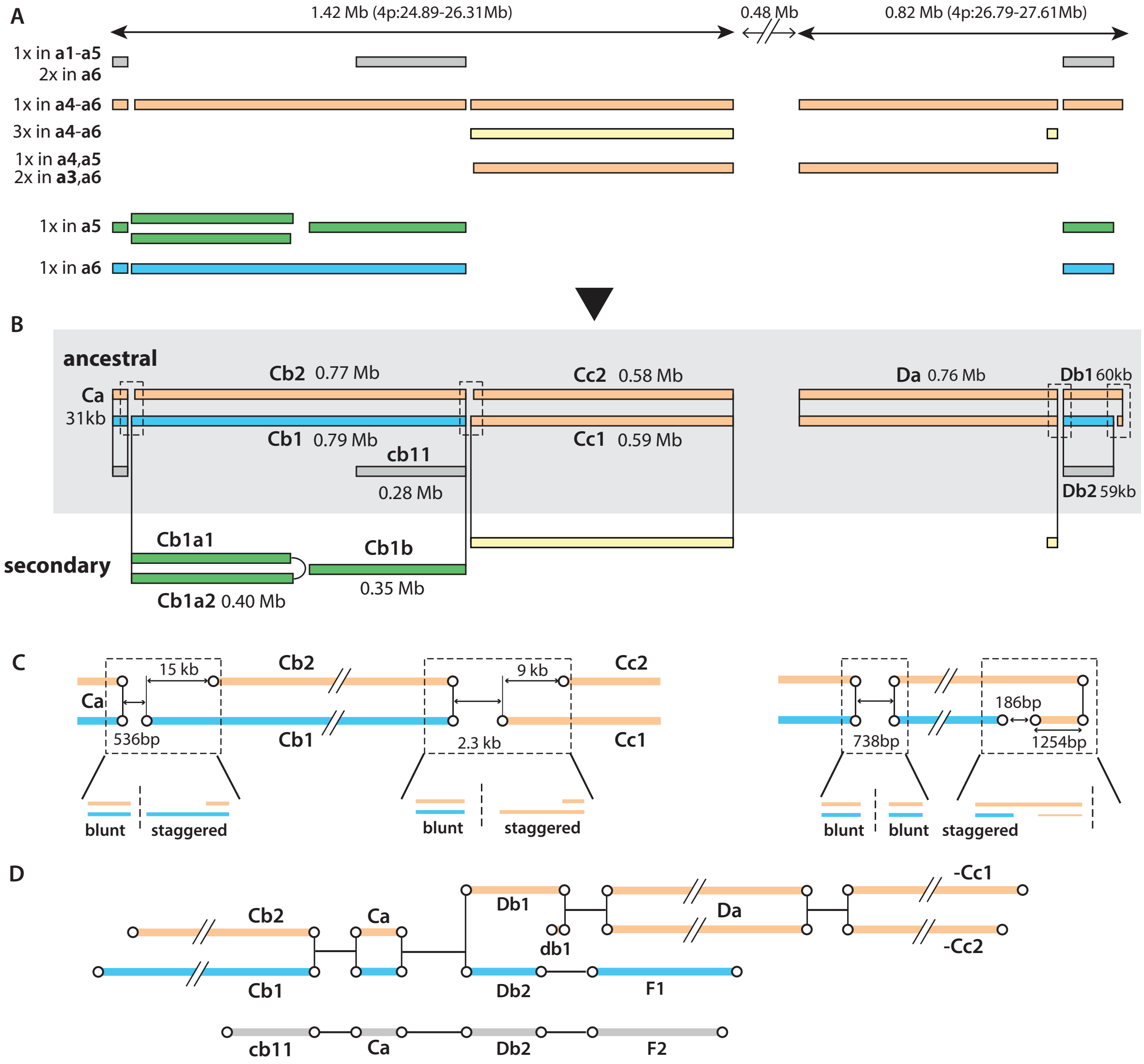

SI Figure 44: Subclonal copy-number variation in bridge clone **a** in region 30-39Mb

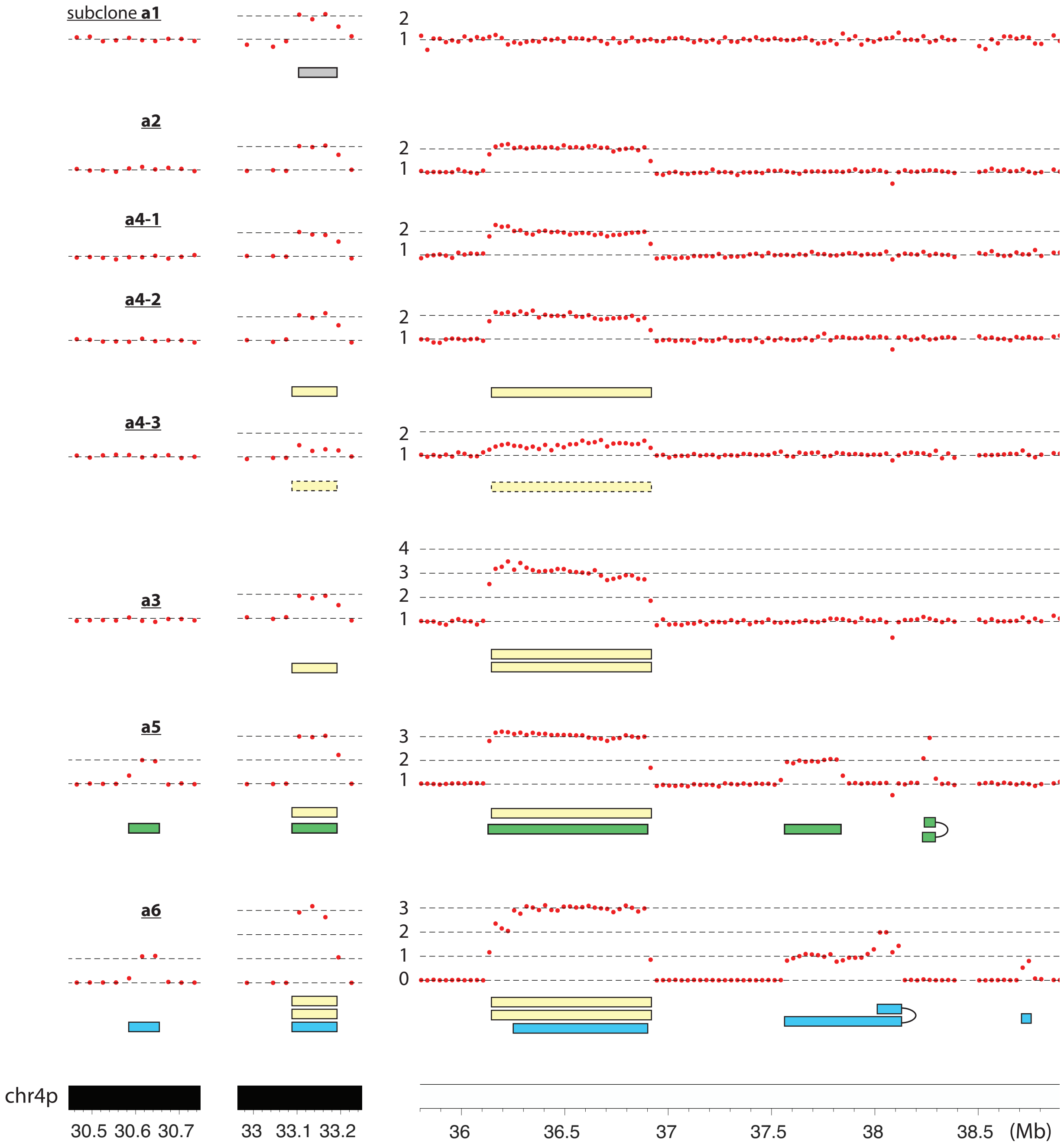

SI Figure 45: Segments of copy-number variation in region 30-39Mb

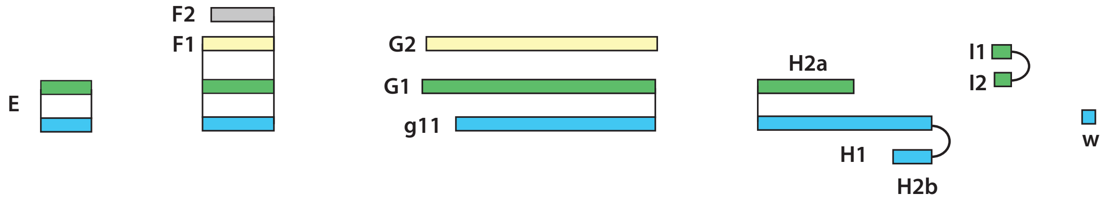

From subclone **a6** we determine the breakpoints of the following segments: **E**(1x), **F1**(3x), **G2**(2x), **g11**(1x), **H1**(1x) and **H2b**(1x). Numbers in parentheses are the copy number of each segment. **H1** and **H2b** have adjacent centromeric (right) boundaries are joined together by a foldback junction with insertions. There is also a 40kb-segment **w**:38.71-38.75Mb that is private to **a6**.

Segment **G1**, with breakpoints identified in subclone **a5**, is determined to be a sister of **G2** based on adjacent breakpoints at both centromeric and telomeric boundaries. See **SI Figure 5** for the rationale for this inference.

Subclone **a5** also contains a segmental gain between 37.55 and 37.83Mb that shares an identical breakpoint as segment **H1**: We infer this copy-number gain to be due to a segment **H2a** that may have been derived from the same ancestral segment (**H2**) that gives rise to **H2b**, and **H2** and **H1** are sister segments generated by replication.

The two copy gain near 38.2Mb in subclone **a5** is attributed to a pair of sister segments **I1/I2** that are joined by a foldback junction.

Segment **F2**, which is retained only in subclone **a1**, is inferred to be a sister of **F1** based on adjacent breakpoints at the centromeric boundary(right).

The joining pattern of these segments in the rearranged chromosome is shown below. Note the synteny between **E** and **H** that supports the inference of **H2a** and **H2b** derived from a single ancestor **H2**. In subclone **a4-3**, segment **G2** is lost from a fraction of cells; similar loss is also seen for the flanking segments **O** and **J2**, indirectly confirming the assembly.

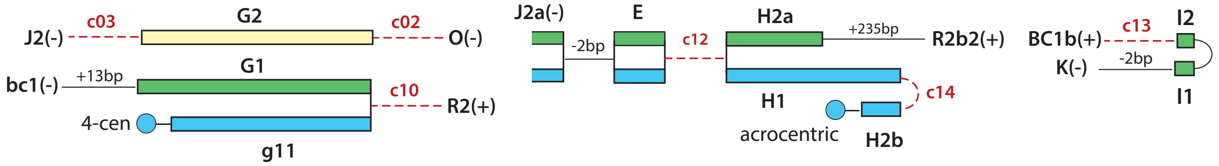

Segment **w** is included in the rearranged **A** segments in subclone **a6**, **F** segments will be treated together with the flanking segments at the end of this section.

SI Figure 46: Subclonal copy-number variation in bridge clone **a** in region 41-46Mb

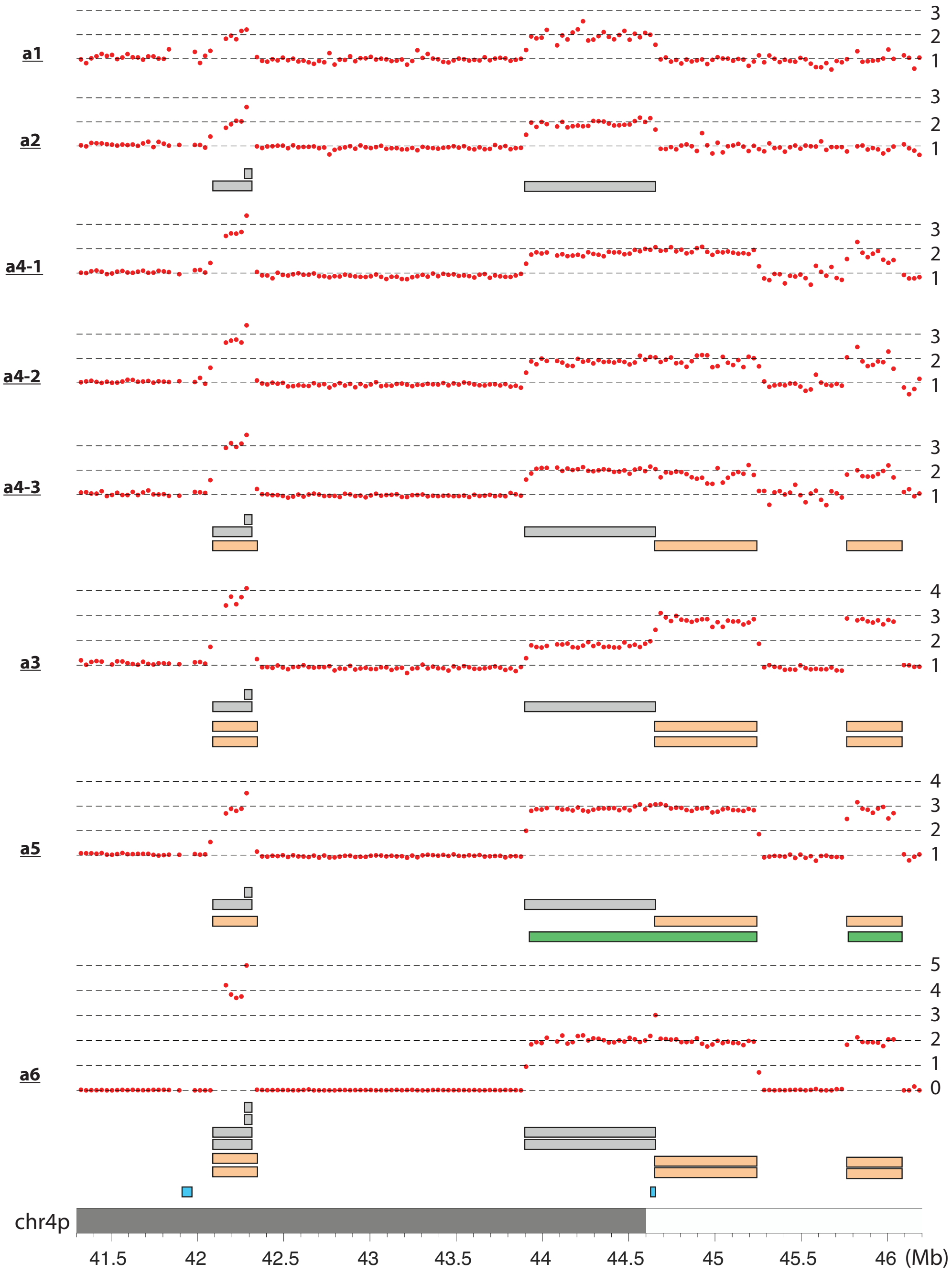

SI Figure 47: Segments of copy-number variation in region 41-46Mb

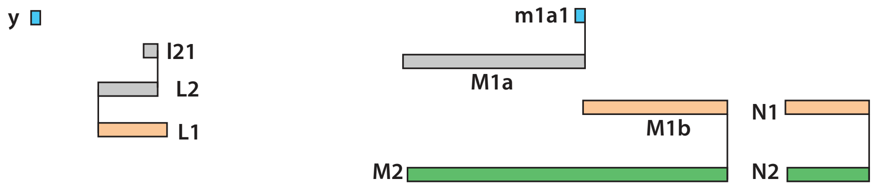

From subclone **a6** we determine the breakpoints of the following segments: **L1/L2/l21**, **M1a/M1b**, and **N1** (all 2x). The **N2** segment is defined in subclone **a5**; **N1/N2** are inferred to be sisters as they have adjacent breakpoints on the telomeric side. In subclone **a5** we also identify a segment **M2** that is inferred to be a sister of the ancestor of **M1a/M1b**. We further infer the partial overlap between **M1a** and **M1b** to have been generated by a replication-bypass mechanism (cf. **Extended Data Fig. 8b,c**).

There are two **a6**-private segmental gains: one (**y**) at 41.91-41.94Mb (25kb), the other (**m1a1**) at 44.64-44.66Mb (24kb). They are included in the rearranged **A** segments in subclone **a6**. The arrangement of **L**, **M**, and **N** segments in the rearranged chromosome will be presented later.

SI Figure 48: Subclonal copy-number variation in bridge clone **a** in region 46-47Mb

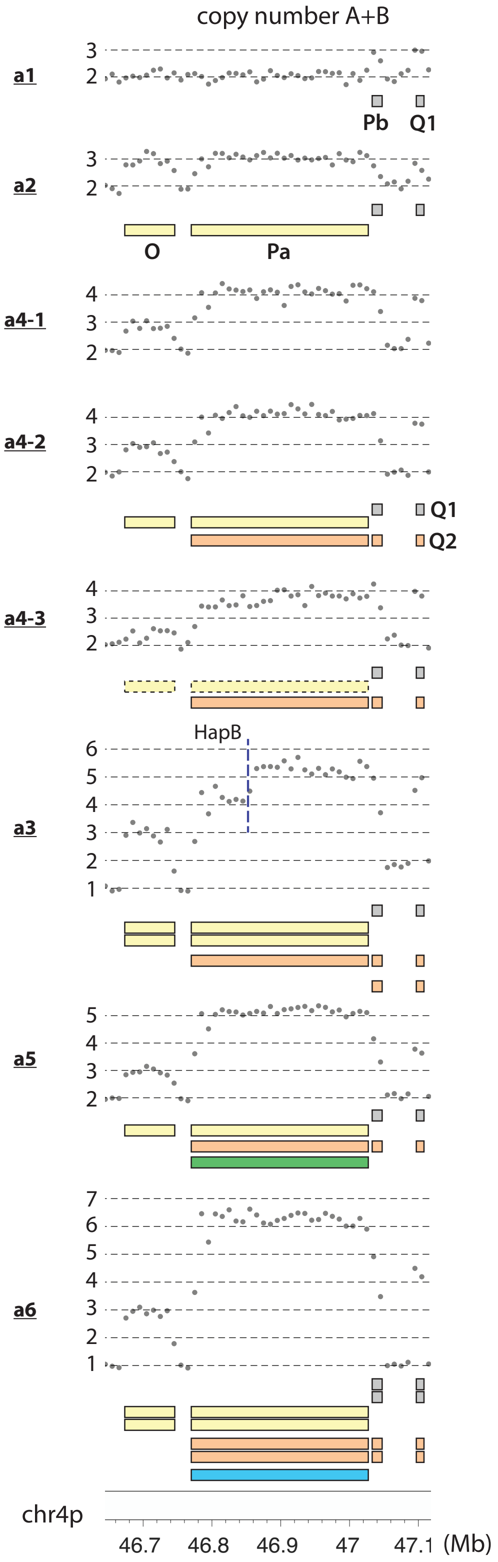

Cheng-Zhong Zhang, Carlos Mendez-Dorantes, Kathleen H. Burns, David Pellman

SI Figure 49: Subclonal copy-number variation in bridge clone **a** in region 48-49Mb

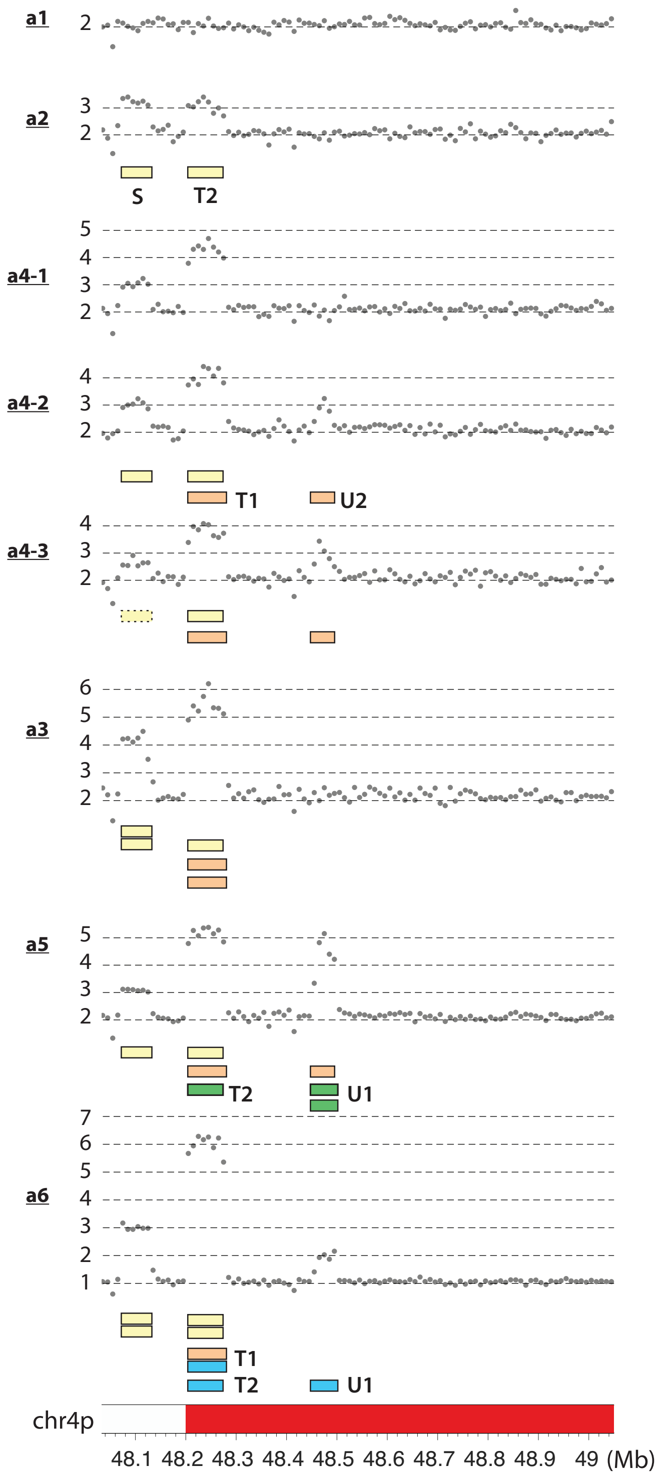

SI Figure 50: Segments of copy-number variation in regions 46-47Mb and 48-49Mb

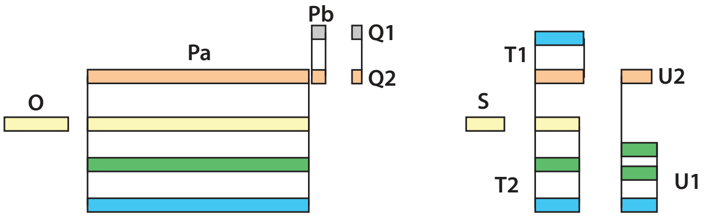

To determine segmental copy-number variation in 46-49Mb, we use both the total DNA copy number (left) for breakpoint identification and haplotype-specific copy number (right) for breakpoint phasing. We note that subclone **a6** has an intact copy of homolog B. (1) From subclone **a6** we determine the breakpoints of the following segments: **O**(46.67-46.74Mb;2x), **Pa**(46.78-47.029Mb;5x), **Pb**(47.03Mb-47.047Mb;4x), **S**(48.07-48.13Mb;2x), and **U1**(48.46-48.50Mb;1x). (2) We can also determine the breakpoints of **Q1/Q2** (47.09-47.11Mb) and **T1/T2**(48.20-48.28Mb) from subclone **a6**, but their copy-number states. The copy-number states of **Q1/Q2** segments in **a6** can be determined based on the copy number of the flanking segments in the rearranged chromosome (shown below). The copy-number states of **T1/T2** segments in subclone **a6** will be determined later. (3) The breakpoints of **U2** (sibling of **U1**) are determined in subclone **a4** and **a5**.

The assembly of segments **L-N**, **Pb**, **Q**, and their copy-number states in each subclone are shown below. Note that the linkage between **M1a/m1a1** and **Bb2/bb2** in subclone **a6** cannot be definitively resolved: it is possible that **m1a1:bb2** (total size 32kb) is present as an overlapping duplication generated by replication bypass. The copy-number states of segments **L1/L2/l21** and **Q1/Q2** in subclone **a6** are determined from the copy number of **M1a** and **M1b** (both 2x).

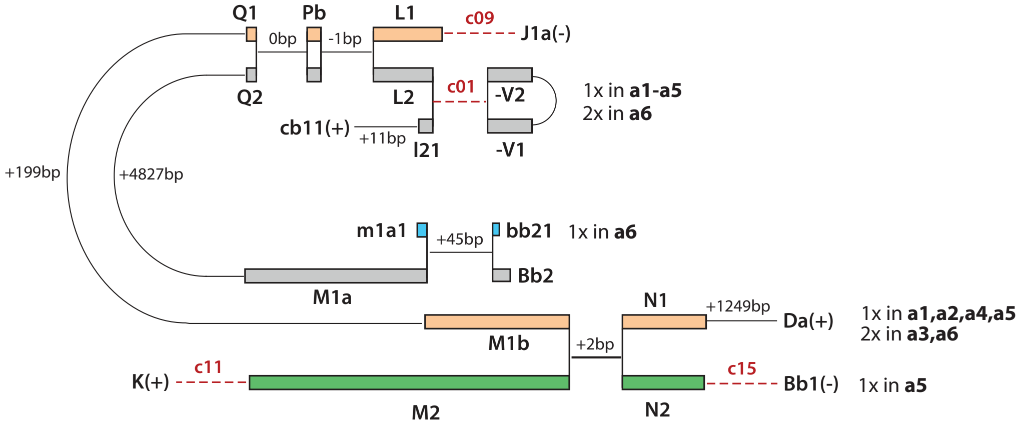

SI Figure 51: Subclonal copy-number variation in bridge clone **a** in region R:47-48Mb

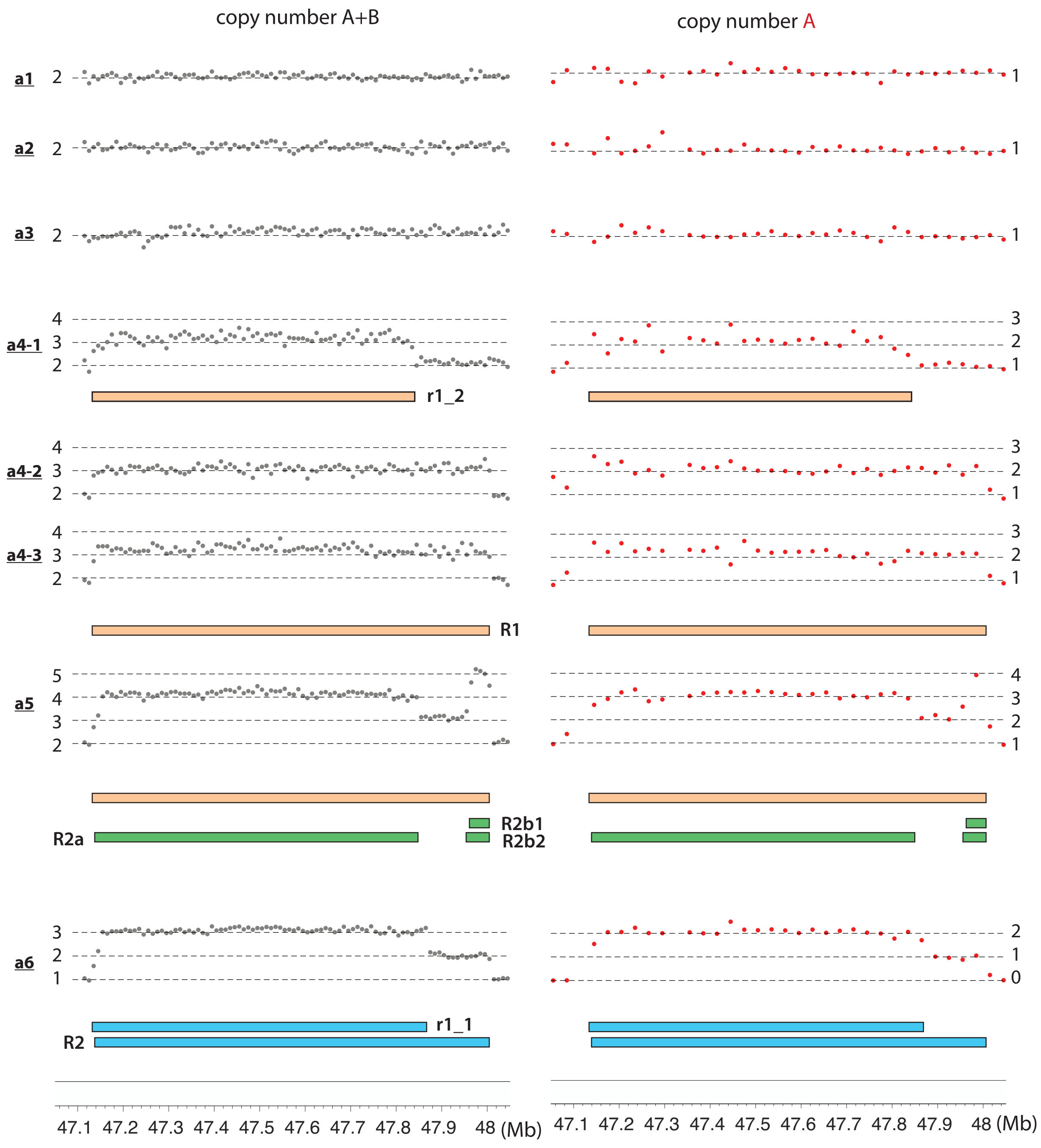

From the segmental copy number in subclone **a4-2** and **a4-3**, we can determine the breakpoints of segments **R1** (47.13-48.01Mb). A truncated segment **r1_2** (47.13-47.84Mb) accounts for the gain in subclone **a4-1**. Based on breakpoint junctions in subclone **a4**, we can assemble the following compound segment: **Cb2(+):R1:U2:Bd1a(-)**. From the segmental copy number and breakpoints in **a5** and **a6**, we infer the presence of a sister segment **R2**: **R1** and **R2** share the same centromeric (right) breakpoint but have distinct, adjacent telomeric (left) breakpoints. The duplications in **a5** and **a6** can have two configurations (#1 and #2 as shown in **SI Figure** 52).

SI Figure 52: Segments in region R:47-48Mb

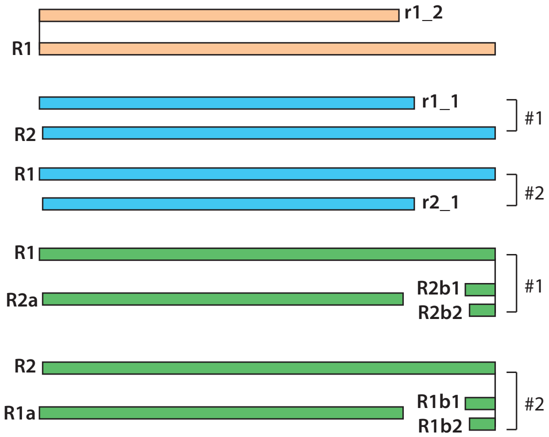

To distinguish between these two configurations, we need to consider the copy number of the flanking segments in the rearranged chromosome. From the compound segment **Cb2(+):R1:U2:Bd1a(-)** determined in subclone **a4**, we can determine the copy number of the **R1** segment in subclone **a5** and **a6** from the flanking segments: **Cb2**(1x in **a5** and **a6**), **Bd1a**(1x in **a5**; 0x in **a6**). Thus **R1** is retained in **a5** but truncated in subclone **a6**. Moreover, based on junctions in **a5** and **a6**, we can assemble the following segments flanking **R2**: **G1/g11(-):R2(+)** and **R2(-):U1:Cb1/Cb1a(+)**. The copy number of these flanking segments in **a5** and **a6** are **G1/g11(-)**(1x in **a5** and **a6**) and **Cb1(+)**(1x in **a6**; 2x in **a5**). Thus, the different copy-number states of **R2(+)** and **R2(-)** in subclone **a5** indicates a discontinuity of segment **R2** in subclone **a5**. Thus, we infer the structure of **R** segments in both **a5** and **a6** to be #1.

We infer the segments to have been generated by the following sequence of events (bottom figure). (i) End-joining between unreplicated dsDNA fragments, **R1/R2(-)** and **U1/U2(+)**; (ii) incomplete replication creates internal ssDNA ends in **R1/R2**; (iii) fusions of newly generated dsDNA ends **R1(+)**, **R2(+)**, **U1(-)**, **U2(-)**. Note that the internal ssDNA ends remain unligated. (iv) After another round of replication, the internal ssDNA ends are converted into dsDNA ends, generating segments **R1a**, **R1b**, **R2a**, and **R2b**. **R1b** is lost; **R1a** (final name **r1_1**) and **R2a** undergo further fusions but **R2b** remains unligated. (v) **R2b** is duplicated to create **R2b1/R2b2**; **r1_2** is derived from **R1** or **R1a**. Based on this inference, the adjacent breakpoints of **R2a(-)** and **r1_1(-)** originate from sister DNA ends generated by a simple replisome; the ancestor of **r1_1** could have been duplicated to produce **r1_2**.

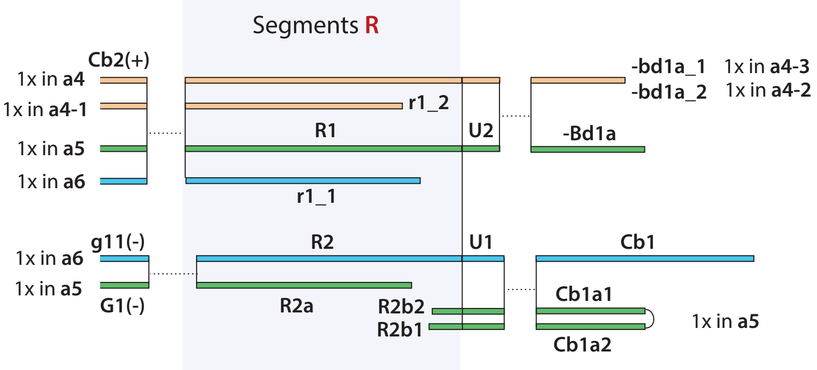

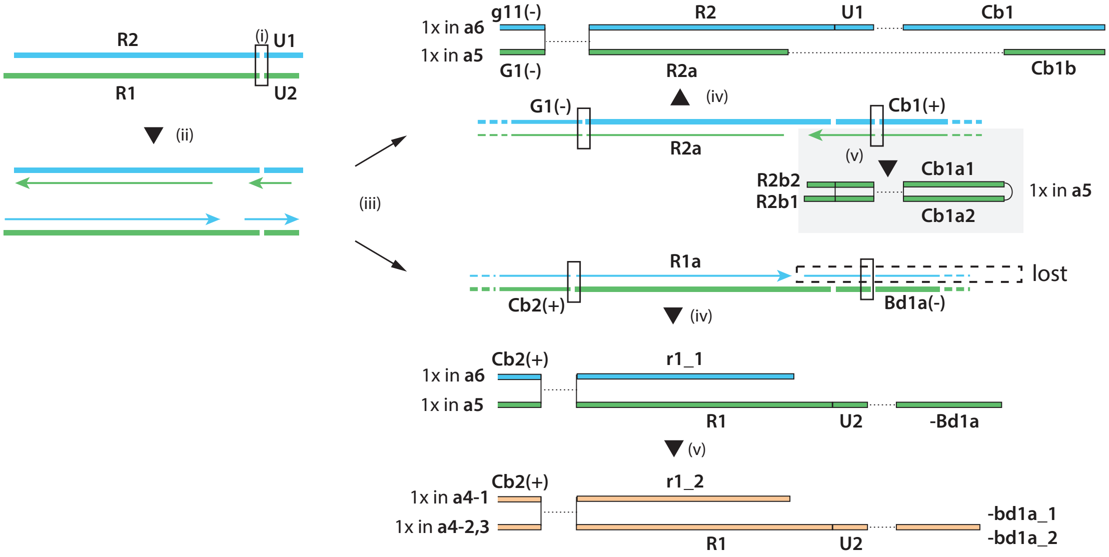

SI Figure 53: Subclonal copy-number variation in bridge clone **a** in region J:39-41Mb

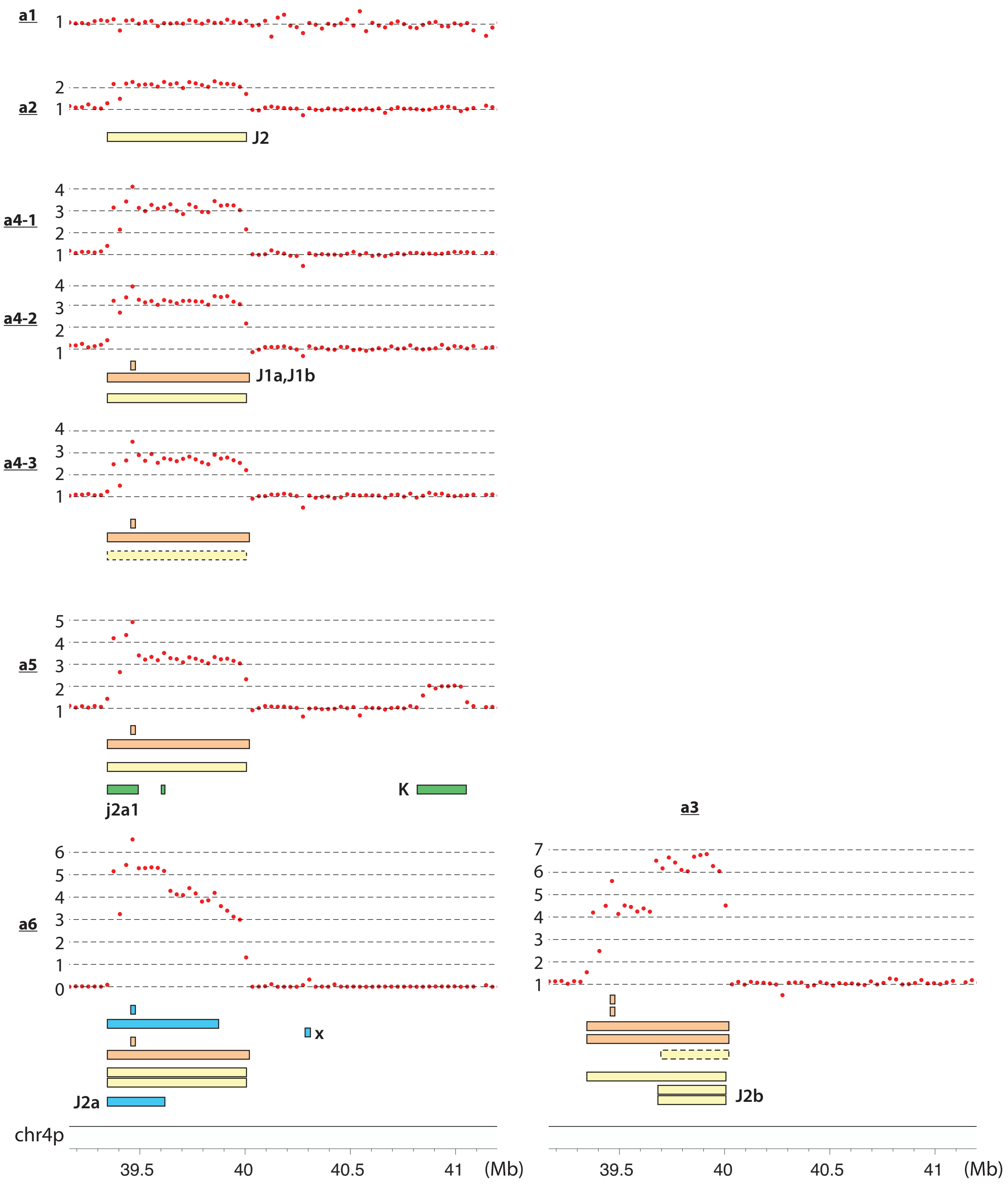

Copy-number gains at 40.83-41.05Mb in subclone **a5** and at 40.29-40.31Mb in subclone **a6** are attributed to segments **K** and **x**. Segment **x** is private to subclone **a6** but segment **K** is inferred to be ancestral as the breakpoints form junctions with segments **I1** and **M2**, both of which inferred to be ancestral. The **J** segments are discussed on the next page.

SI Figure 54: Segments in region J:39-41Mb

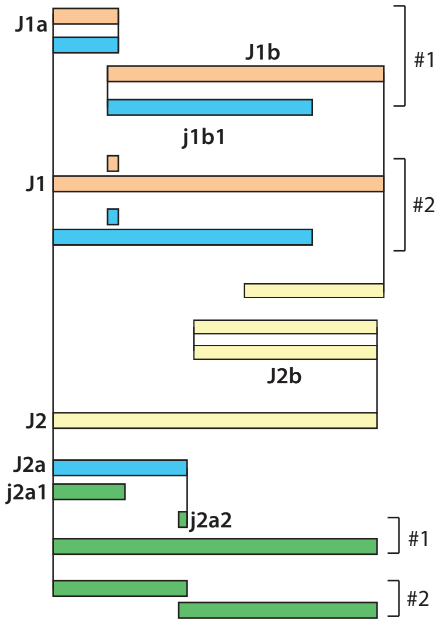

For the amplified **J** segments, the copy-number gains in subclone **a2** and **a4** suggest the presence of two sister segments **J1** and **J2** (39.35-40.01Mb), plus a 23kb triplication at 39.45-39.47Mb. We infer there is a contiguous **J2** segment based on the copy-number gain in subclone **a2**; the 23kb triplication in **a4** can be realized either with a 23kb overlap between two segments (**J1a/J1b**, #1), or with a single segment **J1** plus a 23kb segment (#2). We favor the two-piece configuration because of the opposite coordination between breakpoints that we attribute to sister-DNA exchange (cf. **Extended Data Figure 8**). However, the other configuration cannot be definitively excluded.

We further determine the presence of segment **J2a** (39.36-39.63Mb) in subclone **a6** and **j2a1** (39.36-39.48Mb) in subclone **a5**. Although the breakpoint of **j2a1(-)** at 39.482Mb is close to the breakpoint at 39.469Mb [**J1a(-)**], we infer **j2a1(-)** to be a secondary breakpoint because its partner **R2b1(+)** is secondary; by contrast, **J1a(-)** is an ancestral breakpoint. Therefore, the adjacency between **J1a(-)** and **j2a1(-)** likely occurs by chance.

Subclone **a5** further contains a small duplication at 39.622-39.630Mb; this short duplication can be realized either as a small segment **j2a2**(#1), or two partially overlapping fragments (#2) generated by replication-bypass. Before discussing these two possible configurations, we first analyze the segmental copy number to determine long-range segmental structure of rearranged DNA.

Based on breakpoint junctions, we assemble the following compound segments in the rearranged DNA (right figure):

i (from **a1**, gray) **cb11:Ca:Db2:-F2**;
ii (from **a2**, yellow) **cb11:Ca:Db2:-F1:-T2:Pa:J2**(:G2:-O:S);
iii (from **a4**, orrange) **Cb2:Ca:Db1**(:Da:-Cc1);
iv (from **a4**) (S:Cc2:-Da:N1:-M1b:Q2:Pb:L1:) **-J1a:-Pa:T1:J1b**(:Cc1);
v (from **a6**, blue) **Cb1:Ca:Db2:-F1:-T2:Pa:J2a**(:E:H1);

The bold-faced segments are shown on the right and the flanking segments (in parentheses) are not shown.

From the compound segments we can then infer the structure of the ancestral DNA fragments. From (i) and (ii), we infer an ancestral DNA with the same composition as (ii). Based on adjacent breakpoints on **J1/J2**, we infer that (ii) and (iv) are derived from opposite strands of an ancestral dsDNA fragment. Note the opposite strand coordination between **T1/T2** and **J1/J2** due to sister-chromatid exchange. Based on adjacent breakpoints on **Cb1/Cb2** and **Db1/Db2**, we infer (iii) and (v) are derived from opposite strands of another ancestral dsDNA fragment.

We can further invoke re-replication due to late-firing origins within **F1** to explain the triplication consisting of **cb11**(277kb), **Ca**(31kb), **Db2**(59kb), **-F1**(108kb), **-T2**(77kb), **-Pa**(253kb), and **J2a**(272kb). The total size of re-replicated DNA (1.08Mb) is approaching the maximum size of a single replicon.

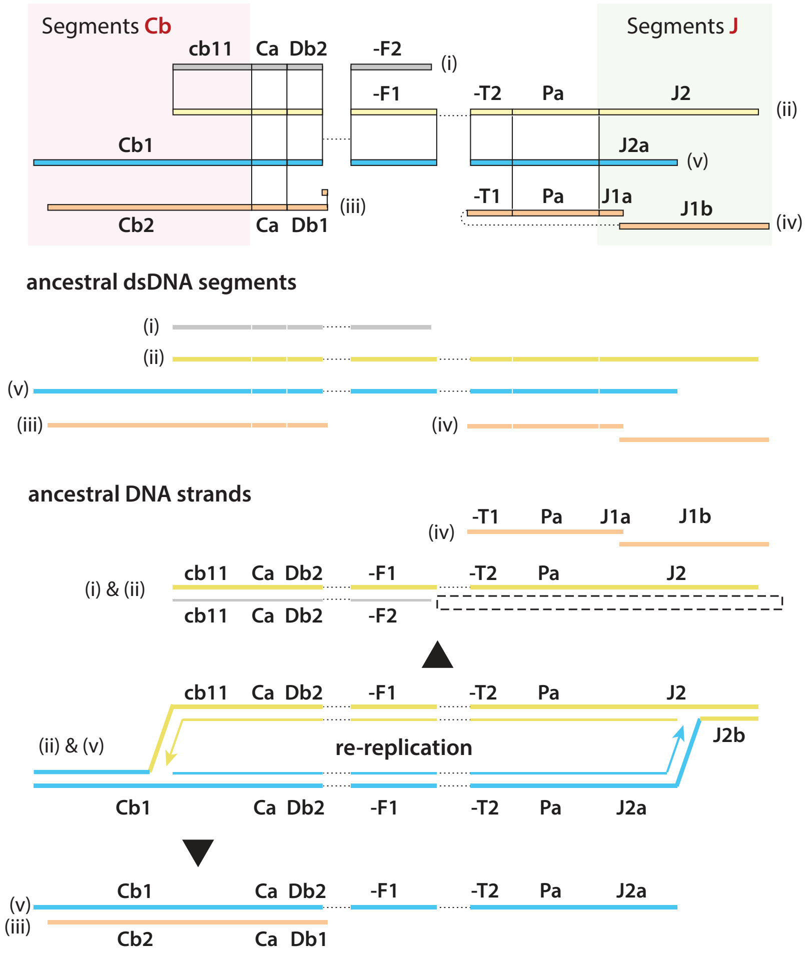

Based on the assembled chromosome fragments (ii-v), we can determine the copy number state of each compound segment in each subclone as shown below. This analysis can both verify the continuity of the assembly and determine the locations of secondary breakpoints on duplicated segments. For example, the presence of a single copy of **Cb1** and **Cb2** in subclone **a6** indicates there is a single copy of **J2a** (linked to **Cb1**) and **J1b** (linked to **Cb2**); but the presence of two copies of **S** that are linked to **J1a** indicates a discontinuity between **J1a**(+) and **J1b**(-) in subclone **a6**. This leads to the inference of **j1b1**. Note that this inference is independent of whether there is a single **J1** segment or two segments **J1a/J1b**.

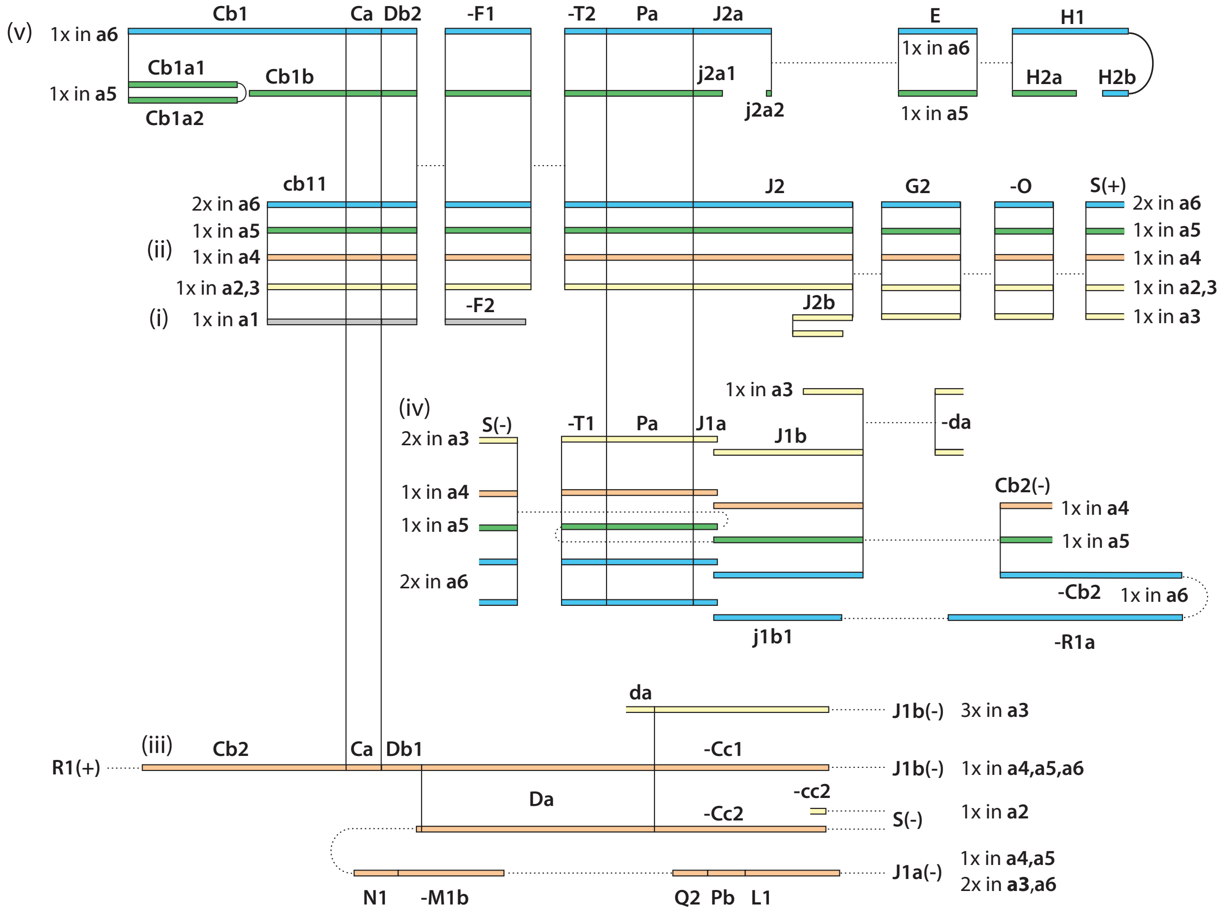

There remains some uncertainty about amplified segments in subclone **a3** and **a5**. In **a3**, there are three extra truncated copies of **J1/J2** and we can only identify one breakpoint at 36.66Mb(+) that is fused with telomeric repeats. A plausible origin of this breakpoint is from the breakage of the re-replication bubble. We therefore named this segment **J2b** (36.66-40.01Mb) as a *cis* segment of **J2a** (39.35-39.62Mb), although we cannot rule out the possibility that they are generated independently. In **a5**, the private breakpoints at 39.48Mb and 39.63Mb can reside on any of **J2a**, **J2**, or **J1b**, as shown on the right. We choose the first configuration in the schematic diagram for convenience. In either configuration, the breakpoint at 39.63Mb with newly added telomere repeats represents the end of the rearranged chromosome.

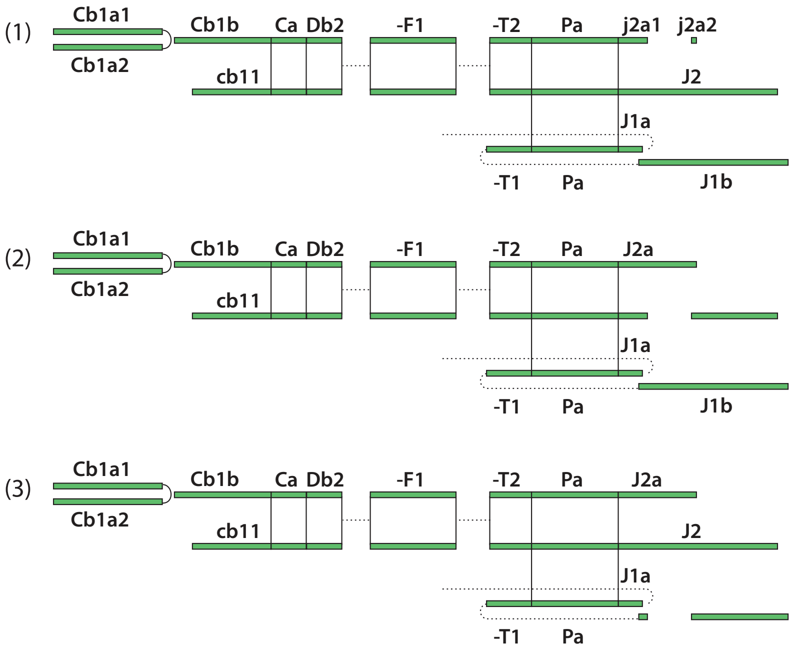

11. Segmental structure of rearranged chromosomes in different subclones of bridge clone a

In the previous section, we present data related to the determination of the breakpoints of all the segments in bridge clone **a**. Here we show how these data are used to determine the structure of the rearranged chromosome as shown in **Extended Data Figure 5**.

Rearranged chr4 in subclones a1, a2, a4. We start with the rearranged chromosomes in subclone **a1**, **a2**, and **a4**. In subclone **a1** and **a2**, all but one junction are unique. The only duplicated junction is the **L2/l21:-V1/V2** junction (highlighted in gray); however, this ambiguity only affects the order of **V1/V2** segments, but not of the other segments. We can therefore directly determine the order of segments linked by unique junctions: **A→L2:-V2:V1:-l21**· · · ① and ❶· · · **cb11→-F1/F2**· · · ②. ① and ❶ denote a connection between segments; similar for ② and ❷.

For segments in subclone **a4**, the following junctions are duplicated (highlighted in gray): **Q1:Pb:L**, **Cb2/cb11:Ca:Db**, **Da:-Cc**, and **-T:Pa:J**. Based on the structure of rearranged chr4 in subclone **a1** and **a2**, we can phase the junctions in **Q:Pb:L**, **Cb2/cb11:Ca:Db**, and **-T:Pa:J** segments, leaving the **Da:-Cc1/Cc2** junctions as the only ambiguity. Based on the phasing of junctions in **-T:Pa:J**, we determine the structure of fragment ⑤→❹. The ambiguous phasing of the **Da:-Cc1/Cc2** junctions implies two possible configurations: One produces ④→❸ as shown in the figure below, the other produces ④→❺ with a crossover within **Da:Cc**. As the second configuration results in an acentric circle ④→❺ · · · ⑤→❹ that is inconsistent with the stable copy-number states of these segments, we can rule it out.

The segmental structure of rearranged chr4 in subclones **a1**, **a2**, and **a4** are shown below. For better illustration of the phasing of duplicated segments, segments are colored based on the subclone where they are present at the lowest copy-number state (usually single copy): **a1** (gray); **a2** (yellow); **a4** (orange). Open and filled numbers denote connections between segments. Duplicated segments are highlighted in gray rectangular areas. A triplicated segment (**l21**) is highlighted in a red area.

SI Figure 55: Segmental structure of rearranged chr4 in subclones **a1**, **a2** and **a4**

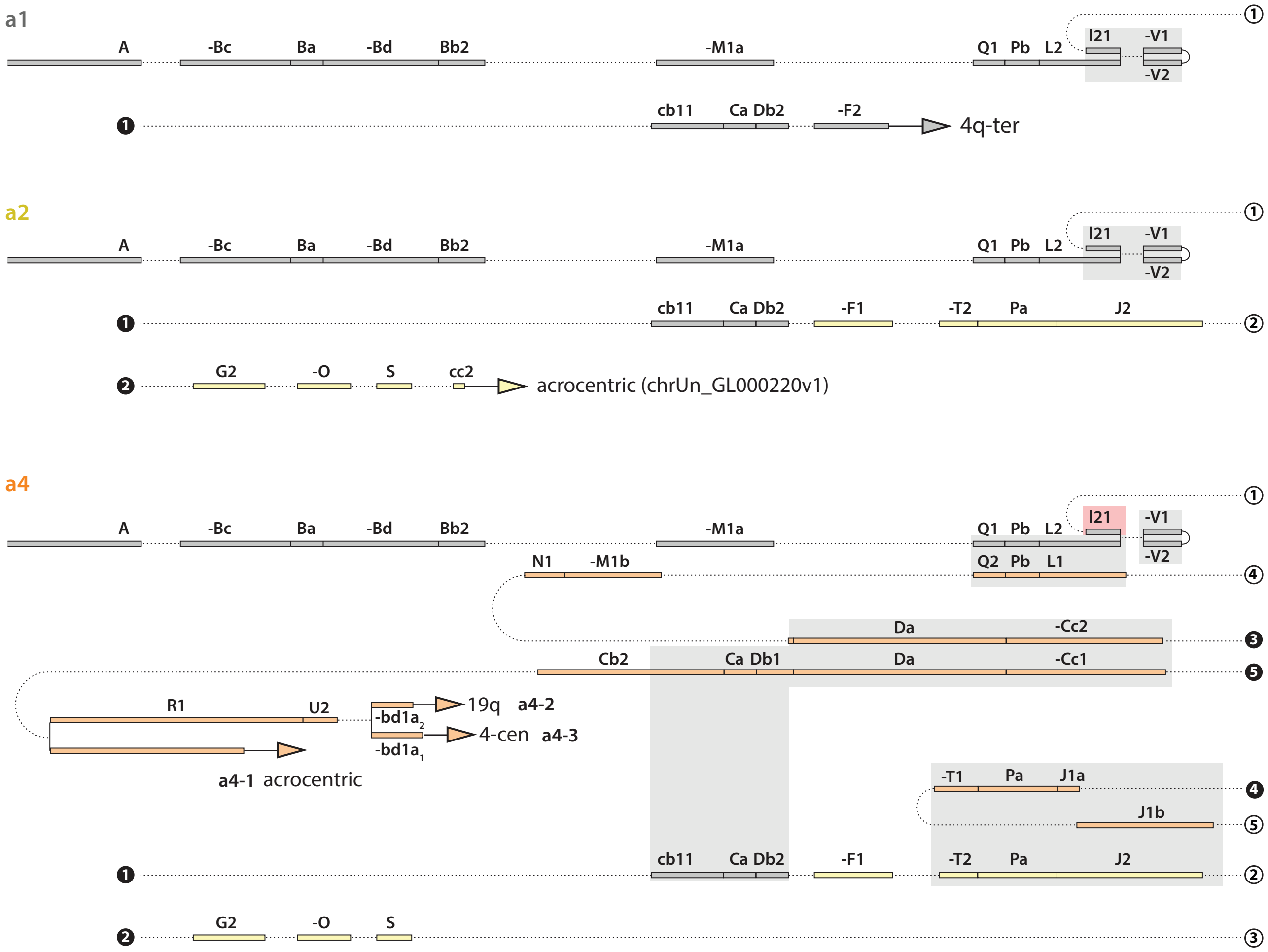

The end of the rearranged chr4 in subclone **a1** is capped by a telomeric segment from 4q (gray triangle). The end of rearranged chr4 in subclone **a2** is capped by rDNA (`chrUn_GL000220v1`). The end of rearranged chr4 in **a4-1** is capped by a sequence that maps to acrocentric arms; the end of rearranged chr4 in **a4-2** is capped by a telomeric segment from 19q; the end of rearranged chr4 in **a4-3** is capped by a sequence that maps to the chr4 centromere. The approximate locations of the repeats are determined by alignment to the `CHM13` reference using `BLAT` from the UCSC genome browser.

Rearranged chr4 in subclone a3. We can partially determine the structure of amplified DNA in subclone **a3** based on the order of segments determined in subclones **a2** and **a4** and the segmental copy number in subclone **a3**.

In **SI Figure 56**, each solid line represents a single copy and dashed lines represent a partial copy indicating subclonal loss. The yellow circle below segment **J2** denotes a fusion to telomeric repeats (the truncated segment is denoted **J2b**); the yellow triangle next to segment **da1** denotes a fusion to rDNA repeats; the copy number of these two fusions are undetermined.

Based on the segmental copy number, we infer there is an extra copy of the chromosome fragment from **J2b** to **Cc1** (②➔❺) and an extra copy of two partial duplications of **J2:G2** and **J1b:Cc1**. The linkage between these duplications and the end of rearranged chr4 in subclone **a3** cannot be determined.

SI Figure 56: Segmental structure of rearranged chr4 in subclone **a3**

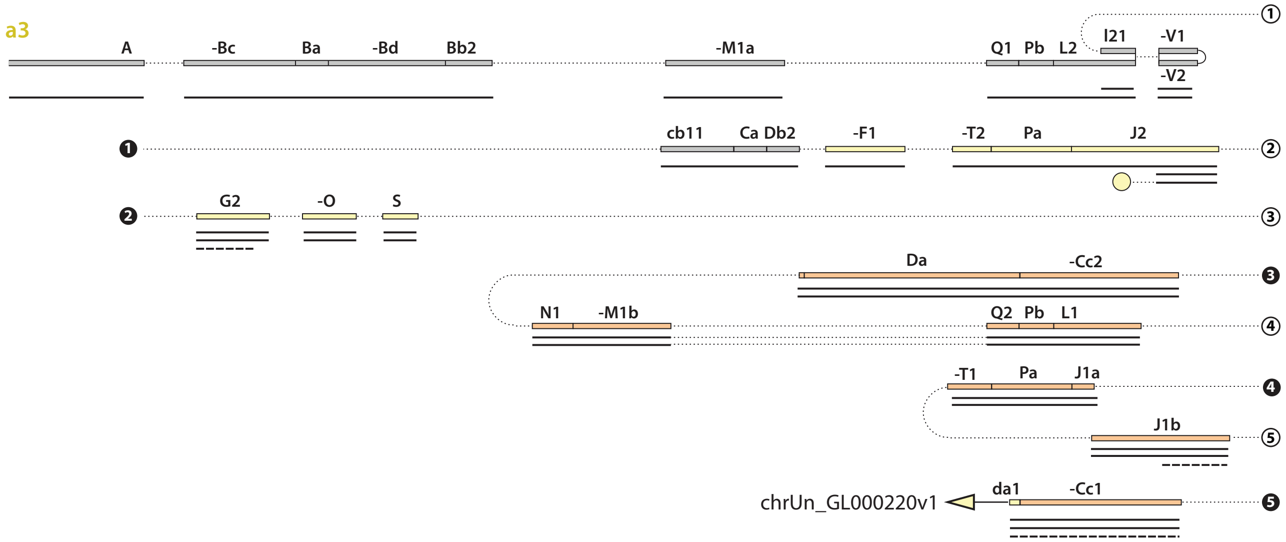

Rearranged chr4 in subclone a5 and a6. Subclones **a5** and **a6** contain additional duplications including both ancestral segments and secondary duplications of ancestral segments. We assemble two additional fragments of the ancestral rearranged chromosome: ❻→⑦ assembled from segments in subclone **a5** and ❼→⑧ assembled from junctions preserved in both **a5** and **a6** (bottom box). A comparison between **a5** and **a6** suggests an uneven segregation of ❼→❽ (bottom box). This uneven segregation most likely occurred in the next generation after the formation of the ancestral rearranged chromosome.

The structure of rearranged chr4 in subclone **a5** is determined completely, with the chromosome end mapped to **j2a2** that is capped by telomeric repeats (green triangle). The structure of rearranged chr4 in subclone **a6** is not completely resolved.

SI Figure 57: Segmental structure of rearranged chr4 in subclones **a5** and **a6**

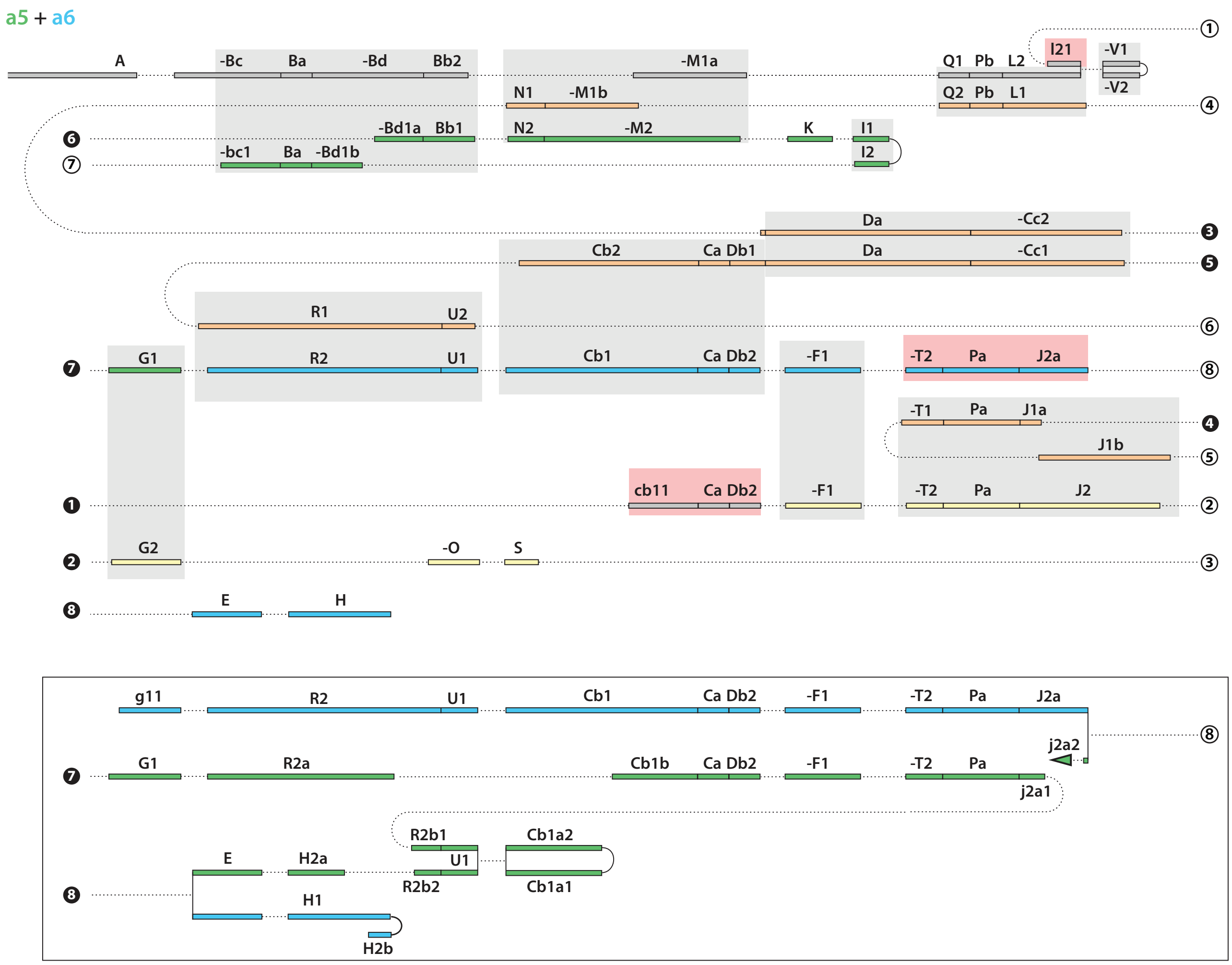

Structure of the ancestral rearranged chr4. We infer the structure of the *ancestral* rearranged chr4 (**SI Figure 58**) based on the synteny of segments preserved in all the subclones. The ancestral DNA segments are colored by the same scheme as above. Features of the junctions between segments are represented as follows. Solid lines: no insertion or short insertions that cannot be mapped; long dashed lines: one insertion; red short dashed lines: complex junctions with more than one insertion. Complex junctions are annotated (**c01-c13**,**c15**). Complex junction **c14** is between **H1** and **H2b** (**SI Figure 57**) and is likely generated in the next generation.

SI Figure 58: Segmental structure of the ancestral chr4 in bridge clone **a**.

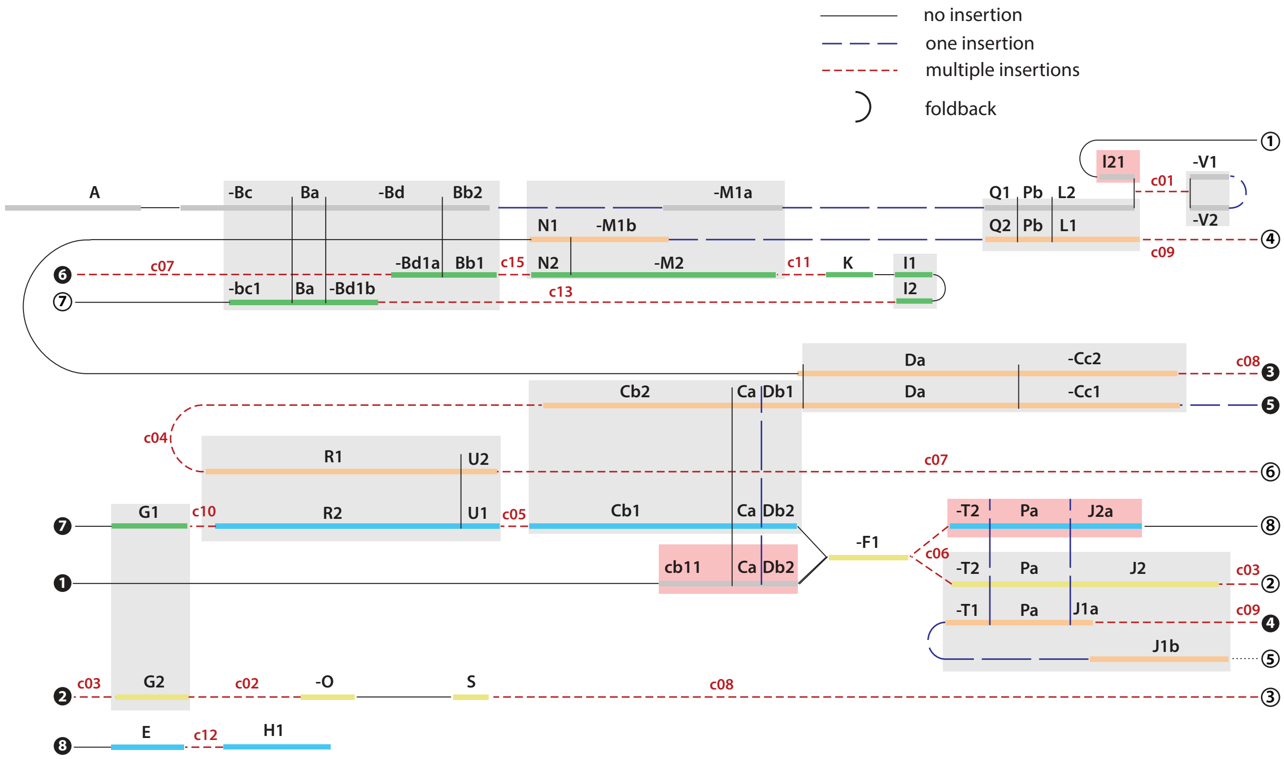

Based on the structure of the ancestral DNA, we can draw the following conclusions.

(1) The rearranged chromosome start with segment **A** at the p-terminus and ends at segment **H**.

(2) The following segments are not duplicated: **A**,**E**,**H**,**K**,**O**,**S**. We infer segments **H2a/H2b** to be secondary duplications since the right breakpoint of **H2a** joins secondary breakpoints on **R2b2**.

(3) The following segments are triplicated (red rectangular areas): **cb11, I21, J2a**.

(4) All the remaining segments (gray rectangular areas) are generated by replication of non-overlapping dsDNA fragments in a single breakage-replication/fusion cycle. Except for **bc1** as a partial duplication of **Bc**, all the remaining duplications are paired as replicated sister DNA segments.

(5) Foldback junctions between **V1/V2** and between **I1/I2** are within the rearranged chromosome (internal foldbacks) but not at chromosome ends.

(6) As sister duplications are generated concurrently, the absence of one sister segment in a subclone (e.g., **Q2** is not present in **a1**) indicates a secondary loss. It is noteworthy that many of the secondary breakpoints are close to breakpoints inferred to be ancestral DNA ends, e.g., **F1(+)** in **a1**, **Cc2(+)** in **a2**, **Da(-)** in **a3**, **J2a(-)** in **a5**. A plausible explanation is that these secondary breakpoints are derived from single-strand nicks/gaps in the ancestral rearranged chromosome that are converted to DSB ends by replication in subsequent generations (see **SI Figure 12** for a plausible explanation).

Additional segments in a6 from a secondary chromothripsis. The copy number of rearranged chr4 in subclone **a6** (**SI Figure 59**) indicates a near complete duplication of fragments ②-⑤ and duplication/secondary fragmentation of fragment ①.

SI Figure 59: Copy-number of chr4 segments in subclone **a6**

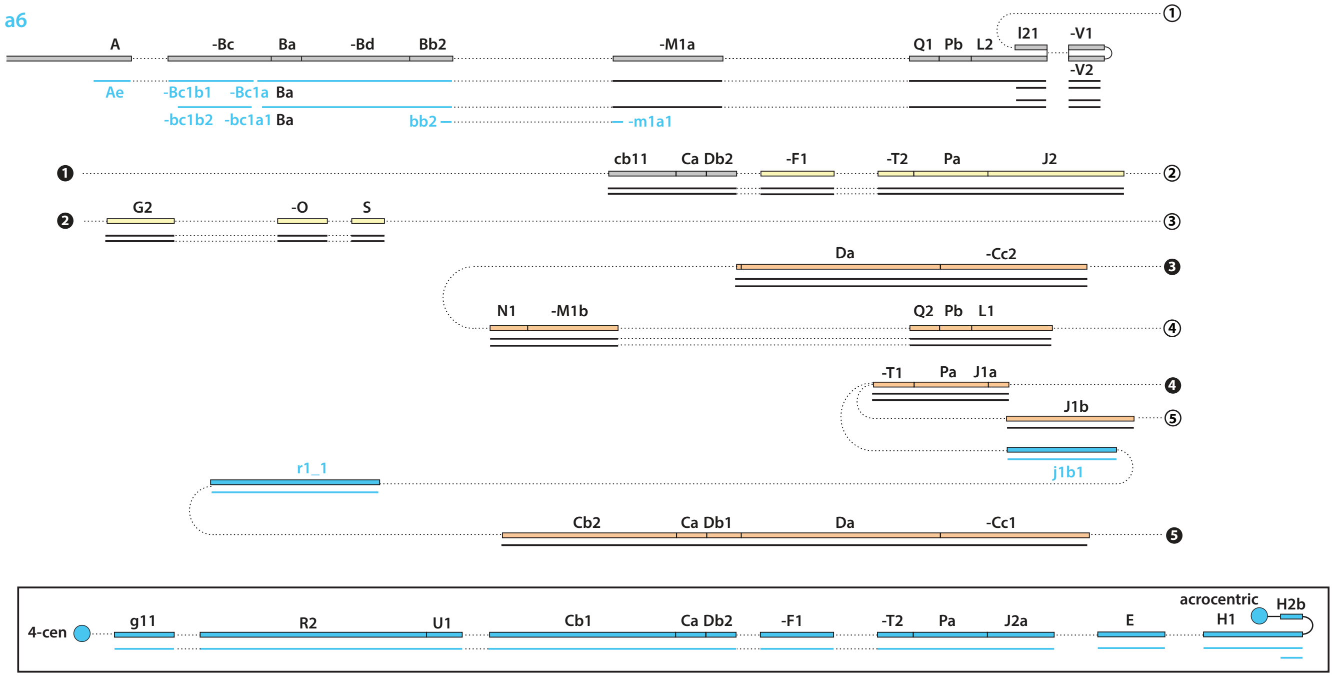

We further infer that the secondary chromothripsis in **a6** involves both chr4 and chr14q and creates complex segmental gains on chr14q (figure on next page). The breakpoints and junctions on chr14q are listed in **Supplementary Table 19**. The concurrence of these events is supported by two observations. First, there is a junction between segment **Bc1b1** and a breakpoint on chr14:20049947(+). Second, the junction between **j1b1** and **r1_1** contains a short insertion that is mapped to a region on chr14 (20049701-938) right next to this breakpoint. Note that two adjacent gapped breakpoints (19,894,715 and 19,901,428) representing a short deletion join distal breakpoints on gained segments. Therefore, the chromothripsis on chr14 also shows three copy-number states (instead of two), consistent with breakage-replication/fusion.

SI Figure 60: Secondary chromothripsis of chr4 and chr14 in subclone **a6**

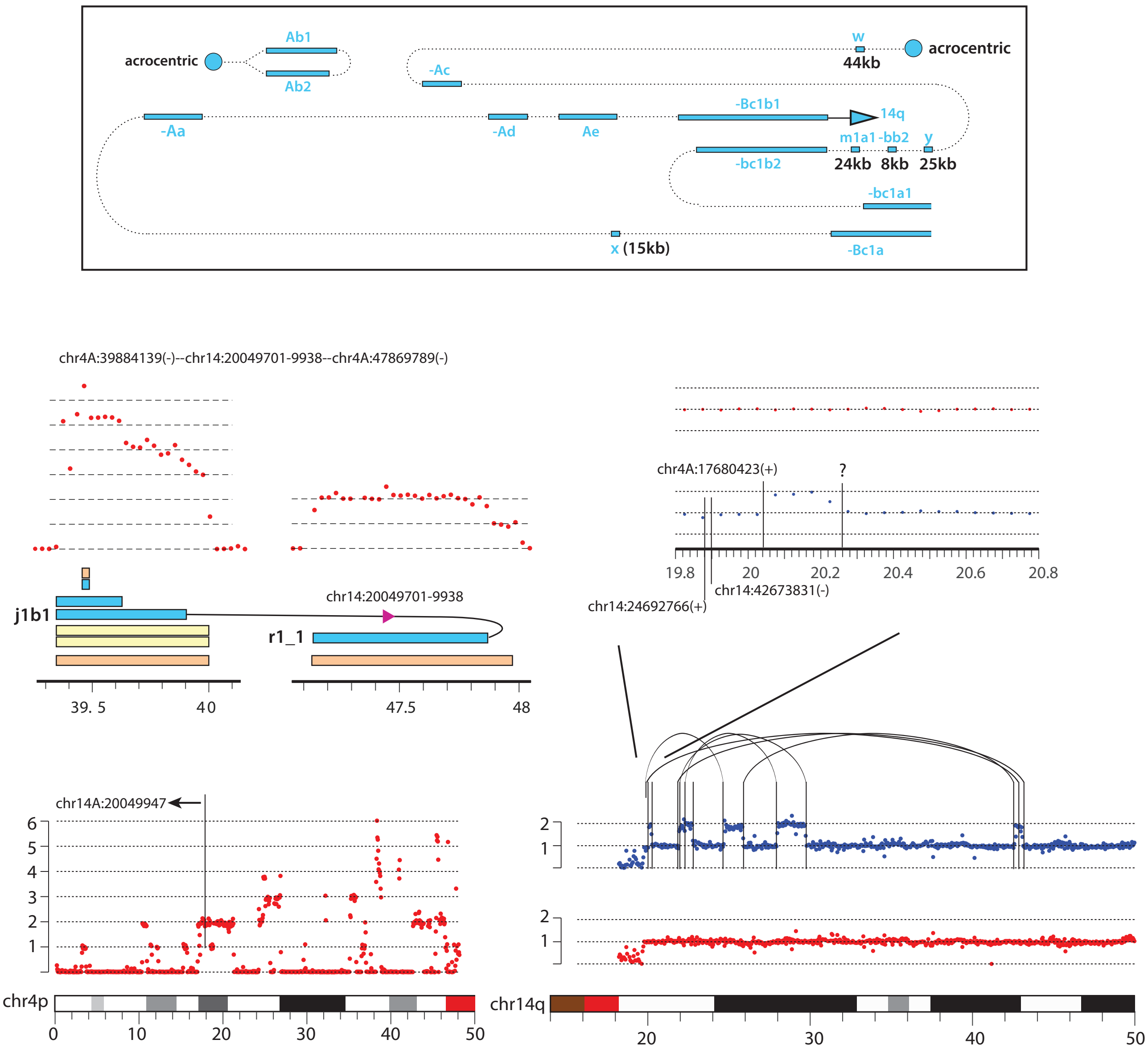

12. Foldback junctions in all the subclones of bridge clone a

The joining of adjacent parallel breakpoints on sister segments creates a foldback junction. Foldback junctions can arise from two processes (**SI Figure 61**). The first is by replication through a hairpin derived from a staggered DSB end (**SI Figure 61**, top), as demonstrated in a recent study in yeast (1). This mechanism is a breakage-fusion-**replication** sequence. Foldback junctions can also result from the ligation of replicated sister DNA ends in a breakage-**replication**-fusion sequence (**SI Figure 61**, bottom).

SI Figure 61: Foldback junctions on both chr4 homologs in the subclones of bridge clone **a**

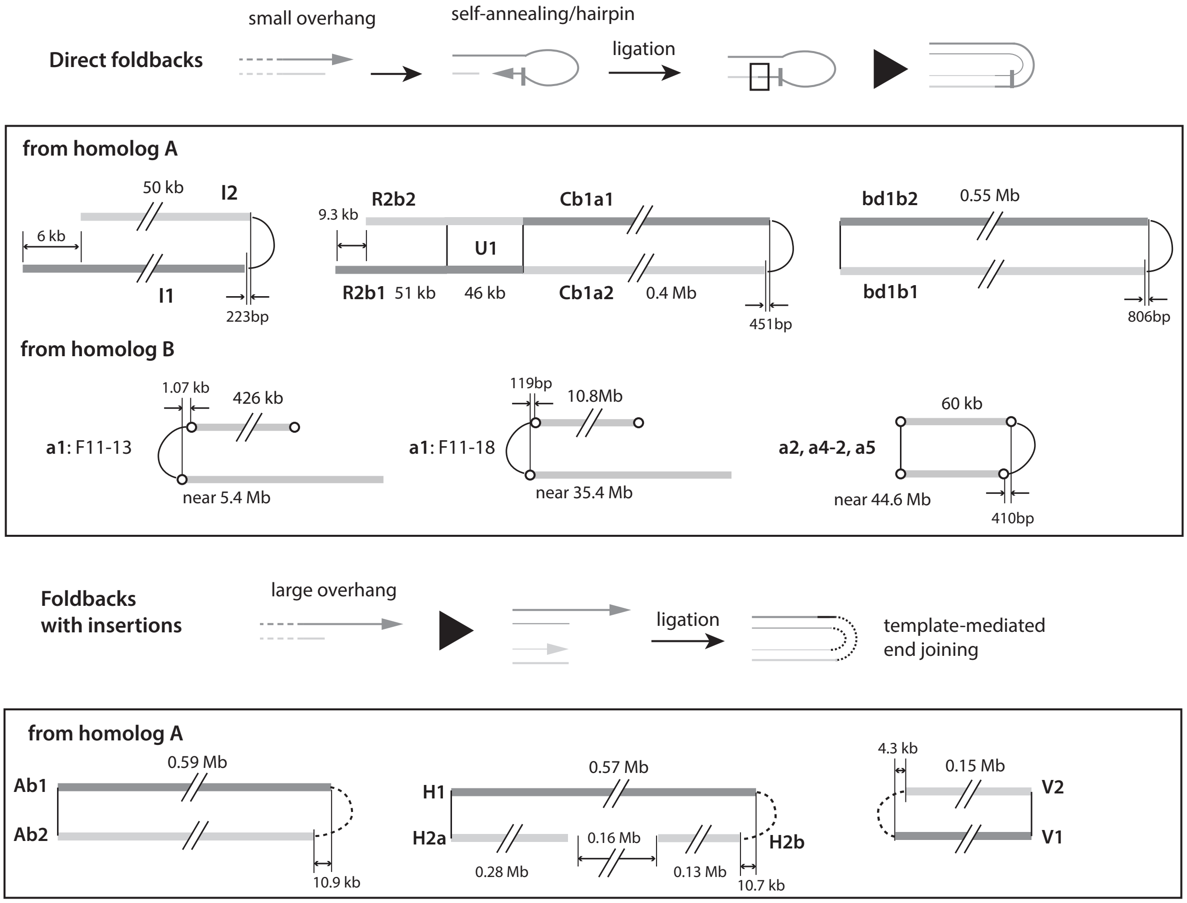

We identified a total of 9 foldback junctions from all the subclones of bridge clone **a**: six from the fragmented A homolog, three from the B homolog (**Supplementary Table 18**). Six junctions were direct foldbacks and the distance between breakpoints in each junction was within 1kb (**SI Figure 61**, top). Three foldback junctions contained insertions (**SI Figure 61**, bottom) and the breakpoints were further apart (4.3kb, 10.7kb, and 10.9kb). These observations are consistent with the two mechanisms of foldbacks from breakage-replication/fusion. In particular, we suggest that foldback junctions joining breakpoints that are further apart are generated by breakage-**replication**-fusion and can accept insertions by a similar repair mechanism that creates long-range junctions with insertions.

SI Figure 62: DNA segments of homolog 4B in subclones **a2**, **a4**, and **a5** near 44.6Mb

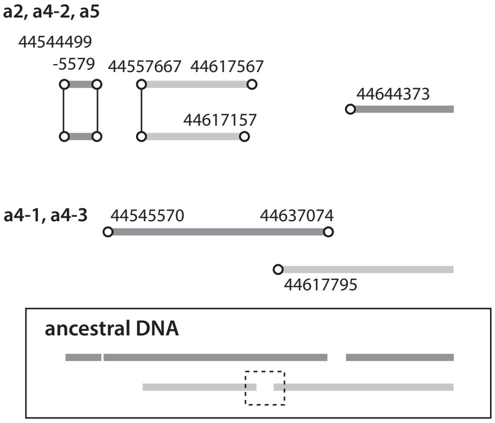

For foldback junctions at chromosome ends, including those generated by bridge breakage, we expect to see both deletion of DNA on the telomeric side of the foldback breakpoints and a long-range palindromic structure in the rearranged DNA. This was observed for the two foldback junctions on homolog B in two subclones of subclone **a1**. By contrast, foldback junctions between sister DNA segments are not

subject to these constraints. Based on the long-range structure of the ancestral rearranged chr4A, we inferred that at least three foldback junctions (**I1/I2**, right; **Cb1a1/Cb1a2**, right; **V1/V2**, left) were formed between sister DNA segments instead of sister chromatids. For the **I1/I2** and the **Cb1a1/Cb1a2** segments, the inference that the foldback junctions arose from sister-DNA fusion can also be drawn based on the presence of staggered breakpoints on the opposite side. Finally, for the foldback junction on homolog B near 44.6Mb in subclones **a2**, **a4-2**, and **a5**, we inferred that the adjacent breakpoints were derived from an ancestral DNA end opposite to another DNA end that became the breakpoint at 44617795 in subclone **a4-1** and **a4-3** (**SI Figure 62**). (This last observation provides an interesting example of adjacent overlapping breakpoints that are segregated into different cells.)

13. Adjacent overlapping breakpoints in chromothripsis in the K-562 genome

From the long-read data of K-562 cells, we identified three pairs of adjacent overlapping breakpoints on chr18 (**SI Figure 63**). These breakpoints form translocations between each other and with two adjacent gapped breakpoints (3). The presence of adjacent overlapping breakpoints, gapped breakpoints, and parallel breakpoints (two foldback junctions) on this chromosome indicates chromosome breakage-replication/fusion.

SI Figure 63: Adjacent overlapping breakpoints on chr18 of K-562 cells

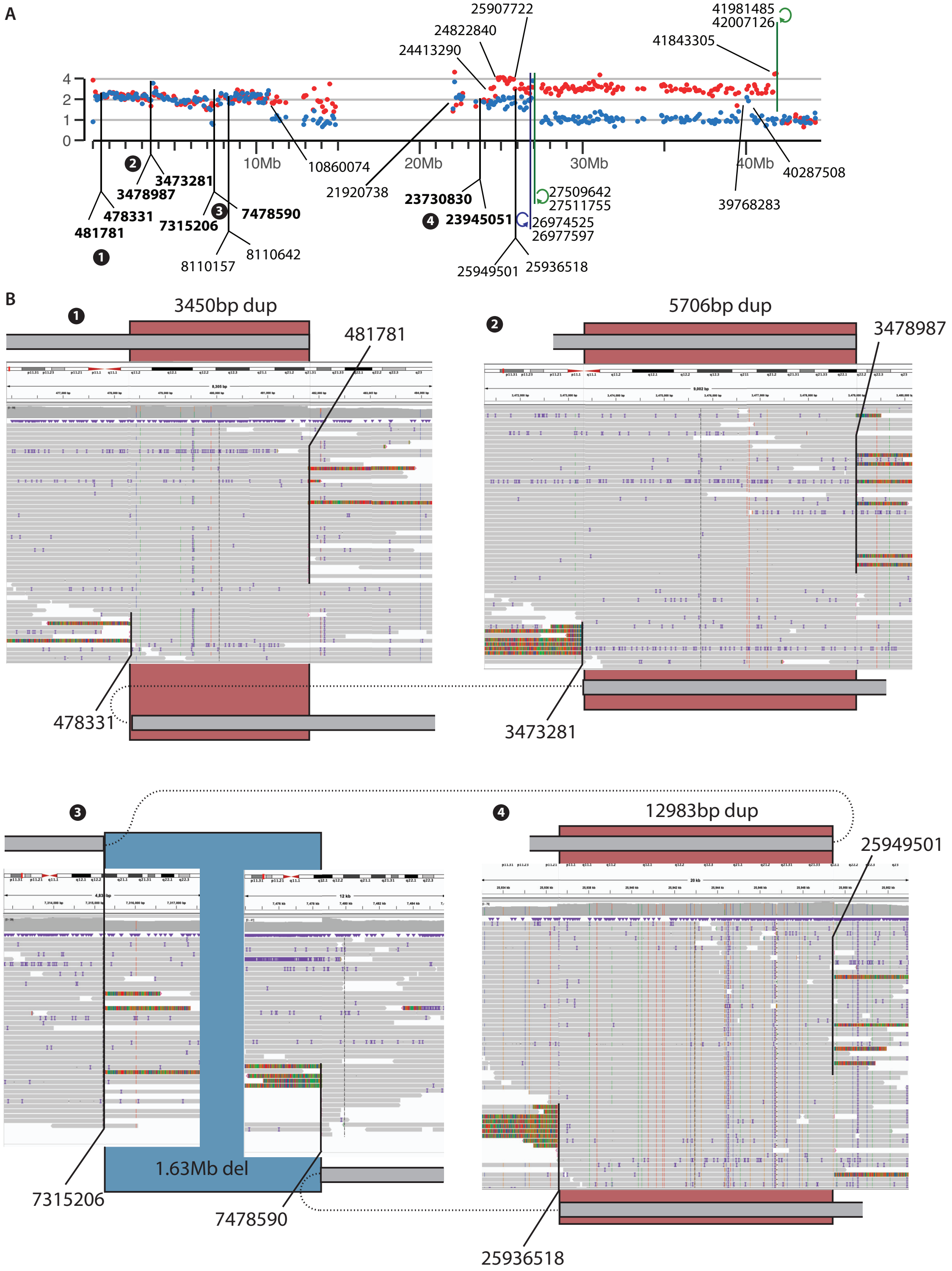

14. Insertions in single cells after bridge resolution

We clustered insertions in four single daughter cells with broken bridge chromosomes: two daughters that share a bridge (**C-4a** and **C-4b**; **SI Figure 65**), and two single daughter cells **C-2a** (**SI Figure 64**) and **T-1a** (**SI Figure 66**). [The names are according to the original names in Umbreit et al. (36).] In the **T-1a** cell, we also assembled the 8 chains of insertions (A-H), including a junction (B) containing 15 insertions (**SI Figure 64**). Notably, for the G junction, we identified two separate chains (G1-G4 and G1'-G3-G4) that are similar to the example in the HCC1954 genome (**SI Figure 29B**). These examples demonstrate that multiple insertion junctions can be generated in a single cell cycle.

In all four cells, the insertion hotspots are near the sites of chromosome breakage; in the **C-4** pair, we even identified two insertion hotspots on opposite sides of the broken chr18 (between 14.11 and 14.15Mb). By contrast, we did not observe insertion junctions in cells with unbroken bridges. Thus, insertions originate from sites of DNA breakage. Interestingly, in the **C-2a** daughter cell, we identified hotspots of insertions both near the breakage sites of bridge chromosomes (inferred based on the reciprocal copy-number gain/loss pattern between the daughter cells) and at several non-bridge chromosomes. Presumably, these non-bridge chromosome hotspots reflect sites of sporadic DNA breakage in this cell.

Based on the timing of bridge breakage after mitosis, we conclude that the initiation of DNA replication prior to bridge breakage is not required for the generation of junctions with tandem insertions. For the daughter cell pair **C-4**, bridge breakage occurred 3.5 hours after mitosis and cells were collected 24 hours after mitosis (by this timepoint the junctions were already formed). For cell **C2(a)**, bridge breakage occurred 3.5 hours after mitosis and the cell was collected 16 hours after mitosis. For cell **T-1(a)**, bridge breakage occurred 12.3 hours after mitosis and cells were collected 22.6 hours after mitosis. In all cases, the daughter cells are expected to be in G1 or early S phase when the bridge chromosomes were broken.

SI Figure 64: Insertions in single cell C-2a

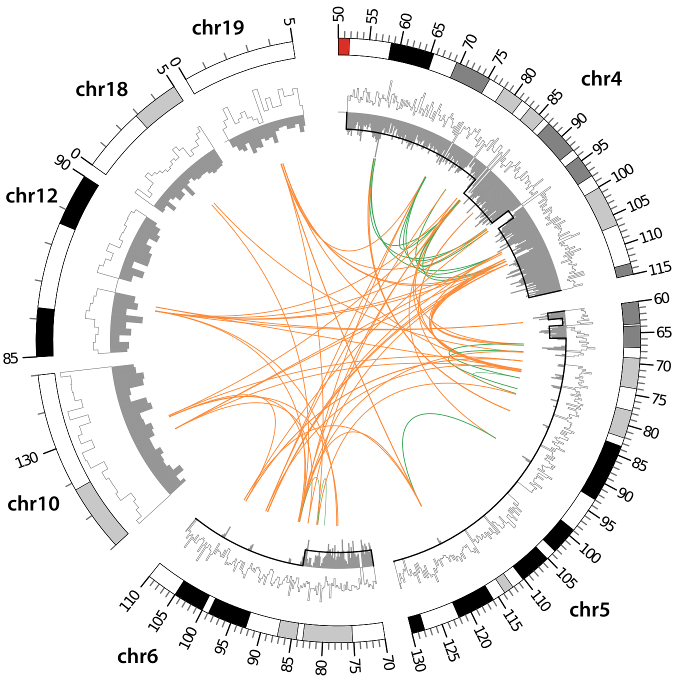

Hotspot of insertions near chr12:86.696-86.701Mb

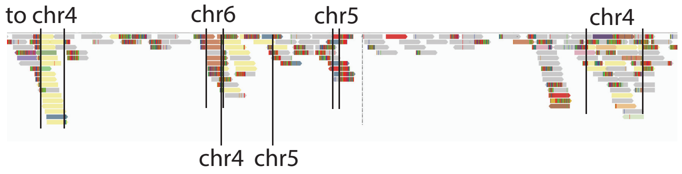

SI Figure 65: Clustering of insertions in a daughter cell pair C-4 from Umbreit et al. (36)

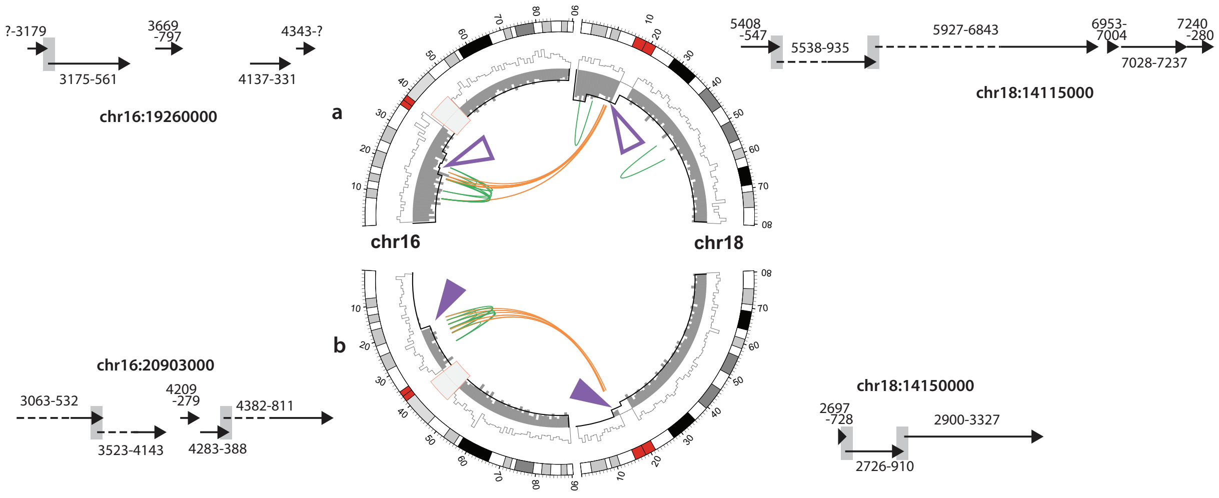

We did not observe insertion junctions in cells that were collected right after the bridge was mechanically broken (36). We also did not observe insertion junctions in cells with micronuclei, although insertion junctions were prevalent in daughter cells that inherited damaged chromosome from micronuclei (36, 40). Therefore, DNA breakage is required but insufficient to generate insertions.

SI Figure 66: Clustering of insertions in single cell T-1a

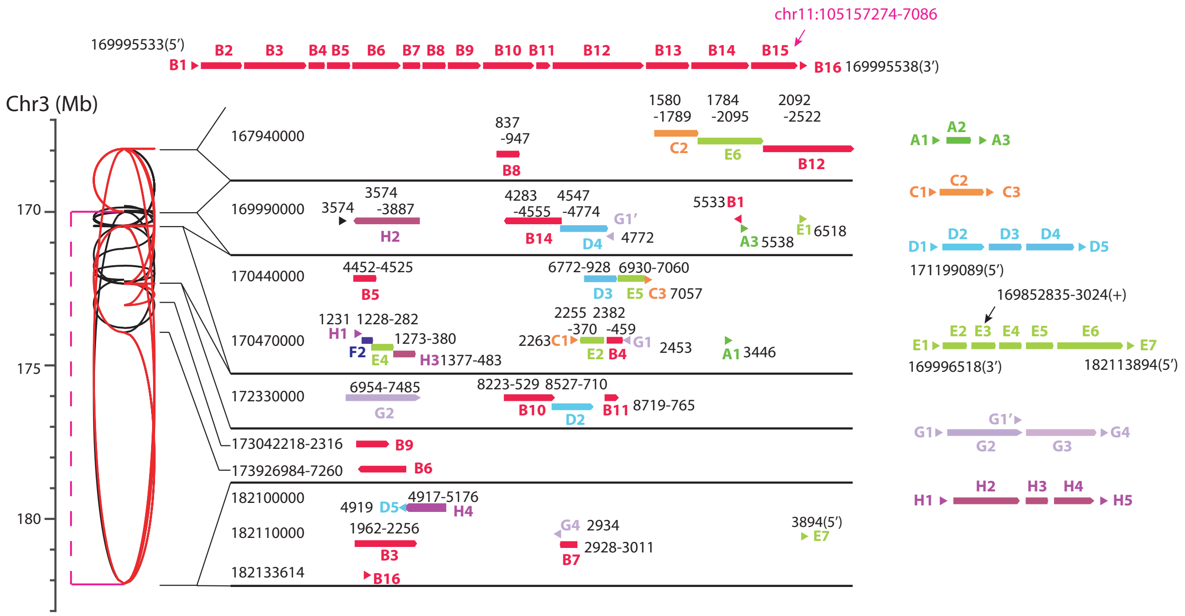

References

1. Al-Zain, A.M., M.R. Nester, I. Ahmed, and L.S. Symington. 2023. Double-strand breaks induce inverted duplication chromosome rearrangements by a dna polymerase delta-dependent mechanism. Nat Commun 14(1): 7020. doi:10.1038/s41467-023-42640-5 .
2. Alexander, J.L., M.I. Barrasa, and T.L. Orr-Weaver. 2015. Replication fork progression during re-replication requires the dna damage checkpoint and double-strand break repair. Curr Biol 25(12): 1654–60. doi:10.1016/j.cub.2015.04.058 .
3. Anand, R.P., S.T. Lovett, and J.E. Haber. 2013. Break-induced dna replication. Cold Spring Harb Perspect Biol 5(12): a010397. doi:10.1101/cshperspect.a010397 .
4. Baris, Y., M.R.G. Taylor, V. Aria, and J.T.P. Yeeles. 2022, May. Fast and efficient dna replication with purified human proteins. Nature 606(7912): 204–210. doi:10.1038/s41586-022-04759-1 .
5. Bhattacharjee, A., Y. Wang, J. Diao, and C.M. Price. 2017, 10. Dynamic dna binding, junction recognition and g4 melting activity underlie the telomeric and genome-wide roles of human cst. Nucleic Acids Research 45(21): 12311–12324. doi:10.1093/nar/gkx878. https://academic.oup.com/nar/article-pdf/45/21/12311/22146311/gkx878.pdf .
6. Brunette, G.J., R.W. Tourdot, D. Zong, D. Pellman, and C.Z. Zhang. 2024. Haplotype-resolved karyotype construction from hi-c data using reflinker. bioRxiv: 2024.03.02.583108. doi:10.1101/2024.03.02.583108 .
7. Costantino, L., S.K. Sotiriou, J.K. Rantala, S. Magin, E. Mladenov, T. Helleday, J.E. Haber, G. Iliakis, O.P. Kallioniemi, and T.D. Halazonetis. 2014. Break-induced replication repair of damaged forks induces genomic duplications in human cells. Science 343(6166): 88–91. doi:10.1126/science.1243211 .
8. Dilley, R.L., P. Verma, N.W. Cho, H.D. Winters, A.R. Wondisford, and R.A. Greenberg. 2016. Break-induced telomere synthesis underlies alternative telomere maintenance. Nature 539(7627): 54–58. doi:10.1038/nature20099 .
9. Donnianni, R.A. and L.S. Symington. 2013, July. Break-induced replication occurs by conservative dna synthesis. Proceedings of the National Academy of Sciences 110(33): 13475–13480. doi:10.1073/pnas.1309800110 .
10. Donnianni, R.A., Z.X. Zhou, S.A. Lujan, A. Al-Zain, V. Garcia, E. Glancy, A.B. Burkholder, T.A. Kunkel, and L.S. Symington. 2019. Dna polymerase delta synthesizes both strands during break-induced replication. Molecular Cell 76(3): 371–381.e4. doi:https://doi.org/10.1016/j.molcel.2019.07.033 .
11. Hastings, P.J., J.R. Lupski, S.M. Rosenberg, and G. Ira. 2009. Mechanisms of change in gene copy number. Nat Rev Genet 10(8): 551–64. doi:10.1038/nrg2593 .
12. Kinsella, M., A. Patel, and V. Bafna. 2014. The elusive evidence for chromothripsis. Nucleic Acids Res 42(13): 8231–42. doi:10.1093/nar/gku525 .
13. Kockler, Z.W., B. Osia, R. Lee, K. Musmaker, and A. Malkova. 2021. Repair of dna breaks by break-induced replication. Annu Rev Biochem 90: 165–191. doi:10.1146/annurev-biochem-081420-095551 .
14. Li, Y., N.D. Roberts, J.A. Wala, O. Shapira, S.E. Schumacher, K. Kumar, E. Khurana, S. Waszak, J.O. Korbel, J.E. Haber, M. Imielinski, P.S.V.W. Group, J. Weischenfeldt, R. Beroukhim, P.J. Campbell, and P. Consortium. 2020. Patterns of somatic structural variation in human cancer genomes. Nature 578(7793): 112–121. doi:10.1038/s41586-019-1913-9 .
15. Li, Y., C. Schwab, S.L. Ryan, E. Papaemmanuil, H.M. Robinson, P. Jacobs, A.V. Moorman, S. Dyer, J. Borrow, M. Griffiths, N.A. Heerema, A.J. Carroll, P. Talley, N. Bown, N. Telford, F.M. Ross, L. Gaunt, R.J. McNally, B.D. Young, P. Sinclair, V. Rand, M.R. Teixeira, O. Joseph, B. Robinson, M. Maddison, N. Dastugue, P. Vandenberghe, C. Haferlach, P.J. Stephens, J. Cheng, P. Van Loo, M.R. Stratton, P.J. Campbell, and C.J. Harrison. 2014. Constitutional and somatic rearrangement of chromosome 21 in acute lymphoblastic leukaemia. Nature 508(7494): 98–102. doi:10.1038/nature13115 .
16. Liu, L., Z. Yan, B.A. Osia, J. Twarowski, L. Sun, J. Kramara, R.S. Lee, S. Kumar, R. Elango, H. Li, W. Dang, G. Ira, and A. Malkova. 2021. Tracking break-induced replication shows that it stalls at roadblocks. Nature 590(7847): 655–659. doi:10.1038/s41586-020-03172-w .
17. Ma, J., A. Ratan, B.J. Raney, B.B. Suh, W. Miller, and D. Haussler. 2008. The infinite sites model of genome evolution. Proc Natl Acad Sci U S A 105(38): 14254–61. doi:10.1073/pnas.0805217105 .
18. Maciejowski, J., Y. Li, N. Bosco, P.J. Campbell, and T. de Lange. 2015. Chromothripsis and kataegis induced by telomere crisis. Cell 163(7): 1641–54. doi:10.1016/j.cell.2015.11.054 .
19. McVey, M., V.Y. Khodaverdian, D. Meyer, P.G. Cerqueira, and W.D. Heyer. 2016. Eukaryotic dna polymerases in homologous recombination. Annual Review of Genetics 50(Volume 50, 2016): 393–421. doi:https://doi.org/10.1146/annurev-

genet-120215-035243 .
20. Mendez-Dorantes, C., X. Zeng, J.A. Karlow, P. Schofield, S. Turner, J. Kalinowski, D. Denisko, E.A. Lee, K.H. Burns, and C.Z. Zhang. 2024. Chromosomal rearrangements and instability caused by the line-1 retrotransposon. bioRxiv: 2024.12.14.628481. doi:10.1101/2024.12.14.628481 .
21. Menghi, F., K. Inaki, X. Woo, P.A. Kumar, K.R. Grzeda, A. Malhotra, V. Yadav, H. Kim, E.J. Marquez, D. Ucar, P.T. Shreckengast, J.P. Wagner, G. MacIntyre, K.R. Murthy Karuturi, R. Scully, J. Keck, J.H. Chuang, and E.T. Liu. 2016. The tandem duplicator phenotype as a distinct genomic configuration in cancer. Proc Natl Acad Sci U S A 113(17): E2373–82. doi:10.1073/pnas.1520010113 .
22. Min, J., J. Zhao, J. Zagelbaum, J. Lee, S. Takahashi, P. Cummings, A. Schooley, J. Dekker, M.E. Gottesman, R. Rabadan, and J. Gautier. 2023. Mechanisms of insertions at a dna double-strand break. Mol Cell 83(14): 2434–2448 e7. doi:10.1016/j.molcel.2023.06.016 .
23. Mirman, Z., S. Cai, and T. de Lange. 2023. Cst/polalpha/primase-mediated fill-in synthesis at dsbs. Cell Cycle 22(4): 379–389. doi:10.1080/15384101.2022.2123886 .
24. Mirman, Z. and T. de Lange. 2020. 53bp1: a dsb escort. Genes Dev 34(1-2): 7–23. doi:10.1101/gad.333237.119 .
25. Moore, J.K. and J.E. Haber. 1996. Capture of retrotransposon dna at the sites of chromosomal double-strand breaks. Nature 383(6601): 644–6. doi:10.1038/383644a0 .
26. Ramsden, D.A., J. Carvajal-Garcia, and G.P. Gupta. 2022. Mechanism, cellular functions and cancer roles of polymerase-theta-mediated dna end joining. Nat Rev Mol Cell Biol 23(2): 125–140. doi:10.1038/s41580-021-00405-2 .
27. Saini, N., S. Ramakrishnan, R. Elango, S. Ayyar, Y. Zhang, A. Deem, G. Ira, J.E. Haber, K.S. Lobachev, and A. Malkova. 2013, September. Migrating bubble during break-induced replication drives conservative dna synthesis. Nature 502(7471): 389–392. doi:10.1038/nature12584 .
28. Setton, J., K. Hadi, Z.N. Choo, K.S. Kuchin, H. Tian, A. Da Cruz Paula, J. Rosiene, P. Selenica, J. Behr, X. Yao, A. Deshpande, M. Sigouros, J. Manohar, J.T. Nauseef, J.M. Mosquera, O. Elemento, B. Weigelt, N. Riaz, J.S. Reis-Filho, S.N. Powell, and M. Imielinski. 2023. Long-molecule scars of backup dna repair in brca1- and brca2-deficient cancers. Nature 621(7977): 129–137. doi:10.1038/s41586-023-06461-2 .
29. Shale, C., D.L. Cameron, J. Baber, M. Wong, M.J. Cowley, A.T. Papenfuss, E. Cuppen, and P. Priestley. 2022. Unscrambling cancer genomes via integrated analysis of structural variation and copy number. Cell Genom 2(4): 100112. doi:10.1016/j.xgen.2022.100112 .
30. Smith, C.E., A.F. Lam, and L.S. Symington. 2009. Aberrant double-strand break repair resulting in half crossovers in mutants defective for rad51 or the dna polymerase delta complex. Mol Cell Biol 29(6): 1432–41. doi:10.1128/MCB.01469-08 .
31. Stephens, P.J., C.D. Greenman, B. Fu, F. Yang, G.R. Bignell, L.J. Mudie, E.D. Pleasance, K.W. Lau, D. Beare, L.A. Stebbings, S. McLaren, M.L. Lin, D.J. McBride, I. Varela, S. Nik-Zainal, C. Leroy, M. Jia, A. Menzies, A.P. Butler, J.W. Teague, M.A. Quail, J. Burton, H. Swerdlow, N.P. Carter, L.A. Morsberger, C. Iacobuzio-Donahue, G.A. Follows, A.R. Green, A.M. Flanagan, M.R. Stratton, P.A. Futreal, and P.J. Campbell. 2011. Massive genomic rearrangement acquired in a single catastrophic event during cancer development. Cell 144(1): 27–40. doi:10.1016/j.cell.2010.11.055 .
32. Stephens, P.J., D.J. McBride, M.L. Lin, I. Varela, E.D. Pleasance, J.T. Simpson, L.A. Stebbings, C. Leroy, S. Edkins, L.J. Mudie, C.D. Greenman, M. Jia, C. Latimer, J.W. Teague, K.W. Lau, J. Burton, M.A. Quail, H. Swerdlow, C. Churcher, R. Natrajan, A.M. Sieuwerts, J.W. Martens, D.P. Silver, A. Langerod, H.E. Russnes, J.A. Foekens, J.S. Reis-Filho, L. van 't Veer, A.L. Richardson, A.L. Borresen-Dale, P.J. Campbell, P.A. Futreal, and M.R. Stratton. 2009. Complex landscapes of somatic rearrangement in human breast cancer genomes. Nature 462(7276): 1005–10. doi:10.1038/nature08645 .
33. Stroik, S., J. Carvajal-Garcia, D. Gupta, A. Edwards, A. Luthman, D.W. Wyatt, R.L. Dannenberg, W. Feng, T.A. Kunkel, G.P. Gupta, M. Hedglin, R. Wood, S. Doublié, E. Rothenberg, and D.A. Ramsden. 2023, November. Stepwise requirements for polymerases δ and θ in theta-mediated end joining. Nature 623(7988): 836–841. doi:10.1038/s41586-023-06729-7 .
34. Teng, S.C., B. Kim, and A. Gabriel. 1996. Retrotransposon reverse-transcriptase-mediated repair of chromosomal breaks. Nature 383(6601): 641–4. doi:10.1038/383641a0 .
35. Tourdot, R.W., G.J. Brunette, R.A. Pinto, and C.Z. Zhang. 2021. Determination of complete chromosomal haplotypes by bulk dna sequencing. Genome Biol 22(1): 139. doi:10.1186/s13059-021-02330-1 .
36. Umbreit, N.T., C.Z. Zhang, L.D. Lynch, L.J. Blaine, A.M. Cheng, R. Tourdot, L. Sun, H.F. Almubarak, K. Judge, T.J.

Mitchell, A. Spektor, and D. Pellman. 2020. Mechanisms generating cancer genome complexity from a single cell division error. Science 368(6488). doi:10.1126/science.aba0712 .

37. Verma, P. and R.A. Greenberg. 2016, May. Noncanonical views of homology-directed dna repair. Genes and Development 30(10): 1138–1154. doi:10.1101/gad.280545.116 .
38. Willis, N.A., R.L. Frock, F. Menghi, E.E. Duffey, A. Panday, V. Camacho, E.P. Hasty, E.T. Liu, F.W. Alt, and R. Scully. 2017. Mechanism of tandem duplication formation in brca1-mutant cells. Nature 551(7682): 590–595. doi:10.1038/nature24477 .
39. Yu, Y., N. Pham, B. Xia, A. Papusha, G. Wang, Z. Yan, G. Peng, K. Chen, and G. Ira. 2018. Dna2 nuclease deficiency results in large and complex dna insertions at chromosomal breaks. Nature 564(7735): 287–290. doi:10.1038/s41586-018-0769-8 .
40. Zhang, C.Z., A. Spektor, H. Cornils, J.M. Francis, E.K. Jackson, S. Liu, M. Meyerson, and D. Pellman. 2015. Chromothripsis from dna damage in micronuclei. Nature 522(7555): 179–84. doi:10.1038/nature14493 .
